# Genome-wide activation of latent donor splice sites in stress and disease

**DOI:** 10.1093/nar/gks834

**Published:** 2012-09-23

**Authors:** Yuval Nevo, Eyal Kamhi, Jasmine Jacob-Hirsch, Ninette Amariglio, Gideon Rechavi, Joseph Sperling, Ruth Sperling

**Affiliations:** ^1^Department of Genetics, The Hebrew University of Jerusalem, Jerusalem 91904, ^2^Department of Organic Chemistry, The Weizmann Institute, Rehovot 76100 and ^3^Sheba Cancer Research Center, Sheba Medical Center, Ramat-Gan 52620, ^4^Sackler School of Medicine, Tel Aviv University, Israel

## Abstract

Sequences that conform to the 5′ splice site (5′SS) consensus are highly abundant in mammalian introns. Most of these sequences are preceded by at least one in-frame stop codon; thus, their use for splicing would result in pre-maturely terminated aberrant mRNAs. In normally grown cells, such intronic 5′SSs appear not to be selected for splicing. However, under heat shock conditions aberrant splicing involving such latent 5′SSs occurred in a number of specific gene transcripts. Using a splicing-sensitive microarray, we show here that stress-induced (e.g. heat shock) activation of latent splicing is widespread across the human transcriptome, thus highlighting the possibility that latent splicing may underlie certain diseases. Consistent with this notion, our analyses of data from the Gene Expression Omnibus (GEO) revealed widespread activation of latent splicing in cells grown under hypoxia and in certain cancers such as breast cancer and gliomas. These changes were found in thousands of transcripts representing a wide variety of functional groups; among them are genes involved in cell proliferation and differentiation. The GEO analysis also revealed a set of gene transcripts in oligodendroglioma, in which the level of activation of latent splicing increased with the severity of the disease.

## INTRODUCTION

Recognition of a 5′ splice site (5′SS) consensus sequence is a key step in both constitutive and alternative splicing (AS) (1). In addition to the 5′SSs that are actually used for splicing (authentic 5′SSs), sequences that conform to the 5′SS consensus are highly abundant in mammalian introns; yet, in normally growing cells, such intronic 5′SSs appear not to be selected for splicing and are thus termed latent (2). Importantly, most (>98%) of these alternative latent 5′SSs are preceded by at least one stop codon in the reading frame of the upstream exon. Thus, if activation of latent splicing occurred, the resulting alternatively spliced mRNA isoforms would become pre-maturely terminated aberrant mRNAs (2–5).

AS is a major source of the diversity of the human proteome and plays a major role in the regulation of gene expression. It is estimated that the majority of human genes undergo regulated AS (6–11) and, importantly, alterations in AS and misregulation of factors affecting AS were shown to be involved in several human diseases including cancer (12–16). In this study, we focus on the scope and possible consequences of activation of latent splicing, because we found, in a number of specific gene transcripts, that latent splicing, leading to aberrant mRNAs, could be elicited under stress (5,17,18).

The cellular stress response is of extreme significance to the survival of an organism. Response to different stimuli varies from manipulation of specific proteins, alteration of cell cycle control and up to a general transcriptional response (19). Of key interest is the process of pre-mRNA splicing and, especially, AS (1,20–23). The pivotal role of AS in the regulation of the transcriptome has suggested that cellular responses to environmental stimuli would also be manifested in changes in splicing and AS. Indeed, earlier studies in yeast showed that pre-mRNA splicing was inhibited by heat shock (24,25), and more recent studies have shown that heat treatment also caused changes in AS (26,27). In fact, RNA splicing and AS can be affected by different stress conditions such as temperature, UV radiation and neuronal stress (26,28,29). We reported heat-induced changes in AS of transcripts encoding the CAD enzyme (abbreviated for: carbamoyl-phosphate synthetase, aspartate trans-carbamylase, dihydroorotase) in Syrian hamster cells (17). As expected (24,25), subjecting these cells to mild heat shock partially inhibited normal splicing. At the same time, however, the heat shock elicited an apparently novel splicing event using an alternative latent 5′SS located within the downstream intron, thereby generating a spliced isoform having an extended exon. Interestingly, the latent 5′SS conformed to the consensus better than the upstream authentic one; yet, splicing at that site could not be detected in normally growing cells (17). A subsequent survey of 446 constitutively spliced human genes revealed that latent 5′SSs, whose sequences comply with the 5′SS consensus but are otherwise not used for splicing, are highly abundant within the introns of that database (2). This observation led us to ask whether activation of latent alternative 5′SSs is a general stress-induced phenomenon.

Here, we performed bioinformatics analyses searching for latent 5′SSs in the whole human genome. We show that >70% of the introns within the coding regions of human genes harbor multiple latent 5′SSs. Importantly, the intronic sequences upstream of almost all of these sites (>98%) harbor at least one in-frame stop codon. Thus, eliciting splicing at the latent 5′SSs (hereafter referred to as latent splicing) has the potential of introducing premature termination codons (PTCs) into the alternatively spliced isoform. To complement these bioinformatics predictions, we carried out microarray analyses to investigate the genome-wide impact of heat shock on latent splicing. We show that intronic latent 5′SSs are activated by heat shock treatment in hundreds of gene transcripts. Furthermore, analyses of available microarray data from the Gene Expression Omnibus (GEO) show that activation of latent splicing occurs under a wide range of stressful conditions spanning from hypoxia to different cancerous states, affecting thousands of gene transcripts.

## MATERIALS AND METHODS

### Plasmids

CAD1 and CAD2 minigene constructs were described previously (3). All plasmids were verified by DNA sequencing.

### Transfections and RNA isolation

Human 293 T cells were grown to 20–40% confluency in tissue culture plates and transiently co-transfected (30) with the appropriate constructs as described (3,31). Cells were harvested at 24 h post-transfection and total cellular RNA was extracted with guanidinium thiocyanate as described (3).

### Stress and antibiotic treatments

Treatment with cycloheximide (CHX; 20 µg/ml) was carried out for 4 h unless otherwise indicated. Treatments for the different stress conditions were as follows: hypoxia, induced using 500 µM DFO for 24 h; γ radiation, cells were exposed to 30 Gy and left to recover for 3 h; cold shock, cells were incubated at 10°C for 4 h; heat shock, cells were incubated at 42–45°C in a water bath for the indicated time and left to recover for 45 min where specified.

### RT–PCR and qPCR

Total cellular RNA was treated with RNase-free DNase I (50 U/ml; Promega) and cDNA was synthesized from 2 µg of RNA, using dT_15_ primer and MMLV reverse transcriptase (Promega). PCRs (20 µl) contained cDNA synthesized from 0.2 µg of total RNA, 10 pmol of the relevant primers and 1.0 U of Taq DNA polymerase (Promega). qPCR conditions for endogenous latent mRNA level of CAD in 165-28 Syrian hamster cells were previously described (31). The results represent at least three independent experiments. Primer sequences and cycling conditions are detailed in Supplementary Tables S5 and S6, respectively.

### Bioinformatics search for latent 5′SSs

Intronic 9-mer sequences were predicted as latent 5′SSs when they conformed to the 5′SS consensus sequence, namely were at least ∼164-fold more likely to arise from the 5′SSs sequence distribution than from a random intronic sequence. A PSSM was created from the authentic 5′SSs in our filtered database and was used for this calculation (Supplementary Table S1). A score was calculated for each predicted latent 5′SS as the percentage along the range of all possible scores as described (32). Characteristics of each predicted latent site were recorded (e.g. chromosomal location, intron number, intronic position, and score; see Supplementary Table S2). Predicted latent 5′SSs that were positioned <1000 nt downstream of the authentic 5′SS and had a PTC in the reading frame of the upstream exon were selected for further analyses with microarray data (see below).

### Microarray analysis

RNA was amplified and hybridized to Affymetrix Human Exon 1.0 ST Array according to the manufacturer’s instructions. Microarrays were hybridized, washed, stained and were scanned with the Affymetrix system (GeneChip Hybridization oven 640; GeneChip Fluidic Station 450; GeneChip scanner 3000). The GeneChip Operating Software was used to create raw signal data (CEL files).

Raw data were normalized and summarized using the ‘apt-probeset-summarize’ program of the Affymetrix Power Tools software package. The prepackaged analysis ‘rma’ was used for quantile normalization, background correction and median-polish summarization. The program was given a modified set of probesets definition files (PGF and MPS files), where cross-hybridizing probesets were excluded and all probes against relevant features (e.g. a latent exon) were united as one probeset. Summation was done both at the exon (probeset) and the gene levels, where gene assignment was done according to the chromosomal location of the probeset, comparing to genes’ location in the UCSC genome browser (March 2006) (http://genome.ucsc.edu). Gene-level summation was done using ‘core’ probesets only. Data were filtered to include only expressed genes by applying a threshold—only genes with a 3.6 expression and above were kept for the analyses, leaving ∼90% of all genes (transcript cluster ids) and ∼92% of all probesets.

Array data were then crossed with a database of predicted latent 5′SSs (see above). Predicted latent sites were intronic sequences that conform to the 5′SS consensus, ∼164-fold more likely to arise from the 5′SS sequence distribution than from a random intronic sequence, located at the most at 1000 nt downstream to the authentic 5′SS and introduce a PTC upon activation. The expression of latent exons was normalized to the expression of their upstream authentic exon and compared between treatment and control.

A similar analysis was done with modified PGF and MPS files, where probes were united as one probeset according to known alternative 5′SS events (UCSC genome browser, March 2006). Statistical significance was calculated using an equal proportions test between the results for latent 5′SSs and known alternative 5′SS events. Corrections for multiple hypothesis testing (MHT) did not affect the significance of the results. Figures for the expression of specific genes (Figure 3) were generated by using Partek Genomics Suite (6.4) with pre-processed microarray data.

### GEO data analyses

CEL files of the following experiments were obtained from the GEO database: GSE12546, GSE19154, GSE20342, GSE21840 and GSE9385. Analysis was done for each experiment according to the workflow described earlier. For the breast cancers overlap analyses (Figure 5A), raw expression data from MCF-7 cells of three different sources (GSE19154, GSE20342 and GSE21840) were analyzed together with data from MCF-10 A cells (GSE19154). A short script was written in order to calculate the exact representation factor and *P*-value of any overlap between any number of sets with specific sizes, given the size of the whole world.

## RESULTS

### Stress elicits latent splicing—the case of the CAD gene

**Figure 1.**
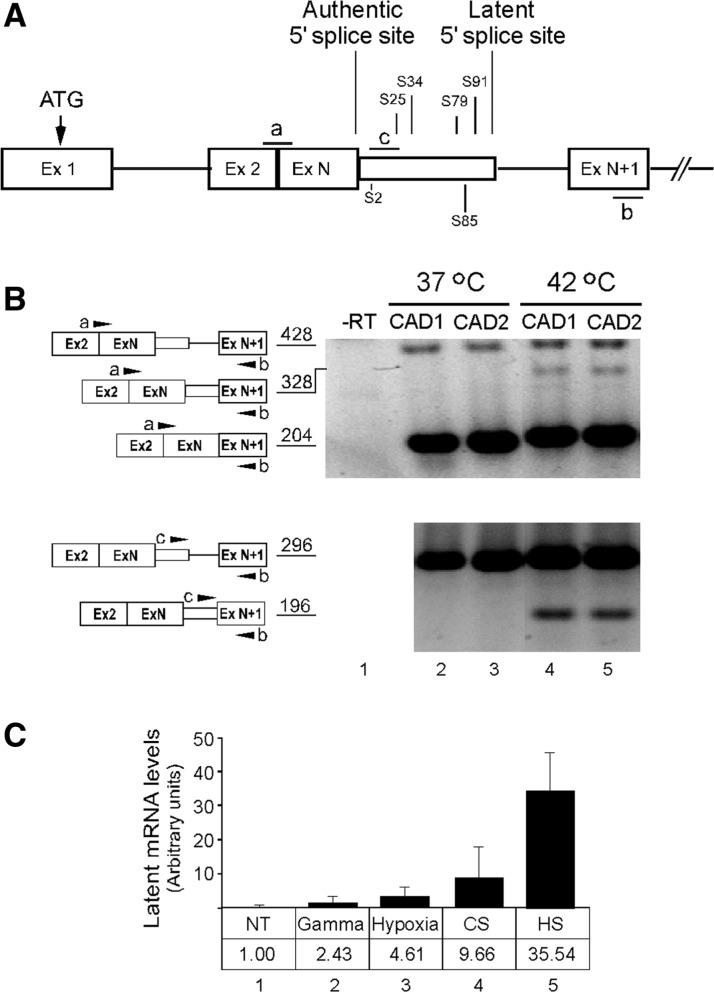
Latent splicing is elicited under stress conditions—the CAD case. (**A**) Schematic representation of CAD1 and CAD2 minigene constructs. Both constructs share the same start codon (ATG) but differ in the position of the stop codons between the authentic and latent 5′SSs (designated S; numbers represent distance in nucleotide from the authentic 5′SS; stop codons for CAD1 and CAD2 are marked above and below the latent exon, respectively). Open boxes, exons; lines, introns; narrow box, latent exon; a, b, c, mark the position of the PCR primers. (**B**) RT–PCR analysis of RNA extracted from human 293 T cells transiently transfected with either CAD1 or CAD2 and grown at 37°C (lanes 1–3) or heat-treated at 42°C for 45 min (lanes 4–5). PCRs were performed with primer pair a + b (upper panel) and primer pair b + c (lower panel). The PCR products and their sizes (nt) are depicted on the left. (**C**) Relative levels of latent CAD mRNAs expressed from the endogenous CAD gene in 165-28 cells in response to different environmental stress conditions. NT, no treatment; Gamma, 30 Gy; Hypoxia, 500 µM DFO; CS, cold shock (10°C for 4 h); HS, heat shock (45°C for 45 min). All measurements were normalized to the basal level detected for the control of untreated cells (designated as 1.00). Error bars represent standard errors of three to five independent experiments.

Activation of splicing at an intronic latent 5′SS (Figure 1A) was first observed in transcripts of the endogenous CAD gene in Syrian hamster cells subjected to heat shock stress (17). It was also noted that the sequence flanked by the latent 5′SS and the upstream authentic 5′SS contained four stop codons in the CAD protein reading frame. Here we tested the effect of heat shock on the splicing pattern of two CAD minigene constructs, CAD1 and CAD2 (Figure 1A), which were transfected into human 293 T cells. CAD1 and CAD2 had four and two in-frame stop codons, respectively, between the authentic and the latent 5′SSs. RT–PCR analyses revealed that latent splicing could not be observed under normal growth conditions (Figure 1B, lanes 2 and 3), whereas heat treatment elicited latent splicing in the mRNA expressed from both constructs (Figure 1B, lanes 4 and 5, respectively).

This finding raised the question whether other stresses would affect latent splicing in a similar manner. Using a qPCR protocol (31), we show that exposing Syrian hamster cells to γ irradiation, hypoxia, cold shock and heat shock elicited latent splicing in the endogenous CAD mRNA. It is evident that while latent splicing was elicited by all stress conditions tested, changes in temperature had the strongest effect (Figure 1C, columns 2–5). Exposure to cold shock or heat shock resulted in a respective increase of latent splicing by 10- or 35-fold relative to the basal level in un-treated cells. Latent splicing was significantly most sensitive to heat shock (*t*-test: *P* < 0.01), reaching in some instances 15% of the level of the authentic mRNA. Hypoxia and exposure to 30 Gy of γ-irradiation had a modest effect on latent splicing, amounting to a respective increase of latent splicing by 4- and 2-fold, relative to the basal level in un-treated cells.

### Heat shock elicits latent splicing—a global analysis

**Figure 2.**
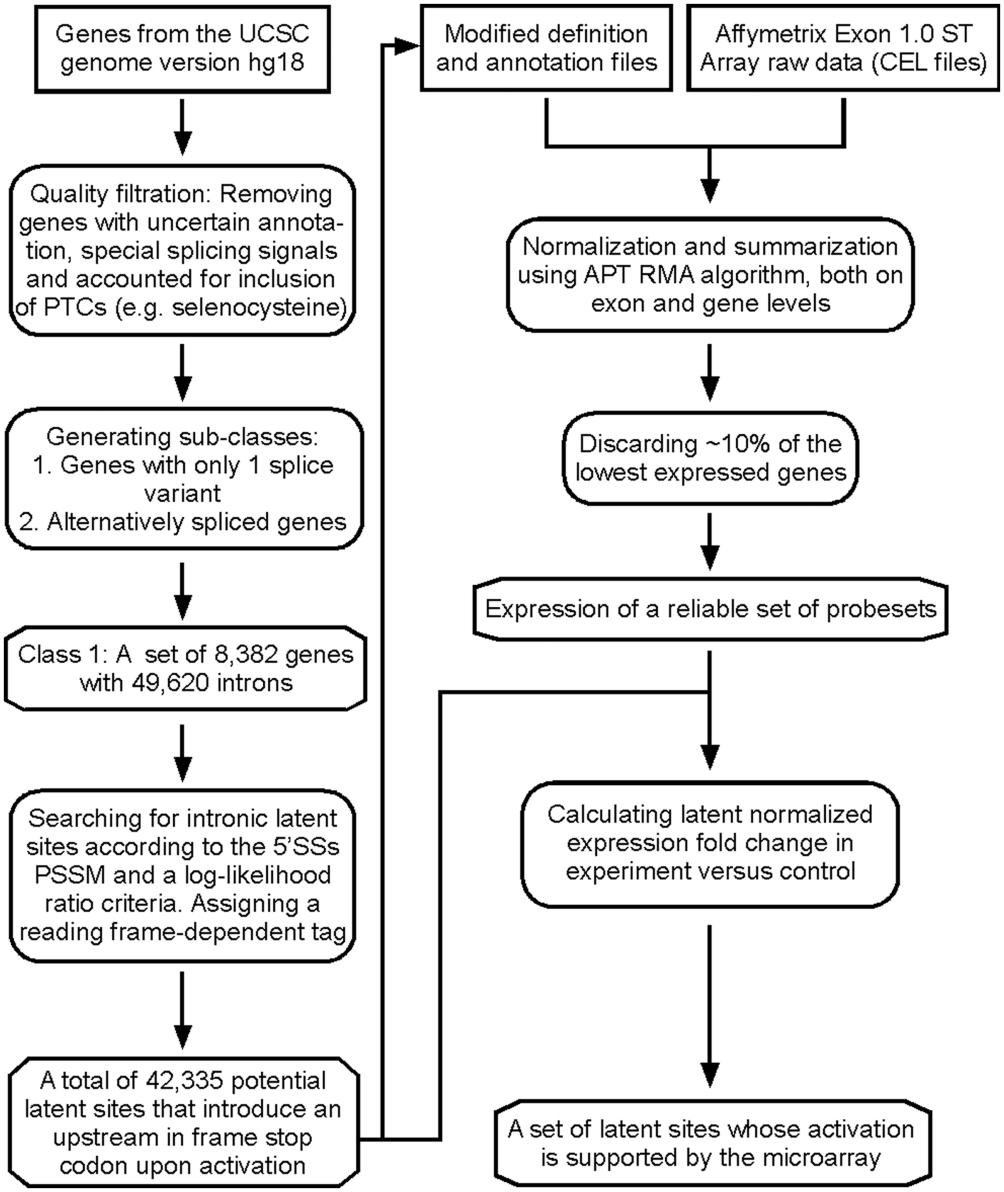
Latent 5′SSs are highly abundant in the human genome. Flow chart of global computational and experimental analyses of latent splicing. Boxes on the left describe the computational search for latent 5′SSs; boxes on the right describe the experimental chip-aided analysis of elevation of latent splicing after heat shock. Rectangles, program inputs; rounded rectangles, programs; octagons, program outputs; arrows, direction of information flow.

Having shown that heat stress elicited splicing at a latent alternative 5′SS in the endogenous CAD pre-mRNA transcript and in pre-mRNAs expressed in human cells transiently transfected with CAD minigene constructs, we went on to evaluate the generality of this effect in the human genome. Our previous bioinformatics analysis, which was performed on a data set of 446 genes with no restriction on the length of the latent exons (defined as the sequence between the authentic and the latent 5′SSs), revealed an average of 5–6 latent 5′SSs per intron (2). Here, we extended our computational analysis to the whole human genome using a more rigorous algorithm. Figure 2 (left) depicts a flow chart of the analysis. Our initial data set included the human genes from the UCSC genome version hg18, from which we filtered out genes with uncertain annotation, genes with special splicing signals and genes with PTCs encoding for selenocysteine. In order to minimize possible combinatorial effects and increase our confidence in the determined reading frame, we next filtered out alternatively spliced genes. Our final database included a set of 8382 genes having 49 620 introns (Table 1). Following the procedure of Shapiro and Senapathy (32), we searched this data set for intronic latent 5′SSs according to a log likelihood ratio criteria (Supplementary Table S1). This analysis revealed that ∼71% of the introns had latent 5′SSs and the total number of such sites was 344 709, of which 86% (295 835) had at least one in-frame stop codon upstream of the latent site. Taking into account only introns flanked by coding exons (excluding introns within untranslated regions) showed an average of nine latent 5′SSs per intron, with >98% of them having an upstream in-frame stop codon (Table 1). Because human exons are rather short, while introns can be very long (33), latent 5′SSs that are very far from the authentic 5′SS are unlikely to serve as alternative 5′SSs. Limiting our search to latent 5′SSs positioned at <1000 nt downstream from the authentic 5′SS and to introns in coding regions, we remained with 46 279 latent 5′SSs in 26 229 introns, in which the fraction of sites that harbored an upstream in-frame stop codon amounted to 91%. These computational analyses predicted the presence of latent 5′SSs in most human introns.

**Table 1.** Occurrences of latent 5′SSs in the human genome

Total number of genes^a^	Total number of introns	Introns with latent 5′SSs (%)	Total latent 5′SSs	Latent 5′SSs per intron	Latent 5′SSs with upstream PTC (%)
8382^b^	49 620	35 385 (71.31)	344 709^d^	9.74	295 835 (85.82)
6405^c^	46 131	32 614 (70.70)	299 780^d^	9.19	295 835 (98.68)
8382^b^	49 620	28 423 (57.28)	50 369^e^	1.77	42 335 (84.05)
6405^c^	46 131	26 229 (56.86)	46 279^e^	1.76	42 335 (91.48)

^a^After filtration (for details see text).

^b^Whole genes.

^c^Whole genes excluding intron-less genes and UTRs.

^d^All latent 5′SSs.

^e^Latent 5′SSs located up to 1000 nt downstream of the respective authentic 5′SS.

Because heat shock had the strongest effect on the activation of the latent alternative 5′SSs (Figure 1), we carried out a whole-genome analysis of latent splicing affected by this kind of stress. For this analysis, we used the Human Exon 1.0 ST Array (Affymetrix). The ∼1.4 × 10^6^ probesets of this platform include core probesets, which recognize the most established exons in the human genome, as well as many other probesets that recognize exons having a weaker level of evidence. Furthermore, non-core probesets can recognize AS at 242 137 latent 5′SSs, which constitute ∼70% of the latent sites identified by our bioinformatics analyses. This platform is thus suitable to perform whole-genome analyses of changes in splicing in response to stress.

For the chip-aided global analysis, total RNA was extracted from heat-treated and from untreated HEK 293 T cells. Our bioinformatics search revealed 42 335 latent sites at a distance of up to 1000 nt from the authentic 5′SS, which had an upstream stop codon. These latent sites were crossed with the Affymetrix probesets definition files to create modified definitions (PGF and MPS files), where each of the latent exon, its upstream exon and its downstream intron were defined as a single PSR (Figure 2, right panels). The signals of probesets against latent exons were normalized to the signals of the probesets against the respective upstream exons, and a comparison was made of the values obtained for the heat-treated and the untreated cells. A threshold of at least 1.5-fold change was taken as a measure of up- or downregulation of the latent exon. In cases where a probeset against the downstream intron existed, the change in signal of the latent exon had to be also at least 1.5-fold greater than that of the intronic area. This measure was taken in order to exclude cases of intron retention or general splicing inhibition. Eliminating from our analysis the ∼10% lowest expressed genes, we analyzed a subset of 4669 latent sites (Table 2). Comparing to the control data set of untreated cells, this analysis revealed that 508 (10.88%) latent exons had at least 1.5-fold elevated expression levels in response to heat shock (Supplementary Table S2). To assess the significance of this result, we performed an analogous analysis on 497 known cases of alternative 5′SS selection, occurring in genes that were filtered out from the analysis of latent splicing (Figure 2). This control experiment showed that only 24 (4.83%) of the cases of alternative 5′SS selection had changes in their splicing pattern due to heat shock (equal proportion test: *P* < 1.23 *e* − 05, MHT correction *P* < 1.11 *e* − 04). These analyses show that the observed changes in the splicing pattern of the 508 latent sites indicate a significant effect on the regulation of latent splicing due to the environmental stress and are probably a low estimate resulting from the stringent analysis. Table 3 depicts 30 representative genes from the list of 508 genes that showed elevated levels in latent splicing after heat shock. Our bioinformatics search revealed that latent 5′SSs are found in most genes, independent of their function. This is in accordance with the global analysis (Supplementary Table S2), showing that the list of genes in which latent splicing was activated by heat shock is widespread and includes genes with diverse key cellular functions.

**Table 2.** Number of 5′SSs that show elevated expression in heat shocked versus control cells

	Total sites	Upregulated sites in heat shocked cells (%)		
Threshold		1.5	1.75	2.0
Type of 5′SS				
Latent	4669	508 (10.88)	277 (5.93)	165 (3.53)
Alternative	497	24 (4.83)	11 (2.21)	6 (1.20)
*P*-value^a^		<1.23*e* − 05	<2.96*e* − 04	<2.93*e* − 03

^a^Equal proportion test.

**Table 3.** Representative upregulated latent 5′SSs

Gene symbol	Gene name	Location of latent 5′SS	Fold change
SLC10A1	Solute carrier family 10 (sodium/bile acid cotransporter family), member 1	chr14:69332857	32.46
TMED10	Transmembrane emp24-like trafficking protein 10 (yeast)	chr14:74672120	4.95
FGD1	FYVE, RhoGEF and PH domain containing 1	chrX:54490201	3.51
C1R	Complement component 1, r subcomponent	chr12:7132902	2.97
M6PR	Mannose-6-phosphate receptor (cation dependent)	chr12:8987217	2.68
VIPR2	Vasoactive intestinal peptide receptor 2	chr7:158595119	2.48
SDPR	Serum deprivation response	chr2:192419119	2.47
GTPBP4	GTP binding protein 4	chr10:1032404	2.36
RAD21	RAD21 homolog (*Schizosaccharomyces pombe*)	chr8:117947543	2.27
ASF1B	ASF1 anti-silencing function 1 homolog B (*Saccharomyces cerevisiae*)	chr19:14093048	2.17
HPRT1	Hypoxanthine phosphoribosyltransferase 1	chrX:133455594	2.11
MYOM2	Myomesin (M-protein) 2, 165 kDa	chr8:2034601	2.09
KISS1	KiSS-1 metastasis-suppressor	chr1:202428038	2.05
KIF5A	Kinesin family member 5 A	chr12:56261752	1.99
ACBD3	Acyl-CoA binding domain containing 3	chr1:224408138	1.96
LBP	Lipopolysaccharide binding protein	chr20:36413711	1.94
PIM2	Pim-2 oncogene	chrX:48657033	1.89
CA2	Carbonic anhydrase II	chr8:86564319	1.82
NUP35	Nucleoporin 35 kDa	chr2:183731529	1.78
LOXL1	Lysyl oxidase-like 1	chr15:72027052	1.74
C19orf30	Chromosome 19 open reading frame 30	chr19:4721230	1.73
SCNN1B	Sodium channel, non-voltage-gated 1, beta	chr16:23272568	1.72
HDDC2	HD domain containing 2	chr6:125639799	1.71
ALG14	Asparagine-linked glycosylation 14 homolog (*Saccharomyces cerevisiae*)	chr1:95310401	1.70
PPM1D	protein phosphatase, Mg^2+^/Mn^2+^ dependent, 1D	chr17:56089182	1.69
NDUFAB1	NADH dehydrogenase (ubiquinone) 1, alpha/beta subcomplex, 1, 8 kDa	chr16:23503681	1.67
KIF7	Kinesin family member 7	chr15:87974010	1.63
RND1	Rho family GTPase 1	chr12:47545414	1.61
RAB6B	RAB6B, member RAS oncogene family	chr3:135041993	1.59
ZNF330	Zinc finger protein 330	chr4:142373854	1.57

**Figure 3.**
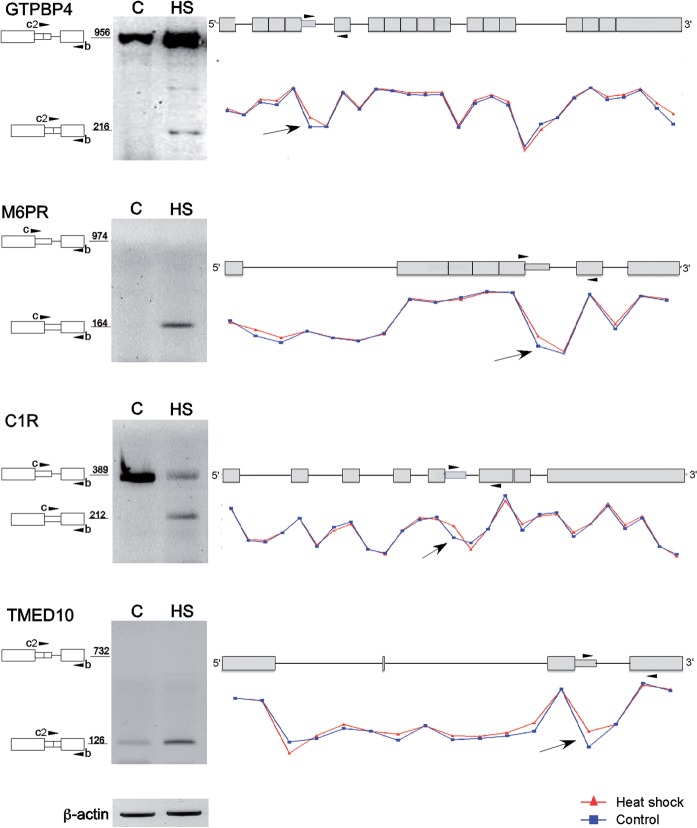

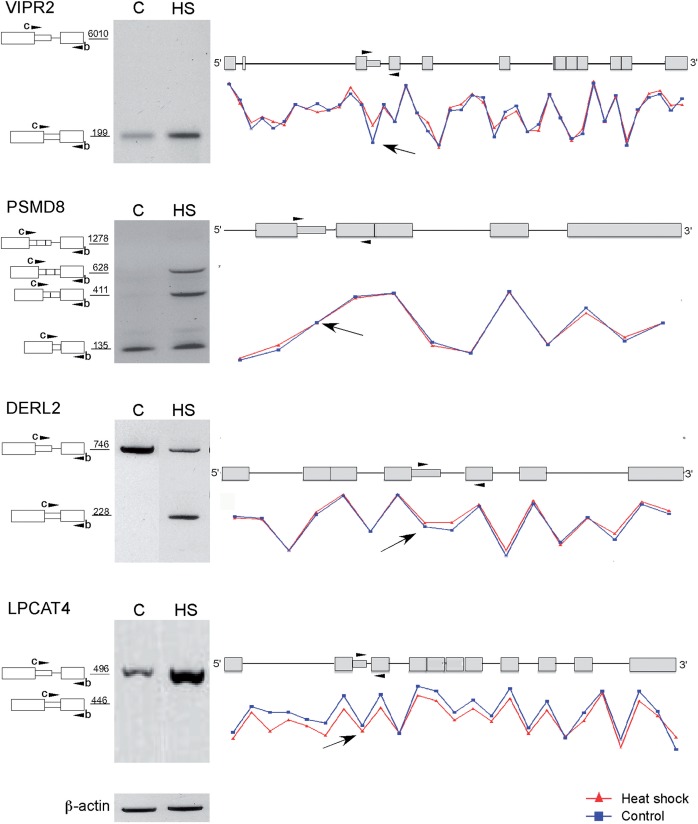
Validation, by RT–PCR, of microarray-detected heat-induced elevation in the level of latent splicing of mRNAs expressed from eight genes in HEK 293 T cells. Right panels: Curves corresponding to the levels of probesets signals along the gene were created using the Partek Genomics Suite software (red, heat-treated cells; blue, untreated cells). Arrows point at probesets that recognized latent exons. Schematics of the genes are drawn above the expression curves. Boxes, exons; lines, introns; narrow box, latent exon; arrowheads mark the positions of the PCR primers, which are identical to the primers indicated on the left panel. Left panels: RT–PCR analyses with primer-pairs flanking the latent sites, as indicated in the schematic drawings of the PCR products on the left (numbers indicate sizes of the PCR products). GTPBP4, GTP-binding protein 4; M6PR, mannose-6-phosphate receptor; C1R, complement component 1, r subcomponent; TMED10, transmembrane emp24-like trafficking protein 10; VIPR2, vasoactive intestinal peptide receptor 2; PSMD8, proteasome 26 S subunit, non-ATPase, 8; DERL2, derlin 2; and LPCAT4, lysophosphatidylcholine acyltransferase 4; C, untreated cells; HS, heat-treated cells. All PCR products were verified by sequencing.

For validation of the microarray results, we selected eight genes. Five of them (GTPBP4, M6PR, C1R, TMED10 and VIPR2) were identified by our bioinformatics survey, and the microarray analysis revealed heat-induced elevation of latent splicing. For these five gene transcripts, validation by RT–PCR analyses showed that latent splicing was significantly elevated in response to heat shock treatment as compared to the control cells (Figure 3). Two other interesting cases were the PSMD8 and DERL2 genes. The bioinformatics analysis identified their transcripts as containing latent exons, but they showed only a modest elevation (DERL2) and no elevation (PSMD8) of latent splicing in the microarray. RT–PCR analysis, however, revealed heat-induced activation of a latent 5′SS in intron 4 of DERL2 and of two latent 5′SSs in the first intron of PSMD8 (Figure 3). Interestingly, activation of latent splicing in DERL2 and PSMD8, under another stress condition, was revealed by a microarray analysis (data not shown), consistent with the bioinformatics analysis. This highlights the observation that different stress conditions elicit different levels of latent splicing (see ‘Discussion’ section). We also identified a novel alternative 5′SS in PSMD8, which did not have an upstream stop codon, and splicing from this site was observed with and without heat treatment (Figure 3). These examples suggest that the list of 508 genes that showed heat-induced elevated levels of latent splicing in our microarray analysis were probably an underestimate. In case of the LPCAT4 transcript, the RT–PCR analysis could not detect any expression of latent RNA, although the microarray analysis showed elevated levels of expression of the latent exon. This discrepancy can be explained due to a more substantial decrease in the expression level of the upstream exon that was used for normalization (Figure 3).

### Heat-induced latent mRNAs are subject to NMD

**Figure 4.**
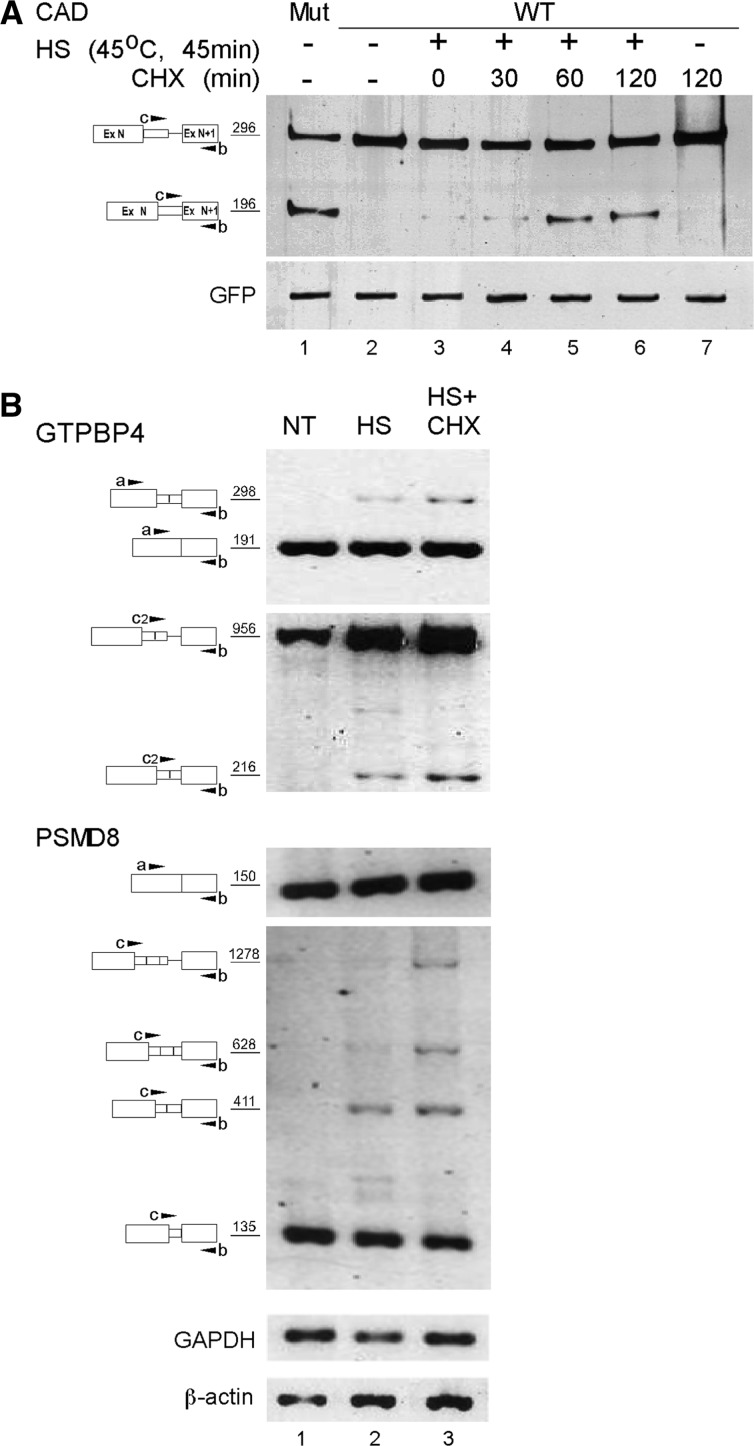
Latent mRNAs that escape suppression of splicing are subjected to the NMD pathway. (**A**) HEK 293 T cells were co-transfected with the WT CAD1 plasmid (lanes 2–7) and GFP. Twenty-four hours post-transfection, cells were heated at 45°C for 45 min, followed by treatment with CHX as indicated. Total RNA was isolated and analyzed by RT–PCR. To mark the location of latent mRNA, we show the splicing pattern of CAD mRNA expressed from a mutant construct lacking in-frame stop codons upstream of the latent site (lane 1). Note that treatment with CHX for 120 min without heat shock did not elicit latent splicing (lane 7). (**B**) RT–PCR analysis of the splicing patterns of RNAs expressed from the endogenous genes GTP binding protein 4 (GTPBP4) and proteasome 26 S subunit non-ATPase 8 (PSMD8) in untreated HEK 293 T cells (NT); heat-treated cells (HS) and cells treated with both heat and CHX (HS + CHX). The levels of GAPDH and β-actin were used as a loading control. The PCR products and their sizes (nt) are depicted on the left.

In previous studies, we have shown that the apparent lack of expression of latent mRNAs under normal growth conditions could not be attributed to degradation by NMD or any other NMD-like RNA surveillance mechanism [(5) and summarized in Supplementary Table S1 of (34)]. Our data have suggested an alternative model, whereby splicing involving latent sites is suppressed such that the generation of PTC-bearing mRNAs is avoided (3,18,31,34). However, we see here that subjecting cells to stresses appeared to have abrogated this suppression, leading to the generation of latent mRNAs. The PTC-bearing latent mRNAs thus formed would likely become substrates for downregulation by NMD. Indeed, when heat shocked cells were treated with CHX, which is known to inhibit NMD by translational block (35), the level of latent, PTC-harboring, CAD WT mRNAs was upregulated in a time-dependent manner (Figure 4A, compare lane 3 to lanes 4–6). As already shown, under normal growth conditions, the expression of latent CAD mRNA could not be detected (Figure 4A, lane 2). As controls we show that a CAD mutant lacking in-frame stop codons did express latent CAD mRNA (Figure 4A, lane 1) and that treatment of cells expressing the wild-type CAD with CHX, but without prior heat-treatment, did not elicit latent splicing (Figure 4A, lane 7). Figure 4 also shows that the NMD pathway was not affected by the heat treatment, because after heat shock we could still inhibit NMD using CHX. This is consistent with a previous report showing that heat shock did not affect NMD (36).

The same effect was further demonstrated using two endogenously expressed pre-mRNA transcripts, GTPBP4 and PSMD8, both of which were computationally identified as targets of suppression of splicing (SOS) and were also validated as such by RT–PCR analysis. Here again, expression of the latent mRNAs in untreated cells was not detected (Figure 4B, lane 1) and heat shock treatment gave rise to latent mRNAs (Figure 4B, lane 2), whose levels increased after treatment with CHX (Figure 4B, lane 3). The significance of this experiment is 2-fold. First, it ascribes an additional function to NMD as a fail-safe mechanism when latent splicing is elicited. Second, it points to the fact that the observed levels of the stress-induced latent splicing are low estimates because splicing events at the latent alternative 5′SSs introduce PTCs into the latent mRNAs and apparently render them substrates for NMD.

### Activation of latent splicing in stress and disease

Our bioinformatics studies have shown that thousands of latent 5′SSs are present in introns and yet they are not utilized for splicing under normal growth conditions. We have shown here that the tight SOS from the latent sites can be abrogated by stress, not only in the case of a number of minigene transcripts but also in hundreds of endogenous genes. These findings raised the possibility that SOS from latent sites might be abrogated in other stresses and in disease. To explore this possibility, we used publicly available Human Exon 1.0 ST Array expression data from the GEO. We analyzed the activation of latent splicing in disease and under stress conditions in a number of systems, employing the procedure developed for the analysis of latent splicing in our heat-stressed cells (Table 4). When we analyzed transcripts of human umbilical vein endothelial cells under conditions that mimic hypoxia stress compared to non-stressed cells (37), we found that in 732 (15.79%) latent 5′SSs the level of expression was elevated >1.5-fold under hypoxia (*P* < 2.22 *e* − 04; MHT correction, *P* < 2.00 *e* − 03). Significant results were obtained also for more stringent thresholds (Table 4).

**Table 4.** Occurrences of latent splicing in stress conditions and diseases

Stress or disease	Cells (treatment)	GEO accession	Event type	Number of events	Upregulation versus control^a^ (%)		
					*t* = 1.5^b^	*t* = 1.75	*t* = 2.0
Hypoxia	HUVECs (cobalt chloride)	GSE12546	Latent sites	4636	732 (15.79)	429 (9.25)	282 (6.08)
			Alt. 5′SSs	498	49 (9.84)	27 (5.42)	15 (3.01)
			*P*-values		<2.22 × 10^−4^	<2.15 × 10^−3^	<2.65 × 10^−3^
Breast cancer	MCF-7 and MCF-10 A as control	GSE19154	Latent sites	4644	794 (17.10)	524 (11.28)	368 (7.92)
			Alt. 5′SSs	499	49 (9.82)	30 (6.01)	24 (4.81)
			*P*-values		<1.51 × 10^−5^	<1.54 × 10^−4^	<6.36 × 10^−3^
	MCF-7 (estrogen 6 h)	GSE20342	Latent sites	4678	293 (6.26)	133 (2.84)	61 (1.30)
			Alt. 5′SSs	502	29 (5.78)	13 (2.59)	7 (1.39)
			*P*-values		<0.334	<0.373	<0.568
	MCF-7 (estrogen 24 h)	GSE20342	Latent sites	4625	433 (9.36)	198 (4.28)	120 (2.59)
			Alt. 5′SSs	500	26 (5.20)	12 (2.40)	4 (0.80)
			*P*-values		<9.81 × 10^−4^	<0.022	<6.56 × 10^−3^
	MCF-7 (genotoxic stress response: camptothecin)	GSE21840	Latent sites	4636	783 (16.89)	513 (11.07)	345 (7.44)
			Alt. 5′SSs	495	24 (4.85)	12 (2.42)	9 (1.82)
			*P*-values		<1.34 × 10^−12^	<8.24 × 10^−10^	<1.36 × 10^−6^
Glioma	Glioblastoma	GSE9385	Latent sites	4662	409 (8.77)	259 (5.56)	178 (3.82)
			Alt. 5′SSs	504	26 (5.16)	18 (3.57)	14 (2.78)
			*P*-values		<2.76 × 10^−3^	<0.031	<0.121
	Oligodendroglioma II	GSE9385	Latent sites	4684	853 (18.21)	600 (12.81)	448 (9.56)
			Alt. 5′SSs	502	52 (10.36)	33 (6.57)	28 (5.58)
			*P*-values		<5.29 × 10^−6^	<2.5 *e* − 05	<1.64 × 10^−3^
	Oligodendroglioma III	GSE9385	Latent sites	4672	612 (13.10)	357 (7.64)	231 (4.94)
			Alt. 5′SSs	505	29 (5.74)	16 (3.17)	10 (1.98)
			*P*-values		<9.31 × 10^−7^	<1.11 × 10^−7^	<1.34 × 10^−3^

^a^Unless specified, the controls were untreated cells (hypoxia and breast cancer) and normal brain cells (glioma).

^b^*t*, threshold.

Next, we performed analyses of latent splicing activation comparing data from two different breast cell lines having different phenotypes: the immortalized, non-transformed mammary epithelial cell line MCF-10 A and the MCF-7 breast carcinoma cell line, which is expressing the estrogen receptor (ER) (38). Comparing MCF-7 breast cancer cells to MCF-10 A breast cells, our analyses revealed an elevation of over 1.5-fold in latent splicing in 794 (17.10%) latent sites. Treatment of MCF-7 cells with estrogen for 6 and 24 h was reported to affect AS (39). Our analyses of these data revealed that these treatments also elevated the level of latent splicing. Thus, latent splicing was elevated over 1.5-fold in 293 (6.26%) and in 433 (9.36%) latent sites after 6 or 24 h, respectively. Genotoxic stress of MCF-7 cells with camptothecin, which inhibits transcription elongation by creating topoisomerase I-DNA adducts that physically interfere with RNA pol II elongation, was shown to affect AS (40). We also found an effect on latent splicing, with 783 (16.89%) latent sites showing elevated levels of at least 1.5-fold. Analogous analyses performed on each of these data sets, analyzing known alternative 5′SSs (Table 4), showed that a much lower percentage of alternative 5′SSs were activated compared to the elevation in levels of latent splicing under the same conditions. Except for the estrogen treatment for 6 h, in all other cases *P*-values were <0.001 and reached <1.34*e* −12 for the MCF-7 breast cancer cells after genotoxic stress. All statistically significant results remained significant after MHT correction. These analyses revealed that the changes we saw in the pattern of latent splicing in the hypoxia-treated cells and the breast cancer cells, untreated and treated, are due to the stress and disease and are probably a low estimate, resulting from the stringent analysis.

We also analyzed the elevation of latent splicing in a number of gliomas—nervous system neoplasms derived from glial cells, using data available in the GEO database (41). We analyzed glioblastoma multiforme and oligodendroglioma Grades II and III that were shown to have changes in AS. Our analyses revealed that while in glioblastoma tumors 409 (8.77%) latent sites had over 1.5-fold elevation in latent splicing, in oligodendroglioma samples the number increased to 853 (18.21%) in Grade II and to 612 (13.10%) in Grade III (Table 4). As in the hypoxia and breast cancer cases, an analogous analyses of known alternative 5′SSs (Table 4) showed that the activation of latent splicing in gliomas was significantly higher than that of changes in alternative 5′SSs selection (*P* < 2.76 × 10^−3^ and remained significant after MHT corrections).

### Do stress-activated latent 5′SSs differ from those that were not activated?

Our global analyses revealed that thousands of latent 5′SSs are activated in stress and cancer. These results enabled us to search for characteristics of latent 5′SSs and especially to compare the latent sites that were activated in stress and cancer to those that were not. Our bioinformatics analyses did not show a significant difference in the length distribution of activated and non-activated latent exons (Supplementary Figures S1 and S2). Furthermore, comparison of the score and sequence pattern of the activated latent 5′SSs and of the non-activated ones did not show significant differences (Supplementary Figure S3). We can therefore conclude that the length of the latent exon and the score and sequence pattern of the latent 5′SS are not essential elements in determining whether a latent site is activated or not.

### Activated latent 5′SSs typical to specific cancerous states

**Figure 5.**
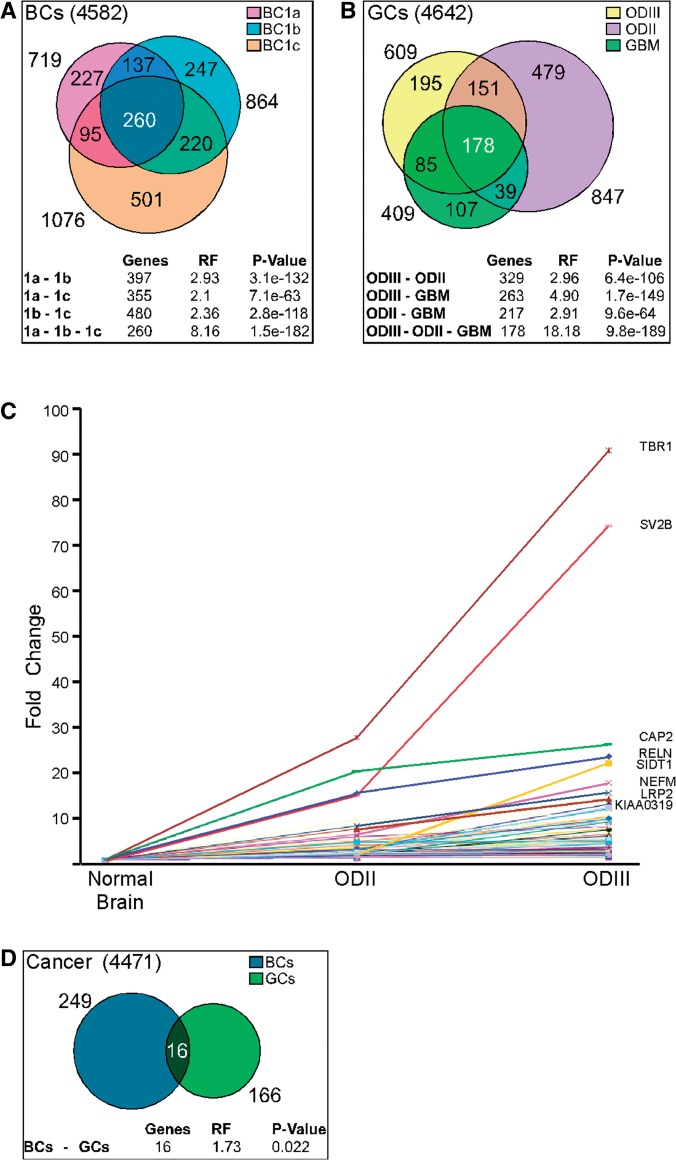
Overlap between activated latent 5′SSs in cancerous states and correlation between the level of latent splicing and the severity of oligodendroglioma. (**A**) Sets of activated latent 5′SSs in MCF-7 cells, taken from three different sources and compared to MCF-10 A cells, are represented by overlapping circles. BC1a, MCF-7 from (38); BC1b, MCF-7 from (40); BC1c, MCF-7 from (39). The sizes of the different intersections are given in their proper places, while the total size of each set is given outside its relevant circle. The table shows the significance of the intersections between each pair and between all three sets, compared to a random selection of equally sized sets. RF, representation factor, is the ratio between the observed size and the randomly expected one; *P*-value is the actual probability of having equally sized or larger intersections (at random selection). (**B**) A similar representation as in a, for the overlap between latent 5′SSs that are activated in glioblastoma (GBM), oligodendroglioma Grade II (ODII) and oligodendroglioma Grade III (ODIII), compared to normal brain (41). (**C**) Correlation between the level of activation of latent splicing and the severity of oligodendroglioma. The graph depicts fold-changes in the level of latent splicing in 125 gene transcripts whose latent splicing expression increased from oligodendroglioma Grade II to Grade III. Names of the top-scoring gene transcripts are indicated. (**D**) A similar representation as in (A) and (B), for the overlap between latent 5′SSs that are activated in breast cancer and in glial tumors.

To identify latent splicing events that are typical to a specific cancerous condition, we first analyzed the level of latent splicing in each of the three data sets from the GEO that had information on MCF-7 breast cancer cells (38–40) and compared it with the level of latent splicing in the MCF-10 A cells (38). Figure 5A depicts a Venn diagram of this analysis showing that in MCF-7 cells activation of latent splicing occurs in hundreds of genes, in all three data sources. While the overlap between the three experiments (and between each pair) is not complete, it still includes hundreds of genes and is highly significant. This overlap gives a minimal reliable set of 260 latent 5′SSs that are activated in MCF-7 breast cancer cells. These numbers represent 36% of the latent sites activated in MCF-7 cells compared to MCF-10 A cells (38), and the overlap between each pair ranges between 45% and 55%. The full list of latent 5′SSs that are activated in at least two experiments is given in Supplementary Table S3. Microarray-detected examples of elevation of latent splicing in MCF-7 cells compared to MCF-10 A cells are shown in Supplementary Figure S4. The genes that show activation of latent splicing in breast cancer cells represent a wide number of functional groups; among them are genes involved in cell proliferation and differentiation.

Similar analyses were performed on gliomas using data from the GEO (41). Figure 5B depicts a Venn diagram of latent splicing activation in the three gliomas and the overlap between them. The full list of latent 5′SSs that are activated in at least two gliomas is given in Supplementary Table S4. A microarray-detected example of a latent splice site that was activated in all three gliomas is shown in Supplementary Figure S5. The overlap between pairs ranges between 53% and 64%. Furthermore, a group of 178 latent 5′SSs showed activation of latent splicing in all these three cancerous cells. This overlap is highly significant. It represents 43% of the latent sites activated in glioblastoma and underlies a lower limit of a list of latent 5′SSs that are activated in gliomas.

As oligodendroglioma Grades II and III represent different stages of aggressiveness of the same malignant tumor, we next asked whether there are genes in which the level of activation of latent splicing increases in correlation with the severity of the disease, namely progressing from Grade II to Grade III. Our analysis showed that in 125 gene transcripts the level of activation of latent splicing was higher in the more aggressive oligodendroglioma Grade III compared to oligodendroglioma Grade II (Figure 5C). Of note is the gene transcript of T box brain 1 transcription factor (TBR1). This gene is a member of a conserved family of genes that share a common DNA-binding domain—the T-box—and encode transcription factors involved in the regulation of developmental processes. It showed ∼28-fold increase in the level of latent splicing in oligodendroglioma Grade II compared to normal cells and increased to ∼90-fold in oligodendroglioma Grade III. Another important example is the transcript of the gene SV2B—synaptic vesicle protein 2B—suggested to play a role in the control of regulated secretion in neural and endocrine cells. It showed ∼15-fold increase in the level of latent splicing in oligodendroglioma Grade II compared to normal cells, increasing to ∼74-fold in oligodendroglioma Grade III. Three additional gene transcripts, CAP2, RELN and SIDT1, showed >20-fold increase in the level of latent splicing in Grade III oligodendroglioma compared to normal cells (Figure 5C). Of these three genes, only the function of RELN (reelin) is known. It encodes a large secreted extracellular matrix protein thought to control cell–cell interactions that are critical for cell positioning and neuronal migration during brain development and is involved in a number of neurological disorders. When we compared the level of latent splicing between oligodendroglioma Grade II, oligodendroglioma Grade III and glioblastoma, which is the most aggressive glial malignant tumor, we found that in 23 gene transcripts the level of activation of latent splicing increased in this order.

To identify latent splicing events that are common to breast cancer and gliomas, we compared the latent sites activated in MCF-7 breast cancer cells with the latent splicing activation common to the three glial tumor categories, taking into account only genes expressed in both cell types (249 out of 260 latent sites in MCF-7 cells, Figure 5A; 166 out of 178 latent sites in the glial tumors, Figure 5B). Figure 5D depicts a Venn diagram with an overlap of 16 gene transcripts that show elevation of latent splicing both in breast cancer and glial tumors.

## DISCUSSION

Environmental stress was shown to affect gene expression, either by increasing or decreasing the level of mRNA of defined sets of genes (42). Because exon selection is regulated by many physiological parameters, it is reasonable to assume that the cellular response to stress would also be manifested in changes in the splicing pattern of many mRNAs. Indeed it has been shown that heat shock and other cellular stresses inhibited splicing (2,24,43,44) and also caused changes in the splicing pattern of gene transcripts (2,6,26,31,45).

In this study, we show that stress-induced activation of latent alternative 5′SSs is widespread across the human genome. Using a bioinformatics approach we first revealed the presence of potential latent 5′SSs in most introns (Table 1). The effect of heat shock on latent splicing of specific endogenous genes indicated that the dormant 5′SSs we identified in the bioinformatics search might be amenable for splicing under stress conditions. We thus performed a global analysis of the effect of heat shock on the expression of latent mRNAs and showed that the potential latent 5′SSs we identified in hundreds of genes are indeed activated under these conditions (Figure 3 and Table 2). The genes in which latent splicing was observed after heat shock reflect a wide repertoire of functional groups (Table 3 and Supplementary Table S2), which is in accordance with our bioinformatics analyses. Taken together, these results support a model where the discrimination of latent 5′SSs is an intrinsic feature of the splicing machinery.

The 508 cases of heat-induced activation of latent splicing reported here might very well be an underestimate. This is because in our analysis of human genes, we filtered out genes with uncertain annotation, genes with special splicing signals and genes with PTCs encoding for selenocysteine. We also filtered out alternatively spliced genes and concentrated on those latent 5′SSs positioned <1000 nt from the authentic 5′SS. These limitations, together with the fact that the Affymetrix chip had probesets for only ∼11% of the latent 5′SSs that passed this filtration, resulted in a lower limit of the number of latent sites that are activated by heat shock.

There is at least one more reason why the actual number of heat-activated latent 5′SSs might be higher than that estimated here. Namely, once a latent site is activated for splicing (e.g. by heat shock), the latent mRNA, which now harbors a PTC, becomes subject to the NMD pathway. Indeed, when NMD in the heat shocked cells was abrogated by treatment with CHX, the level of latent splicing was upregulated in both the endogenous genes and the minigenes tested (Figure 4). This experiment highlights an epistatic role for NMD, whereby in cases where latent splicing is activated (e.g. by stress conditions), NMD is recruited to downregulate the potential hazards of a PTC containing mRNA.

The large number of potential latent 5′SSs and our observation that latent splicing can be activated under heat shock and other stress conditions raised the possibility that activation of latent splicing might occur in disease. We therefore analyzed publicly available Human Exon 1.0 ST Array expression data from the GEO, for the activation of latent splicing in disease and under stress conditions, employing the procedure developed for the analysis of latent splicing in our heat-stressed cells. As shown in Table 4, latent splicing was activated under hypoxia stress, as well as in several tumors or tumor cell lines. It should be pointed out that treatment of breast cancer cells with estrogen or camptothecin further activated latent splicing and resulted in generation of a large number of mRNAs harboring PTCs.

Can the occurrence of latent splicing be indicative of a cancerous state? Activation of latent splicing was observed in gene transcripts representing a wide variety of functional groups. It is indicative of a general quality control mechanism for the production of a correct ORF in the mature mRNA, rather than regulation of a specific subset of genes. Yet, in each of the two cancerous conditions we analyzed, breast cancer and glioma, the intersection of three samples revealed highly significant overlaps (260 and 178, respectively). These overlaps underlie a lower limit of a specific group of latent 5′SSs that are activated under each of these conditions (Figure 5). Further comparison of the level of latent splicing in oligodendroglioma Grades II and III, which represent different aggressiveness stages of the same malignant tumor, showed that in 125 gene transcripts the level of relative activation of latent splicing increased with the severity of the disease, reaching higher level of almost two orders of magnitude in TBR1 and SV2B (Figure 5C). It is still premature to assess the contribution of latent splicing to tumor pathogenesis or determine whether the eliciting of latent splicing results from the stressful conditions associated with malignancy. Nevertheless, these results suggest a potential link between activation of sets of latent splice sites and a specific cancerous state. We also used GO and KEGG tools to search whether genes showing activation of latent splicing in stress or cancer cluster in distinct biological pathways, but did not find significant clustering (data not shown). The list of genes in which latent splicing was activated by either heat shock, hypoxia or in cancer is widespread and includes genes with diverse key cellular functions, among them are genes involved in cell proliferation and differentiation. This is not surprising because our bioinformatics search revealed that latent 5′SSs are found in most genes.

Why splicing at latent 5′SSs (latent splicing) appears not to be detected in healthy cells and in cells grown under normal conditions? It should be noted that our bioinformatics analyses revealed that the quality of the latent alternative 5′SSs, as reflected in their score and similarity to the consensus [(2) and Supplementary Figure S3] cannot explain why they are not used for splicing. On the other hand, our previous studies have shown that the removal of all in-frame stop codons between the authentic and a downstream latent site, either by intentional point mutations or by frame-shifting, was sufficient to activate splicing at such sites (3). Also, as shown here, activating splicing from latent sites can be achieved by applying stressful conditions such as heat shock. In principle, two scenarios can account for this phenomenon: (i) Splicing at latent 5′SSs do occur, but the nonsense mRNAs thus formed are rapidly and efficiently degraded, by any RNA surveillance mechanism (e.g. NMD) to a level below detection and (ii) splicing events at intronic latent 5′SSs that are preceded by at least one stop codon in frame with the upstream exon are suppressed. We tested the first scenario in a substantial number of independent experiments [summarized in Supplementary Table S1 of (34)], but the data do not fit a model that could attribute the lack of latent splicing to a rapid and complete degradation of latent mRNAs by NMD or by its hUpf1-dependent branches or even by a yet unknown RNA surveillance mechanism. On the other hand, these data fit the second scenario and invoke a mechanism—termed SOS—proposed to suppress splicing involving latent alternative 5′SSs whose usage could introduce an in-frame stop codon into the resultant mRNA (34).

The concept of SOS is difficult to conceive, mainly because the occurrence of such a mechanism implies that open reading frames of mRNAs can be recognized in the nucleus—prior to splicing. Namely, this concept is not in line with the well-established paradigm asserting that reading frames are recognized only in fully spliced mRNAs by ribosomes residing in the cell cytoplasm. Further studies are required to verify the SOS mechanism and determine how it works. Nevertheless, supporting evidence for the idea that PTC-harboring pre-mRNAs can be recognized in the cell nucleus, resulting in suppressed splicing, has been provided in a number of studies (46–48), including a recent study showing that un-spliced PTC-harboring transcripts are retained at nuclear transcription sites (49).

Since we have shown that the lack of latent splicing under normal growth conditions is due to SOS from these sites, we propose that the widespread activation of latent splicing under stress is due to abrogation of the SOS mechanism. Such abrogation might be caused by conformational changes in SOS components or due to disruption of interactions between components of the SOS machinery and the pre-mRNA, which might occur under stress. Nevertheless, independent of the mechanism underlying the silencing of latent splicing under normal growth conditions and its abrogation under stress, the fact that the activation of latent splice sites under stress and in cancer is widespread is likely to have medical implications.

We found that activation of latent splicing occurred in thousands of genes under stress and in cancers (Tables 3 and 4; Supplementary Tables S2, S3 and S4). It is possible that different types of stresses might activate latent splicing in different sets of genes and to a different extent. For example, we have previously shown that treatment with pactamycin activated latent splicing (31) and global analysis of this effect revealed that the list of genes activated by heat shock differed from that activated by pactamycin. Yet, there was an overlap between the two lists (data not shown). Similarly, different sets of latent 5′SSs were activated in the different cancers tested, and yet there was an overlap between them. This kind of behavior can be attributed to transcript-specific elements (e.g. splicing enhancers and silencers) which, in concert with spliceosomal components, control the pattern of splicing of specific introns. Interestingly, however, the integrated number of latent 5′SSs that were activated by the different stresses and in the analyzed cancers covers the majority of cases identified by the bioinformatics survey (∼58%). It is thus possible that most bioinformatics-identified latent 5′SSs might be activated in a variety of stressful conditions yielding PTC harboring mRNAs.

Another question arising from our initial bioinformatics analyses pertains the unexpected high abundance, within introns, of legitimate alternative 5′SS sequences that are not used for splicing and remain silent. A plausible model to explain this phenomenon integrates two findings: one showing that U1 snRNP, in addition to its role in RNA splicing, plays a role in protecting pre-mRNA transcripts from premature cleavage and polyadenylation (PCPA) (50,51) and the other, our finding that most intronic latent 5′SSs are preceded by a stop codon in frame with the upstream authentic exon. The way U1 snRNP acts to prevent PCPA suggests its binding to putative 5′SSs (50,51). Such binding, however, could have promoted splicing events leading to undesirable or even harmful mRNAs, which indeed occurred under stress and in cancer. It can thus be speculated that under normal growth conditions, the occurrences of in-frame stop codons upstream of the binding sites for U1 snRNPs help suppress splicing from the latent 5′SSs via a quality control mechanism (34) and, at the same time, allow for other interactions that prevent PCPA. It should be pointed out that the numerous latent 5′SSs also provide an option for generation of new splice isoforms via activation of latent splicing.

In summary, global analyses of changes in latent splicing in stress and disease are presented here for the first time. Our analyses revealed significant changes in alternative 5′SS selection in thousands of genes as a result of stress and disease. The eliciting of latent splicing observed in cancer cells might be involved in tumor pathogenesis. It can also be speculated that the expression of latent sequences may lead to the expression of new antigens in stressed tissues, which can be recognized by the host immune system as foreign and danger signals. The phenomenon described here may therefore be relevant to autoimmune disorders on the one hand and to cancer immunotherapy on the other.

## SUPPLEMENTARY DATA

Supplementary Data are available at NAR Online: Supplementary Tables 1–6, Supplementary Figures 1–5 and Supplementary Text.

## FUNDING

Yeda CEO Fund (to J.S.); Israel Ministry of Health (to J.S.); Israel Cancer Research Fund (to J.S. and R.S.); Helen and Milton Kimmelman Center for Biomolecular Structure and Assembly at the Weizmann Institute of Science (to J.S.). Funding for open access charge: Waived by Oxford University Press.

*Conﬂict of interest statement.* None declared.

## Supplementary Material

Supplementary Data

## References

[1] Wang Z, Burge CB (2008). Splicing regulation: from a parts list of regulatory elements to an integrated splicing code. RNA.

[2] Miriami E, Motro U, Sperling J, Sperling R (2002). Conservation of an open-reading frame as an element affecting 5′ splice site selection. J. Struct. Biol..

[3] Li B, Wachtel C, Miriami E, Yahalom G, Friedlander G, Sharon G, Sperling R, Sperling J (2002). Stop codons affect 5′ splice site selection by surveillance of splicing. Proc. Natl Acad. Sci. USA.

[4] Miriami E, Sperling R, Sperling J, Motro U (2004). Regulation of splicing: the importance of being translatable. RNA.

[5] Sperling J, Sperling R (2008). Nuclear surveillance of RNA polymerase II transcripts. RNA Biol..

[6] Stamm S (2002). Signals and their transduction pathways regulating alternative splicing: a new dimension of the human genome. Hum. Mol. Genet..

[7] Johnson JM, Castle J, Garrett-Engele P, Kan Z, Loerch PM, Armour CD, Santos R, Schadt EE, Stoughton R, Shoemaker DD (2003). Genome-wide survey of human alternative pre-mRNA splicing with exon junction microarrays. Science.

[8] Stamm S, Ben-Ari S, Rafalska I, Tang Y, Zhang Z, Toiber D, Thanaraj TA, Soreq H (2005). Function of alternative splicing. Gene.

[9] Clark TA, Schweitzer AC, Chen TX, Staples MK, Lu G, Wang H, Williams A, Blume JE (2007). Discovery of tissue-specific exons using comprehensive human exon microarrays. Genome Biol..

[10] Pan Q, Shai O, Lee LJ, Frey BJ, Blencowe BJ (2008). Deep surveying of alternative splicing complexity in the human transcriptome by high-throughput sequencing. Nat. Genet..

[11] Ben-Dov C, Hartmann B, Lundgren J, Valcarcel J (2008). Genome-wide analysis of alternative Pre-mRNA splicing. J. Biol. Chem..

[12] Cáceres JF, Kornblihtt AR (2002). Alternative splicing: multiple control mechanisms and involvement in human disease. Trends Genet..

[13] Srebrow A, Kornblihtt AR (2006). The connection between splicing and cancer. J. Cell Sci..

[14] Pajares MJ, Ezponda T, Catena R, Calvo A, Pio R, Montuenga LM (2007). Alternative splicing: an emerging topic in molecular and clinical oncology. Lancet Oncol..

[15] Skotheim RI, Nees M (2007). Alternative splicing in cancer: noise, functional, or systematic?. Int. J. Biochem. Cell Biol..

[16] Karni R, de Stanchina E, Lowe SW, Sinha R, Mu D, Krainer AR (2007). The gene encoding the splicing factor SF2/ASF is a proto-oncogene. Nat. Struct. Mol. Biol..

[17] Miriami E, Sperling J, Sperling R (1994). Heat shock affects 5′ splice site selection, cleavage and ligation of CAD pre-mRNA in hamster cells, but not its packaging in lnRNP particles. Nucleic Acids Res..

[18] Wachtel C, Li B, Sperling J, Sperling R (2004). Stop codon-mediated suppression of splicing is a novel nuclear scanning mechanism not affected by elements of protein synthesis and NMD. RNA.

[19] Kultz D (2005). Molecular and evolutionary basis of the cellular stress response. Annu. Rev. Physiol..

[20] Black DL (2003). Mechanisms of alternative pre-messenger RNA splicing. Annu. Rev. Biochem..

[21] Sperling J, Azubel M, Sperling R (2008). Structure and function of the pre-mRNA splicing machine. Structure.

[22] Wahl MC, Will CL, Lührmann R (2009). The spliceosome: design principles of a dynamic RNP machine. Cell.

[23] Rino J, Carmo-Fonseca M (2009). The spliceosome: a self-organized macromolecular machine in the nucleus?. Trends Cell Biol..

[24] Yost HJ, Lindquist S (1986). RNA splicing is interrupted by heat shock and is rescued by heat shock protein synthesis. Cell.

[25] Yost HJ, Lindquist S (1991). Heat-shock proteins affect RNA processing during the heat-shock response of saccharomyces-cerevisiae. Mol. Cell. Biol..

[26] Biamonti G, Caceres JF (2009). Cellular stress and RNA splicing. Trends Biochem. Sci..

[27] Busà R, Sette C (2010). An emerging role for nuclear RNA-mediated responses to genotoxic stress. RNA Biol..

[28] Pick M, Perry C, Lapidot T, Guimaraes-Sternberg C, Naparstek E, Deutsch V, Soreq H (2006). Stress-induced cholinergic signaling promotes inflammation-associated thrombopoiesis. Blood.

[29] Munoz MJ, Perez Santangelo MS, Paronetto MP, de la Mata M, Pelisch F, Boireau S, Glover-Cutter K, Ben-Dov C, Blaustein M, Lozano JJ (2009). DNA damage regulates alternative splicing through inhibition of RNA polymerase II elongation. Cell.

[30] Kingston RE, Ausubel FM, Brent R, Kingston RE, Moore DD, Seidman JG, Smith JA, Struhl K (1992). Short Protocols in Molecular Biology.

[31] Kamhi E, Yahalom G, Kass G, Hacham Y, Sperling R, Sperling J (2006). AUG sequences are required to sustain nonsense-codon-mediated suppression of splicing. Nucleic Acids Res..

[32] Shapiro MB, Senapathy P (1987). RNA splice junctions of different classes of eukaryotes: sequence statistics and functional implications in gene expression. Nucleic Acids Res..

[33] Schwartz S, Ast G (2010). Chromatin density and splicing destiny: on the cross-talk between chromatin structure and splicing. EMBO J..

[34] Kamhi E, Raitskin O, Sperling R, Sperling J (2010). A potential role for initiator-tRNA in pre-mRNA splicing regulation. Proc. Natl Acad. Sci. USA.

[35] Rajavel KS, Neufeld EF (2001). Nonsense-mediated decay of human HEXA mRNA. Mol. Cell. Biol..

[36] Maquat LE, Li X (2001). Mammalian heat shock p70 and histone H4 transcripts, which derive from naturally intronless genes, are immune to nonsense-mediated decay. RNA.

[37] Hang X, Li P, Li Z, Qu W, Yu Y, Li H, Shen Z, Zheng H, Gao Y, Wu Y (2009). Transcription and splicing regulation in human umbilical vein endothelial cells under hypoxic stress conditions by exon array. BMC Genomics.

[38] Bitton DA, Okoniewski MJ, Connolly Y, Miller CJ (2008). Exon level integration of proteomics and microarray data. BMC Bioinformatics.

[39] Dutertre M, Gratadou L, Dardenne E, Germann S, Samaan S, Lidereau R, Driouch K, de la Grange P, Auboeuf D (2010). Estrogen regulation and physiopathologic significance of alternative promoters in breast cancer. Cancer Res..

[40] Dutertre M, Sanchez G, De Cian MC, Barbier J, Dardenne E, Gratadou L, Dujardin G, Le Jossic-Corcos C, Corcos L, Auboeuf D (2010). Cotranscriptional exon skipping in the genotoxic stress response. Nat. Struct.Mol. Biol..

[41] French PJ, Peeters J, Horsman S, Duijm E, Siccama I, van den Bent MJ, Luider TM, Kros JM, van der Spek P (2007). Identification of differentially regulated splice variants and novel exons in glial brain tumors using exon expression arrays. Cancer Res..

[42] Pleiss JA, Whitworth GB, Bergkessel M, Guthrie C (2007). Transcript specificity in yeast pre-mRNA splicing revealed by mutations in core spliceosomal components. PLoS Biol..

[43] Bond U (2006). Stressed out! Effects of environmental stress on mRNA metabolism. FEMS Yeast Res..

[44] Jolly C, Lakhotia SC (2006). Human sat III and Drosophila hsr omega transcripts: a common paradigm for regulation of nuclear RNA processing in stressed cells. Nucleic Acids Res..

[45] Kaufer D, Friedman A, Seidman S, Soreq H (1998). Acute stress facilitates long-lasting changes in cholinergic gene expression. Nature.

[46] Mühlemann O, Mock-Casagrande CS, Wang J, Li S, Custodio N, Carmo-Fonseca M, Wilkinson MF, Moore MJ (2001). Precursor RNAs harboring nonsense codons accumulate near the site of transcription. Mol. Cell.

[47] Aoufouchi S, Yelamos J, Milstein C (1996). Nonsense mutations inhibit RNA splicing in a cell-free system: recognition of mutant codon is independent of protein synthesis. Cell.

[48] Gersappe A, Burger L, Pintel DJ (1999). A premature termination codon in either exon of minute virus of mice P4 promoter-generated pre-mRNA can inhibit nuclear splicing of the intervening intron in an open reading frame-dependent manner. J. Biol. Chem..

[49] de Turris V, Nicholson P, Orozco RZ, Singer RH, Mühlemann O (2011). Cotranscriptional effect of a premature termination codon revealed by live-cell imaging. RNA.

[50] Kaida D, Berg MG, Younis I, Kasim M, Singh LN, Wan L, Dreyfuss G (2010). U1 snRNP protects pre-mRNAs from premature cleavage and polyadenylation. Nature.

[51] Berg MG, Singh LN, Younis I, Liu Q, Pinto AM, Kaida D, Zhang Z, Cho S, Sherrill-Mix S, Wan L (2012). U1 snRNP determines mRNA length and regulates isoform expression. Cell.

