# Supplementary material for: Genome-wide activation of latent donor splice sites in stress and disease
Source: Nucleic Acids Res. 2012 Sep 23;40(21):10980–94. doi: 10.1093/nar/gks834 (PMC3510495; doi:10.1093/nar/gks834)
Supplement: Supplementary Data [file supp_40_21_10980__index.html]

Genome-wide activation of latent donor splice sites in stress and disease — Genome-wide activation of latent donor splice sites in stress and disease — Supplementary Data 

# Genome-wide activation of latent donor splice sites in stress and disease

## Supplementary Data

files

**Files in this Data Supplement:**

- Supplementary Data - pdf file
- Supplementary Data - pdf file
