# Supplementary material for: Genome-wide activation of latent donor splice sites in stress and disease
Source: Nucleic Acids Res. 2012 Sep 23;40(21):10980–94. doi: 10.1093/nar/gks834 (PMC3510495; doi:10.1093/nar/gks834)
Supplement: Supplementary Data [file supp_gks834_nar-01533-a-2012-File008.pdf]

Genome-wide activation of latent donor splice sites in stress and disease

Yuval Nevo[1*], Eyal Kamhi[2*], Jasmine Jacob-Hirsch[3], Ninette Amariglio[3], Gideon Rechavi[3],

Joseph Sperling[2] and Ruth Sperling[1§]

Supplementary Text & Figures

Do stress-activated latent 5'SSs differ from those that were not activated?

Activation of thousands of latent 5'SSs in stress and cancer enabled us to search for characteristics of latent 5'SSs and especially to compare those latent sites that were activated in stress and cancer to those that were not. First, we compared the length distribution of the latent exons that were activated in stress and cancer to those that were not, and we did not find a significant difference (Supplementary Figure S3). Next, we analyzed the length distribution of the extended latent exons (the latent exon combined with its upstream authentic exon), which represent the new exons resulting from activation of latent splicing. When we compared the length distribution of authentic exons with that of extended latent exons (from all the analyses of stress and disease), we can see that only ~8% of the authentic exons are above 1,000 nt long (Supplementary Figure S4). This justifies the cut-off of 1,000 nt for the length of latent exons analyzed in this study. The finding that ~8% of the activated latent exons (Supplementary Figure S3) are between 901-1,000 nt is in further support of this cut-off. Comparing the length distribution of the extended latent exons that were activated to those that were not we find a very significant difference between the two groups. The main contribution comes from the larger fraction of non-activated extended latent exons of length above 1,000 nt, yet ~15% of the activated extended latent exons are longer than 1,000 nt. We can therefore conclude that, up to 1,000 nt, the length of the latent exon is not an essential factor in determining if a latent site will be activated or not.

Our previous 5'SS sequence analysis had shown that the consensus for the latent 5'SSs was not significantly different from that of the authentic ones (4). Because this analysis used a relatively small data set and a different criterion for the assignment of latent 5'SSs, we repeated here this analysis using a much larger database and a more stringent criterion (a cut-off of ~87.2

for the Shapiro and Senapathy (S&S) splice site score (5) of latent 5'SSs). Furthermore, in the present study we analyzed the score distribution of latent sites that were activated in stress and cancer and compared it with the score distribution of latent sites that were not activated. In supplementary Figure S5A we compare the S&S score distribution for all authentic 5'SSs, all latent 5'SSs and those latent sites that were either activated or not in stress and disease. As we selected a cut-off of ~87.2 for the score of latent 5'SSs, the majority of latent 5'SSs (~80%), weather activated or not, range between 87-92, and there is no significant difference between the score distribution of latent 5'SSs that were activated in stress and cancer and those that were not activated (Supplementary Figure S5A). Thus, a more stringent cut-off would not have discriminated activated (confirmed) latent 5'SSs from non-activated ones. Also, when we compared the sequence pattern, represented as a pictogram of the weight matrices of all the latent sites activated in stress and cancer with that of non-activated ones (Supplementary Figure S5B), no significant differences were detected (χ^2 test with the Bonferroni correction).

However, when we take into consideration each latent site with its upstream authentic counterpart, doing this by comparing the ratio of the score of each latent 5'SS to the score of its upstream authentic 5'SS and plotting the distribution of these ratios for the activated and non-activated latent 5'SSs (Supplementary Figure S5C) we find that the activated latent 5'SSs tend to have a higher score than their upstream authentic 5'SS (P-value<0.014). Similar results were obtained when we compared the difference between the score of each latent 5'SS to the score of its upstream authentic 5'SS, and plotting the distribution of these differences for the activated and non-activated latent 5'SSs (data not shown). Nonetheless, having a better score than its upstream authentic 5'SS counterpart is not an essential condition for activation of a given latent 5'SS, since ~40% of the activated latent 5'SSs have a lower score than their upstream authentic 5'SS, and yet they are activated (Supplementary Figure S5C).

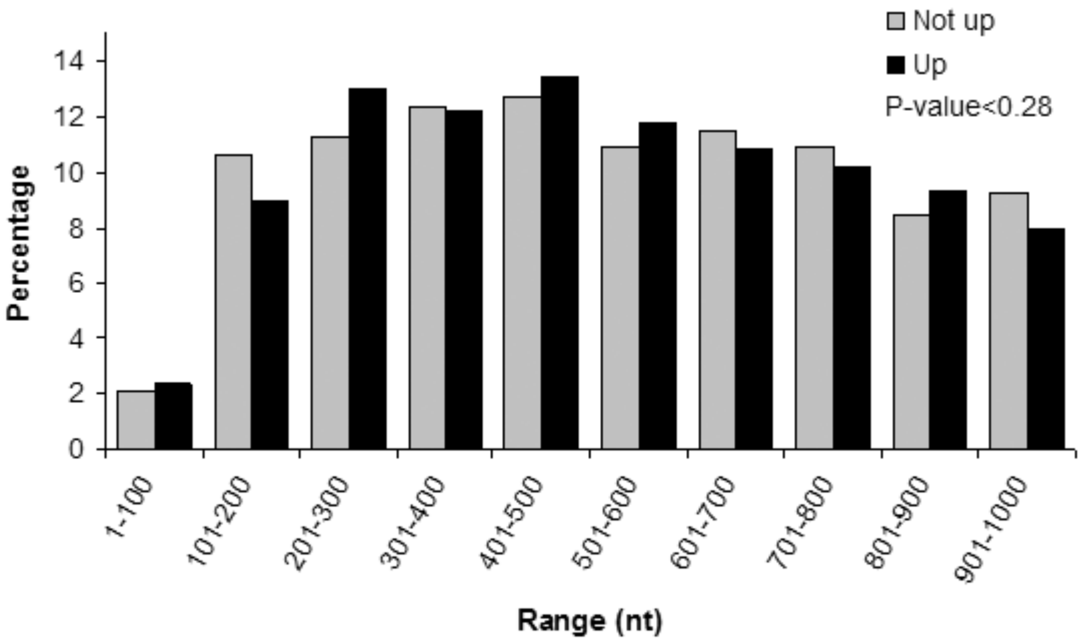

Supplementary Figure S1. Length distribution of latent exons. Lengths up to 1,000 nt were divided into bins of 100 nt. The percentage of latent exons in each such bin is displayed. Grey bars, latent exons that were not activated in any of the analyzed microarray data; black bars, latent exons that were activated in at least one of the analyzed microarray data. The length distribution of activated latent exons was compared to that of non-activated latent exons and the P-value of χ^2 test is displayed.

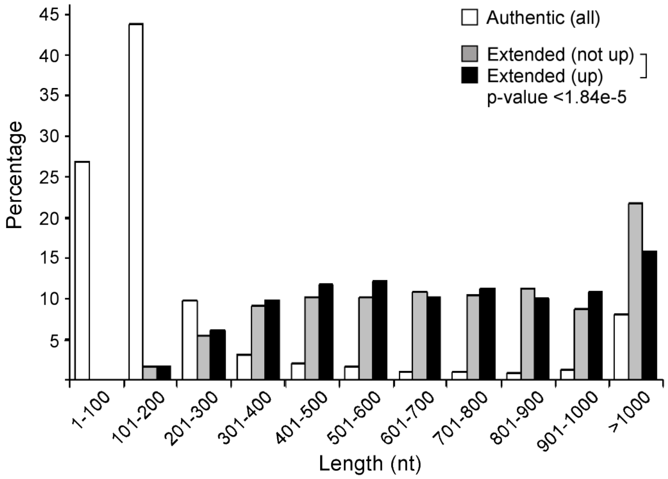

Supplementary Figure S2. Length distribution of authentic and extended exons. Lengths up to 1,000 nt were divided into bins of 100 nt, while all lengths above 1,000 nt were binned together. The percentage of exons in each such bin is displayed. **Authentic**, all exons in the database; **Extended,** the latent exon combined with its upstream authentic exon. Extended (not up), extended latent exons that were not activated in any of the analyzed microarray data sets; Extended (up), extended latent exons that were activated (at least 1.5 fold) in at least one of the analyzed microarray data sets. The length distribution of activated extended latent exons was compared to that of non-activated extended latent exons and the P-value of χ^2 test is displayed.

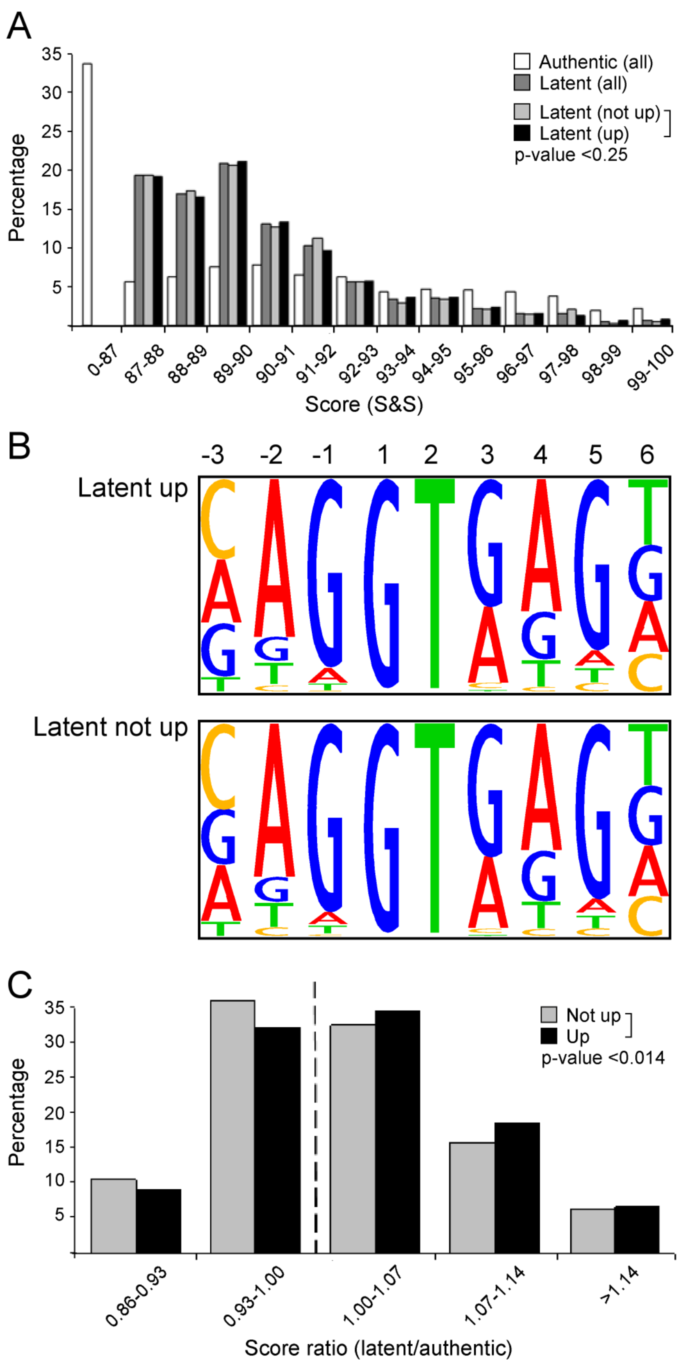

Supplementary Figure S3. Patterns and scores of latent 5' splice sites. Scores of 5'SSs were calculated according to Shapiro and Senapathy (5). (**A**) Distribution of scores. All scores between 0 and 87 (87.2 is approximately the threshold used for searching latent 5'SSs) are displayed as a single bin, followed by bins of 1. Authentic (all), all authentic 5'SSs in the database; Latent (all), all latent 5'SSs analyzed in at least one of the microarray data sets; Latent (not up), latent 5'SSs that were not activated in any of the analyzed microarray data sets; Latent (up), latent 5'SSs that were activated in at least one of the analyzed microarray data sets. The score distribution of activated latent 5'SSs was compared to that of

non-activated latent 5'SSs and the P-value of χ^2 test is displayed. (**B**) The 5’SS sequence patterns, represented as weight matrices, of latent sites activated in stress and cancer is compared with that of non-activated latent sites. The height of each letter is proportional to the frequency of the corresponding base at the given position, and bases are listed in descending order of frequency from top to bottom. **(C)** Distribution of score ratios between latent 5'SSs and their upstream authentic counterparts. Score ratios were binned as displayed on the X-axis, the vertical dashed line separates between latent 5'SSs with a lower score than the one of their upstream authentic 5'SS (on its left) and latent 5'SSs with a higher score than the one of their upstream authentic 5'SS (to its right). Not up, latent 5'SSs that were not activated in any of the analyzed microarray data; Up, latent 5'SSs that were activated in at least one of the analyzed microarray data. The score ratio distribution of activated latent 5'SSs was compared to that of non-activated latent 5'SSs and the P-value of χ^2 test is displayed.

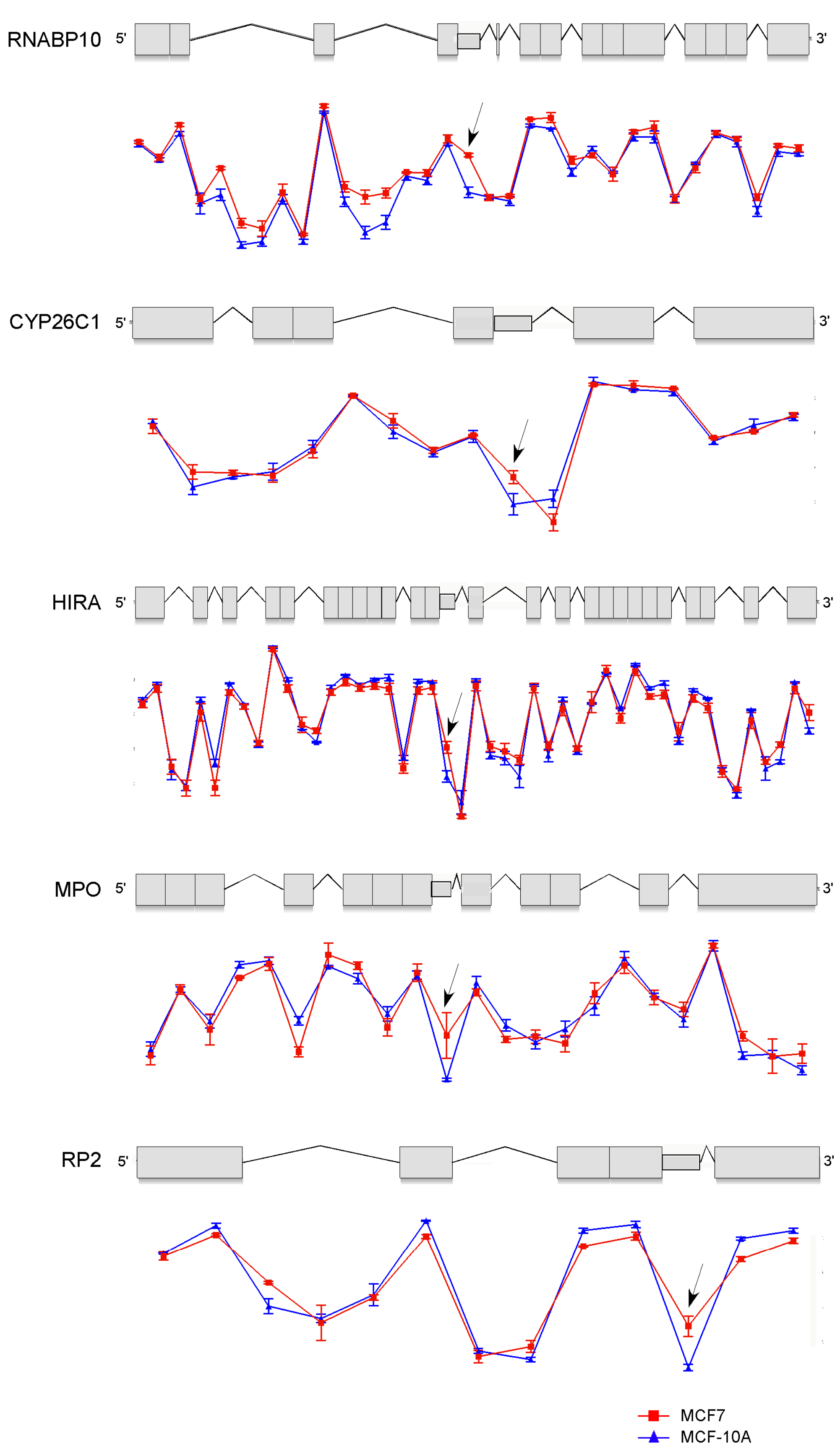
RNABP10
5'
3'
CYP26C1
5'
3'
HIRA
5'
3'
MPO
5'
3'
RP2
5'
3'
MCF7
MCF-10A

Supplementary Figure S4. Microarray-detected elevation in the level of latent splicing in MCF-7 breast cancer cells compared to MCF-10A cells. Curves corresponding to the levels of probesets signals along the genes were created using the Partek Genomics Suite software (red, MCF-7 cells; blue, MCF-10A cells). Arrows point at probesets that recognized latent exons. Schematics of the genes are drawn above the expression curves. Boxes, exons; lines, introns; narrow boxes, latent exons. **RANBP10**, RAN binding protein 10, MCF-7 (from ref. 1); **CYP26C1**, cytochrome P450, subfamily C, polypeptide A, MCF-7 (from ref. 2); **HIRA**, HIR histone cell cycle regulation defective homolog A, MCF-7 (from ref. 3); **MPO**, myeloperoxidase, MCF-7 (from ref. 1); **RP2**, retinitis pigmentosa 2 (X-linked recessive), MCF-7 (from ref. 2).

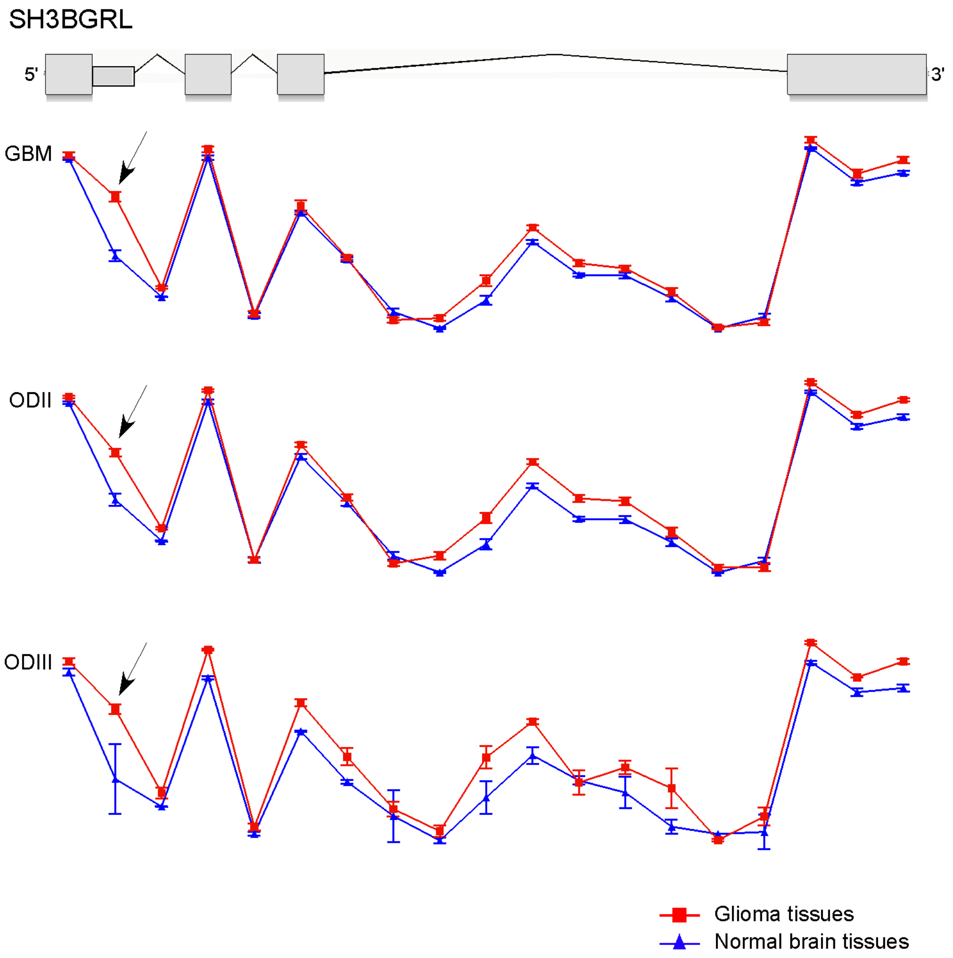

Supplementary Figure S5. An example of a latent splice site that was activated in all three gliomas. Microarray-detected elevation in the level of latent splicing in the first intron of the gene **SH3BGRL**, SH3 domain binding glutamic acid-rich protein like, was detected in glioblastoma (GBM), oligodendroglioma II (ODII), and oligodendroglioma III (ODIII). Curves corresponding to the levels of probesets signals along the gene were created using the Partek Genomics Suite software (red, glioma; blue, normal brain). Arrows point at the probeset that recognized the latent exon. Schematics of the genes are drawn above the expression curves. Boxes, exons; lines, introns; narrow box, latent exon.

References (for Supplementary Text & Figures)

1. Dutertre, M., Sanchez, G., De Cian, M.C., Barbier, J., Dardenne, E., Gratadou, L., Dujardin, G., Le Jossic-Corcos, C., Corcos, L. and Auboeuf, D. (2010) Cotranscriptional exon skipping in the genotoxic stress response. *Nat. Struct. Mol. Biol.*, **17**, 1358-U1218.
2. Bitton, D.A., Okoniewski, M.J., Connolly, Y. and Miller, C.J. (2008) Exon level integration of proteomics and microarray data. *BMC Bioinformatics*, **9**, 118.
3. Dutertre, M., Gratadou, L., Dardenne, E., Germann, S., Samaan, S., Lidereau, R., Driouch, K., de la Grange, P. and Auboeuf, D. (2010) Estrogen Regulation and

Physiopathologic Significance of Alternative Promoters in Breast Cancer. *Cancer Res.*, **70**, 3760-3770.

4. Miriami, E., Motro, U., Sperling, J. and Sperling, R. (2002) Conservation of an open-reading frame as an element affecting 5' splice site selection. *J. Struct. Biol.*, **140**, 116-122.

5. Shapiro, M.B. and Senapathy, P. (1987) RNA splice junctions of different classes of eukaryotes: sequence statistics and functional implications in gene expression. *Nucleic Acids Res.*, **15**, 7155-7174.
