# Supplementary material for: Genome-wide activation of latent donor splice sites in stress and disease
Source: Nucleic Acids Res. 2012 Sep 23;40(21):10980–94. doi: 10.1093/nar/gks834 (PMC3510495; doi:10.1093/nar/gks834)
Supplement: Supplementary Data [file supp_gks834_nar-01533-a-2012-File009.pdf]

Genome-wide activation of latent donor splice sites in stress and disease

Yuval Nevo[1*], Eyal Kamhi[2*], Jasmine Jacob-Hirsch[3], Ninette Amariglio[3], Gideon Rechavi[3],

Joseph Sperling[2] and Ruth Sperling[1§]

Supplementary Material

Supplementary Tables

Supplementary Table 1. 5' splice sites scoring matrix (PSSM)

Position[a]	-3	-2	-1	+1	+2	+3	+4	+5	+6
A	33.57	63.11	9.70	0.03	0.02	60.45	71.06	8.36	17.47
C	36.05	11.01	2.83	0.02	0.78	2.45	7.23	5.52	15.00
G	18.08	11.60	80.72	99.95	0.03	34.52	11.10	78.89	18.72
T	12.30	14.28	6.75	0.00	99.17	2.58	10.61	7.23	48.81

[a]Relative to the 5' splice site

Supplementary Table 2. List of latent 5' splice sites (SSs) activated by heat shock

Gene symbol	Gene name	Location of latent 5'SS	Intron number	Position of latent 5'SS[a]	Number of stop codons[b]	Latent score[c]	Authentic score[c]	Log score ratio[d]	Fold change
SLC10A1	solute carrier family 10 (sodium/bile acid cotransporter family), member 1	chr14:69332857	1	412	9	90.22	92.98	-0.04	32.46
COL5A2	collagen, type V, alpha 2	chr2:189658227	10	462	10	91.41	86.71	0.08	21.77
IL12RB2	interleukin 12 receptor, beta 2	chr1:67560431	3	272	4	94.41	89.96	0.07	5.70
MYH7B	myosin, heavy chain 7B, cardiac muscle, beta	chr20:33033080	7	557	8	92.23	89.31	0.05	5.31
COL5A2	collagen, type V, alpha 2	chr2:189625508	40	211	5	90.42	87.29	0.05	5.11
PRKG2	protein kinase, cGMP-dependent, type II	chr4:82249771	14	854	20	96.36	86.98	0.15	5.02
TMED10	transmembrane emp24-like trafficking protein 10 (yeast)	chr14:74672120	4	95	1	93.27	91.96	0.02	4.95
CALR	calreticulin	chr19:12912956	7	256	1	89.93	96.36	-0.10	4.73
CUL7	cullin 7	chr6:43127523	2	401	9	90.99	92.66	-0.03	4.70
HHLA1	HERV-H LTR-associating 1	chr8:133152640	5	128	2	97.10	90.05	0.11	4.54
FOLR2	folate receptor 2 (fetal)	chr11:71607578	2	153	1	88.78	85.28	0.06	4.53
SORCS3	sortilin-related VPS10 domain containing receptor 3	chr10:106898316	9	773	19	92.07	91.89	0.00	4.48
SORL1	sortilin-related receptor, L(DLR class) A repeats containing	chr11:120927512	16	924	22	91.41	97.23	-0.09	4.28
DNLZ	DNL-type zinc finger	chr9:138377496	1	263	1	91.99	91.84	0.00	4.01
FTSJD2	FtsJ methyltransferase domain containing 2	chr6:37512133	2	618	8	91.41	93.00	-0.02	4.00
SERHL2	serine hydrolase-like 2	chr22:41299154	11	676	5	88.63	95.05	-0.10	3.87
ZNF530	zinc finger protein 530	chr19:62803956	1	643	7	94.63	96.83	-0.03	3.82
GRIK4	glutamate receptor, ionotropic, kainate 4	chr11:120213490	6	728	13	90.61	92.54	-0.03	3.69
PNPO	pyridoxamine 5'-phosphate oxidase	chr17:43374413	1	236	3	88.59	81.54	0.12	3.69
TECTA	tectorin alpha	chr11:120504892	8	630	13	94.63	97.10	-0.04	3.67
PYGB	phosphorylase, glycogen; brain	chr20:25206331	7	370	1	88.65	88.18	0.01	3.67
JAM3	junctional adhesion molecule 3	chr11:133516204	3	330	5	94.73	85.93	0.14	3.62
C9orf102	chromosome 9 open reading frame 102	chr9:97731255	11	289	14	91.66	87.65	0.06	3.60
DAZAP2	DAZ associated protein 2	chr12:49921332	3	166	2	92.04	86.42	0.09	3.57
MTR	5-methyltetrahydrofolate-homocysteine methyltransferase	chr1:235059408	12	218	6	90.96	94.43	-0.05	3.56
FGD1	FYVE, RhoGEF and PH domain containing 1	chrX:54490201	17	267	3	88.59	91.77	-0.05	3.51
PLCG2	phospholipase C, gamma 2 (phosphatidylinositol-specific)	chr16:80472990	8	932	16	88.65	90.94	-0.04	3.50
VGLL1	vestigial like 1 (Drosophila)	chrX:135461456	4	811	12	88.72	87.06	0.03	3.46
KLHL22	kelch-like 22 (Drosophila)	chr22:19155350	3	286	3	92.98	97.36	-0.07	3.39

Gene symbol	Gene name	Location of latent 5’SS	Intron number	Position of latent 5’SS[a]	Number of stop codons[b]	Latent score[c]	Authentic score[c]	Log score ratio[d]	Fold change
SLC25A12	solute carrier family 25 (mitochondrial carrier, Aralar), member 12	chr2:172355708	15	498	14	89.48	92.23	-0.04	3.38
DEPDC4	DEP domain containing 4	chr12:99179640	3	532	8	97.36	90.69	0.10	3.34
B4GALT5	UDP-Gal:betaGlcNAc beta 1,4- galactosyltransferase, polypeptide 5	chr20:47762522	1	997	7	96.36	88.11	0.13	3.33
CLN6	ceroid-lipofuscinosis, neuronal 6, late infantile, variant	chr15:66297302	2	625	9	90.88	93.30	-0.04	3.31
CENPQ	centromere protein Q	chr6:49546382	2	433	6	90.91	88.92	0.03	3.29
TTC9B	tetratricopeptide repeat domain 9B	chr19:45414210	2	723	5	88.72	99.13	-0.16	3.27
RAB36	RAB36, member RAS oncogene family	chr22:21828634	6	416	1	92.64	91.46	0.02	3.17
ATP8B1	ATPase, aminophospholipid transporter, class I, type 8B, member 1	chr18:53506173	12	355	5	91.43	94.17	-0.04	3.15
PADI4	peptidyl arginine deiminase, type IV	chr1:17535721	4	414	1	92.64	83.59	0.15	3.12
JAK2	Janus kinase 2	chr9:5061046	12	995	17	92.66	82.46	0.17	3.12
CLMN	calmin (calponin-like, transmembrane)	chr14:94750767	5	963	16	89.96	97.23	-0.11	3.11
LRP5	low density lipoprotein receptor-related protein 5	chr11:67914345	7	250	3	89.46	95.18	-0.09	3.02
C1R	complement component 1, r subcomponent	chr12:7132902	5	127	1	91.36	84.91	0.11	2.97
ZFPL1	zinc finger protein-like 1	chr11:64609649	3	376	8	89.86	91.53	-0.03	2.94
ZNF287	zinc finger protein 287	chr17:16411219	2	148	2	89.21	95.23	-0.09	2.92
ESYT3	extended synaptotagmin-like protein 3	chr3:139669279	11	139	1	88.72	82.73	0.10	2.90
DIP2C	DIP2 disco-interacting protein 2 homolog C (Drosophila)	chr10:393447	23	320	2	88.70	91.37	-0.04	2.90
PDGFRB	platelet-derived growth factor receptor, beta polypeptide	chr5:149480338	18	305	1	88.59	83.94	0.08	2.87
RAB25	RAB25, member RAS oncogene family	chr1:154303074	2	554	5	88.96	80.59	0.14	2.82
PHF23	PHD finger protein 23	chr17:7082830	1	458	5	89.93	95.69	-0.09	2.82
C2orf52	chromosome 2 open reading frame 52	chr2:232085543	2	938	17	93.27	89.65	0.06	2.80
MAP9	microtubule-associated protein 9	chr4:156508421	5	766	13	95.18	85.87	0.15	2.80
ST7OT4	ST7 overlapping transcript 4 (non-protein coding)	chr7:116384278	4	266	13	88.63	86.71	0.03	2.80
PYROXD2	pyridine nucleotide-disulphide oxidoreductase domain 2	chr10:100136458	14	489	6	91.43	87.65	0.06	2.79
PTPN9	protein tyrosine phosphatase, non-receptor type 9	chr15:73601919	4	597	12	90.46	93.30	-0.04	2.76
HTT	huntingtin	chr4:3106731	20	667	19	92.83	100.00	-0.11	2.76
CNIH4	cornichon homolog 4 (Drosophila)	chr1:222611390	1	73	1	93.20	65.08	0.52	2.75

Gene symbol	Gene name	Location of latent 5'SS	Intron number	Position of latent 5'SS[a]	Number of stop codons[b]	Latent score[c]	Authentic score[c]	Log score ratio[d]	Fold change
SPATA21	spermatogenesis associated 21	chr1:16599349	11	452	9	89.93	91.53	-0.03	2.75
PLIN2	perilipin 2	chr9:19115983	3	128	2	89.06	90.91	-0.03	2.73
NR1D1	nuclear receptor subfamily 1, group D, member 1	chr17:35503408	7	282	4	90.14	95.28	-0.08	2.73
TTC25	tetratricopeptide repeat domain 25	chr17:37345319	2	193	2	90.68	90.22	0.01	2.73
FAR1	fatty acyl CoA reductase 1	chr11:13689097	5	105	4	90.54	88.65	0.03	2.70
MRPL18	mitochondrial ribosomal protein L18	chr6:160132366	2	219	4	95.23	91.83	0.05	2.68
LPCAT4	lysophosphatidylcholine acyltransferase 4	chr15:32443994	3	506	5	90.39	94.18	-0.06	2.68
HCLS1	hematopoietic cell-specific Lyn substrate 1	chr3:122858740	3	75	1	96.22	88.19	0.13	2.68
M6PR	mannose-6-phosphate receptor (cation dependent)	chr12:8987217	5	50	1	90.27	89.31	0.02	2.68
PRDM4	PR domain containing 4	chr12:106658312	10	540	10	88.65	100.00	-0.17	2.67
ZNF287	zinc finger protein 287	chr17:16409809	3	754	18	88.65	90.91	-0.04	2.64
ROCK1	Rho-associated, coiled-coil containing protein kinase 1	chr18:16878980	5	270	5	91.43	90.77	0.01	2.64
CBL	Cas-Br-M (murine) ecotropic retroviral transforming sequence	chr11:118608899	2	285	13	91.64	85.66	0.10	2.62
DHX8	DEAH (Asp-Glu-Ala-His) box polypeptide 8	chr17:38938069	12	351	7	90.97	96.36	-0.08	2.61
EP300	E1A binding protein p300	chr22:39890738	22	659	14	93.53	91.36	0.03	2.59
TCTE1	t-complex-associated-testis-expressed 1	chr6:44362906	2	357	2	89.78	86.67	0.05	2.57
OSM	oncostatin M	chr22:28991899	1	855	6	89.96	84.25	0.09	2.57
EPHA1	EPH receptor A1	chr7:142814115	2	710	7	92.23	93.58	-0.02	2.56
ALOX5	arachidonate 5-lipoxygenase	chr10:45260605	13	866	8	94.18	87.72	0.10	2.56
NOD1	nucleotide-binding oligomerization domain containing 1	chr7:30434604	13	910	10	90.68	88.14	0.04	2.56
RETNLB	resistin like beta	chr3:109957710	2	334	4	89.49	94.43	-0.08	2.55
NCBP1	nuclear cap binding protein subunit 1, 80kDa	chr9:99456790	11	780	14	88.63	68.68	0.37	2.53
GLI3	GLI family zinc finger 3	chr7:42031068	9	319	3	90.50	92.51	-0.03	2.53
CD164L2	CD164 sialomucin-like 2	chr1:27580979	3	425	6	92.69	98.23	-0.08	2.52
LRP2	low density lipoprotein receptor-related protein 2	chr2:169883098	3	419	10	90.88	85.11	0.09	2.49
VIPR2	vasoactive intestinal peptide receptor 2	chr7:158595119	3	144	1	92.71	84.19	0.14	2.48
PIK3R4	phosphoinositide-3-kinase, regulatory subunit 4	chr3:131934581	4	500	13	92.23	92.54	0.00	2.47
SLC20A2	solute carrier family 20 (phosphate transporter), member 2	chr8:42448063	2	713	17	91.02	90.04	0.02	2.47
SDPR	serum deprivation response	chr2:192419119	1	294	2	93.26	91.40	0.03	2.47
MRPS14	mitochondrial ribosomal protein S14	chr1:173253626	2	550	8	90.43	88.05	0.04	2.46

Gene symbol	Gene name	Location of latent 5’SS	Intron number	Position of latent 5’SS[a]	Number of stop codons[b]	Latent score[c]	Authentic score[c]	Log score ratio[d]	Fold change
ULK3	unc-51-like kinase 3 (C. elegans)	chr15:72918823	1	128	1	90.04	83.30	0.11	2.45
GMIP	GEM interacting protein	chr19:19611441	7	452	1	89.53	97.36	-0.12	2.44
CPT2	carnitine palmitoyltransferase 2	chr1:53440827	3	139	5	90.36	91.04	-0.01	2.43
LOH12CR1	loss of heterozygosity, 12, chromosomal region 1	chr12:12480832	3	847	23	91.66	87.88	0.06	2.42
SPATS1	spermatogenesis associated, serine-rich 1	chr6:44419570	1	622	13	90.49	63.91	0.50	2.42
LRP5	low density lipoprotein receptor-related protein 5	chr11:67941046	13	476	6	88.79	82.08	0.11	2.42
CPS1	carbamoyl-phosphate synthase 1, mitochondrial	chr2:211175958	16	660	13	89.48	92.94	-0.05	2.42
HERC1	hect (homologous to the E6-AP (UBE3A) carboxyl terminus) domain and RCC1 (CHC1)-like domain (RLD) 1	chr15:61753227	38	366	3	89.93	92.94	-0.05	2.41
DPP4	dipeptidyl-peptidase 4	chr2:162599350	9	569	11	92.12	84.34	0.13	2.41
ANKRD52	ankyrin repeat domain 52	chr12:54933920	8	197	3	91.33	85.49	0.10	2.40
KIAA1530	KIAA1530	chr4:1364329	11	312	3	89.14	96.22	-0.11	2.40
IRAK2	interleukin-1 receptor-associated kinase 2	chr3:10194862	2	159	4	91.49	85.29	0.10	2.40
SIRT4	sirtuin 4	chr12:119226601	2	358	4	89.93	84.04	0.10	2.40
ZNF157	zinc finger protein 157	chrX:47155471	3	354	2	93.26	100.00	-0.10	2.38
SLC17A2	solute carrier family 17 (sodium phosphate), member 2	chr6:26024448	8	443	11	91.43	88.51	0.05	2.38
C1orf115	chromosome 1 open reading frame 115	chr1:218931563	1	888	8	92.94	92.37	0.01	2.37
YPEL1	yippee-like 1 (Drosophila)	chr22:20394659	2	257	3	90.54	85.67	0.08	2.37
EIF4G2	eukaryotic translation initiation factor 4 gamma, 2	chr11:10776318	21	796	17	90.96	100.00	-0.14	2.37
GTPBP4	GTP binding protein 4	chr10:1032404	4	223	9	93.83	83.96	0.16	2.36
C7orf63	chromosome 7 open reading frame 63	chr7:89726383	3	971	22	90.43	70.86	0.35	2.36
TRAM2	translocation associated membrane protein 2	chr6:52507807	2	732	13	91.81	97.23	-0.08	2.35
SDHAF2	succinate dehydrogenase complex assembly factor 2	chr11:60962479	3	319	2	88.65	84.19	0.07	2.33
SH3PXD2A	SH3 and PX domains 2A	chr10:105376415	9	420	3	91.43	96.22	-0.07	2.32
LAMA5	laminin, alpha 5	chr20:60331252	45	654	5	89.94	78.89	0.19	2.32
LTC4S	leukotriene C4 synthase	chr5:179154085	1	341	4	89.46	90.97	-0.02	2.32
TCEB3	transcription elongation factor B (SIII), polypeptide 3 (110kDa, elongin A)	chr1:23943344	1	690	6	96.10	96.56	-0.01	2.31
DEF6	differentially expressed in FDCP 6 homolog (mouse)	chr6:35385783	2	219	2	89.93	91.50	-0.03	2.29
NFAM1	NFAT activating protein with ITAM motif 1	chr22:41112066	5	872	9	89.30	84.38	0.08	2.29

Gene symbol	Gene name	Location of latent 5'SS	Intron number	Position of latent 5'SS[a]	Number of stop codons[b]	Latent score[c]	Authentic score[c]	Log score ratio[d]	Fold change
HERC1	hect (homologous to the E6-AP (UBE3A) carboxyl terminus) domain and RCC1 (CHC1)-like domain (RLD) 1	chr15:61712181	67	918	18	100.00	91.40	0.13	2.29
DAGLA	diacylglycerol lipase, alpha	chr11:61252621	7	287	2	89.96	94.65	-0.07	2.29
ERCC4	excision repair cross-complementing rodent repair deficiency, complementation group 4	chr16:13930046	4	454	13	91.40	82.53	0.15	2.28
SIRPB2	signal-regulatory protein beta 2	chr20:1405736	4	246	3	89.96	96.83	-0.11	2.28
RAD21	RAD21 homolog (S. pombe)	chr8:117947543	2	462	13	89.45	92.94	-0.06	2.27
GHITM	growth hormone inducible transmembrane protein	chr10:85894322	4	481	4	88.65	82.92	0.10	2.27
PSME4	proteasome (prosome, macropain) activator subunit 4	chr2:53975028	34	619	10	90.50	95.52	-0.08	2.27
C20orf20	chromosome 20 open reading frame 20	chr20:60899507	2	480	7	90.54	92.13	-0.03	2.26
TSPEAR	thrombospondin-type laminin G domain and EAR repeats	chr21:44752564	10	945	8	88.72	91.77	-0.05	2.25
RAD18	RAD18 homolog (S. cerevisiae)	chr3:8906947	12	125	6	89.64	87.64	0.03	2.25
TOP2B	topoisomerase (DNA) II beta 180kDa	chr3:25644846	15	495	16	93.20	83.28	0.16	2.25
LRRC50	leucine rich repeat containing 50	chr16:82762160	8	698	13	91.84	88.92	0.05	2.25
RYR3	ryanodine receptor 3	chr15:31610652	4	494	9	91.64	91.87	0.00	2.24
DCC	deleted in colorectal carcinoma	chr18:48959850	9	367	6	88.64	85.31	0.06	2.24
CTC1	CTS telomere maintenance complex component 1	chr17:8081927	4	146	4	89.64	91.83	-0.03	2.24
TACO1	translational activator of mitochondrially encoded cytochrome c oxidase I	chr17:59032704	1	251	2	93.96	95.23	-0.02	2.22
MYST3	MYST histone acetyltransferase (monocytic leukemia) 3	chr8:41951004	8	374	7	93.27	90.77	0.04	2.22
HERC1	hect (homologous to the E6-AP (UBE3A) carboxyl terminus) domain and RCC1 (CHC1)-like domain (RLD) 1	chr15:61715773	64	983	24	89.93	88.64	0.02	2.22
NID1	nidogen 1	chr1:234267322	5	704	5	90.43	90.69	0.00	2.22
EFNA5	ephrin-A5	chr5:106790266	2	550	8	91.09	82.02	0.15	2.21
ARHGAP18	Rho GTPase activating protein 18	chr6:129968361	10	353	9	88.64	87.96	0.01	2.21
WNK4	WNK lysine deficient protein kinase 4	chr17:38191729	6	704	5	92.91	91.00	0.03	2.20
DHX29	DEAH (Asp-Glu-Ala-His) box polypeptide 29	chr5:54598316	23	406	8	90.50	89.65	0.01	2.20
TAF6L	TAF6-like RNA polymerase II, p300/CBP-associated factor (PCAF)-associated factor, 65kDa	chr11:62302701	5	319	3	89.04	93.99	-0.08	2.20
CISH	cytokine inducible SH2-containing protein	chr3:50623406	1	659	5	96.05	91.28	0.07	2.20

Gene symbol	Gene name	Location of latent 5'SS	Intron number	Position of latent 5'SS[a]	Number of stop codons[b]	Latent score[c]	Authentic score[c]	Log score ratio[d]	Fold change
POLR2H	polymerase (RNA) II (DNA directed) polypeptide H	chr3:185566053	3	346	7	89.46	97.36	-0.12	2.20
LPCAT4	lysophosphatidylcholine acyltransferase 4	chr15:32439293	12	310	2	91.99	87.88	0.07	2.20
NPHS1	nephrosis 1, congenital, Finnish type (nephrin)	chr19:41030520	11	262	6	90.69	84.49	0.10	2.19
C11orf58	chromosome 11 open reading frame 58	chr11:16726631	3	352	10	89.93	92.97	-0.05	2.19
FAF1	Fas (TNFRSF6) associated factor 1	chr1:51026083	4	176	3	97.82	89.14	0.13	2.17
ASF1B	ASF1 anti-silencing function 1 homolog B (S. cerevisiae)	chr19:14093048	3	295	4	89.46	92.64	-0.05	2.17
TEAD3	TEA domain family member 3	chr6:35554883	1	176	4	92.23	95.30	-0.05	2.17
BPI	bactericidal/permeability-increasing protein	chr20:36381106	6	815	11	91.96	97.36	-0.08	2.17
EXT1	exostosin 1	chr8:118916644	3	219	1	90.77	98.23	-0.11	2.16
DLL4	delta-like 4 (Drosophila)	chr15:39010398	3	227	2	89.92	99.13	-0.14	2.15
ZMYND12	zinc finger, MYND-type containing 12	chr1:42670467	7	933	21	88.72	85.67	0.05	2.15
PLEKHA6	pleckstrin homology domain containing, family A member 6	chr1:202483877	12	694	6	98.23	84.53	0.22	2.15
ANKS1A	ankyrin repeat and sterile alpha motif domain containing 1A	chr6:35136920	13	913	5	93.10	95.30	-0.03	2.14
ABCC2	ATP-binding cassette, sub-family C (CFTR/MRP), member 2	chr10:101595012	28	801	13	88.65	94.14	-0.09	2.14
TRNT1	tRNA nucleotidyl transferase, CCA-adding, 1	chr3:3157492	4	161	4	89.01	84.66	0.07	2.14
PLCG2	phospholipase C, gamma 2 (phosphatidylinositol-specific)	chr16:80474731	9	285	3	90.94	96.36	-0.08	2.14
TNKS2	tankyrase, TRF1-interacting ankyrin-related ADP-ribose polymerase 2	chr10:93595405	17	641	20	89.74	93.58	-0.06	2.14
ZNF552	zinc finger protein 552	chr19:63016909	1	981	13	91.09	91.83	-0.01	2.13
MYH7B	myosin, heavy chain 7B, cardiac muscle, beta	chr20:33031940	5	673	9	90.39	83.99	0.11	2.12
SEMA3C	sema domain, immunoglobulin domain (Ig), short basic domain, secreted, (semaphorin) 3C	chr7:80215275	17	874	15	88.70	96.95	-0.13	2.12
SMARCC1	SWI/SNF related, matrix associated, actin dependent regulator of chromatin, subfamily c, member 1	chr3:47744832	6	686	10	92.23	94.17	-0.03	2.12
BTBD8	BTB (POZ) domain containing 8	chr1:92377751	6	177	3	90.22	85.43	0.08	2.12
CARD9	caspase recruitment domain family, member 9	chr9:138384293	6	273	1	88.81	88.27	0.01	2.11
HPRT1	hypoxanthine phosphoribosyltransferase 1	chrX:133455594	6	309	9	93.12	92.94	0.00	2.11
VCP	valosin containing protein	chr9:35054385	5	862	15	89.88	96.95	-0.11	2.11

Gene symbol	Gene name	Location of latent 5'SS	Intron number	Position of latent 5'SS[a]	Number of stop codons[b]	Latent score[c]	Authentic score[c]	Log score ratio[d]	Fold change
CELSR2	cadherin, EGF LAG seven-pass G-type receptor 2 (flamingo homolog, Drosophila)	chr1:109603723	2	500	8	95.18	92.59	0.04	2.11
DUPD1	dual specificity phosphatase and pro isomerase domain containing 1	chr10:76472734	2	826	10	90.81	84.74	0.10	2.11
PIK3R2	phosphoinositide-3-kinase, regulatory subunit 2 (beta)	chr19:18134730	9	415	7	91.49	92.64	-0.02	2.11
PRPF31	PRP31 pre-mRNA processing factor 31 homolog (S. cerevisiae)	chr19:59317400	4	267	4	89.18	83.41	0.10	2.11
DIMT1L	DIM1 dimethyladenosine transferase 1-like (S. cerevisiae)	chr5:61732863	3	756	12	88.65	83.92	0.08	2.10
C10orf27	chromosome 10 open reading frame 27	chr10:72206088	7	826	16	100.00	92.66	0.11	2.10
MYOM2	myomesin (M-protein) 2, 165kDa	chr8:2034601	19	382	3	89.36	94.87	-0.09	2.09
TBC1D8	TBC1 domain family, member 8 (with GRAM domain)	chr2:101021146	5	208	2	96.36	86.47	0.16	2.09
ITGA2	integrin, alpha 2 (CD49B, alpha 2 subunit of VLA-2 receptor)	chr5:52377449	4	771	20	89.67	84.62	0.08	2.09
GALP	galanin-like peptide	chr19:61387011	5	619	2	89.53	88.59	0.02	2.09
TECPR2	tectonin beta-propeller repeat containing 2	chr14:101977010	11	312	6	95.05	89.93	0.08	2.09
SRSF4	serine/arginine-rich splicing factor 4	chr1:29359010	2	463	8	90.49	84.17	0.10	2.08
FRMD7	FERM domain containing 7	chrX:131055202	5	548	12	94.14	90.42	0.06	2.08
MOCOS	molybdenum cofactor sulfurase	chr18:32029587	2	281	1	92.26	84.94	0.12	2.08
C5orf4	chromosome 5 open reading frame 4	chr5:154183017	6	122	4	89.93	92.56	-0.04	2.08
DYNC1LI2	dynein, cytoplasmic 1, light intermediate chain 2	chr16:65340083	3	517	9	89.46	93.30	-0.06	2.07
ZFPM2	zinc finger protein, multitype 2	chr8:106643764	4	880	15	94.14	84.98	0.15	2.07
PRKD1	protein kinase D1	chr14:29264053	2	439	8	95.52	84.34	0.18	2.07
HNRPUL2	heterogeneous nuclear ribonucleoprotein U-like 2	chr11:62249865	1	801	23	91.02	91.99	-0.02	2.06
VRK1	vaccinia related kinase 1	chr14:96397421	11	597	9	91.77	90.54	0.02	2.06
MAT1A	methionine adenosyltransferase I, alpha	chr10:82023882	8	373	6	100.00	90.77	0.14	2.06
C18orf45	chromosome 18 open reading frame 45	chr18:19190053	12	502	13	88.65	89.53	-0.01	2.06
KISS1	KiSS-1 metastasis-suppressor	chr1:202428038	2	486	6	88.59	94.07	-0.09	2.05
SRRM4	serine/arginine repetitive matrix 4	chr12:118039867	4	672	8	90.97	84.13	0.11	2.05
RNF125	ring finger protein 125	chr18:27900176	5	207	6	89.93	87.08	0.05	2.05
MRPL16	mitochondrial ribosomal protein L16	chr11:59332928	2	975	20	91.14	95.23	-0.06	2.05

Gene symbol	Gene name	Location of latent 5'SS	Intron number	Position of latent 5'SS[a]	Number of stop codons[b]	Latent score[c]	Authentic score[c]	Log score ratio[d]	Fold change
PPFIA2	protein tyrosine phosphatase, receptor type, f polypeptide (PTPRF), interacting protein (liprin), alpha 2	chr12:80284454	14	563	8	92.51	82.71	0.16	2.04
ADAM8	ADAM metallopeptidase domain 8	chr10:134930681	21	164	1	89.10	96.83	-0.12	2.04
NPRL3	nitrogen permease regulator-like 3 (S. cerevisiae)	chr16:77819	11	877	12	92.23	94.41	-0.03	2.04
MMP14	matrix metallopeptidase 14 (membrane-inserted)	chr14:22376483	1	510	1	92.64	90.73	0.03	2.04
AHSA1	AHA1, activator of heat shock 90kDa protein ATPase homolog 1 (yeast)	chr14:76999276	4	422	9	89.46	84.00	0.09	2.04
COL4A2	collagen, type IV, alpha 2	chr13:109923723	29	226	3	91.43	96.95	-0.08	2.03
IGFN1	immunoglobulin-like and fibronectin type III domain containing 1	chr1:199451892	4	304	2	91.37	79.20	0.21	2.03
BTN2A3	butyrophilin, subfamily 2, member A3	chr6:26536311	4	110	2	92.30	72.52	0.35	2.03
FAM76B	family with sequence similarity 76, member B	chr11:95158201	4	729	18	91.37	90.63	0.01	2.03
ELOVL2	elongation of very long chain fatty acids (FEN1/Elo2, SUR4/Elo3, yeast)-like 2	chr6:11107843	4	462	13	92.23	92.59	-0.01	2.03
PARVG	parvin, gamma	chr22:42917981	7	103	1	93.76	82.56	0.18	2.03
BLMH	bleomycin hydrolase	chr17:25624561	7	624	12	92.26	84.49	0.13	2.02
LCP2	lymphocyte cytosolic protein 2 (SH2 domain containing leukocyte protein of 76kDa)	chr5:169629595	7	705	17	90.27	89.57	0.01	2.02
C7orf63	chromosome 7 open reading frame 63	chr7:89765197	16	118	1	91.23	86.47	0.08	2.02
ZUFSP	zinc finger with UFM1-specific peptidase domain	chr6:117093697	2	792	9	89.93	89.64	0.00	2.02
SMARCA5	SWI/SNF related, matrix associated, actin dependent regulator of chromatin, subfamily a, member 5	chr4:144665525	4	456	11	89.06	81.39	0.13	2.02
DEAF1	deformed epidermal autoregulatory factor 1 (Drosophila)	chr11:663884	10	651	9	94.41	87.75	0.11	2.01
INO80	INO80 homolog (S. cerevisiae)	chr15:39076685	29	333	4	88.65	87.51	0.02	2.01
PRSS12	protease, serine, 12 (neurotrypsin, motopsin)	chr4:119453195	7	608	12	91.09	88.72	0.04	2.01
ZNFX1	zinc finger, NFX1-type containing 1	chr20:47319678	3	207	4	90.06	97.23	-0.11	2.01
DHCR24	24-dehydrocholesterol reductase	chr1:55103265	6	298	3	91.43	97.36	-0.09	2.00
MET	met proto-oncogene (hepatocyte growth factor receptor)	chr7:116197942	12	862	15	90.68	89.46	0.02	2.00
CBL	Cas-Br-M (murine) ecotropic retroviral transforming sequence	chr11:118664329	12	464	12	93.09	100.00	-0.10	2.00
KIF5A	kinesin family member 5A	chr12:56261752	25	135	3	94.14	85.17	0.14	1.99

Gene symbol	Gene name	Location of latent 5'SS	Intron number	Position of latent 5'SS[a]	Number of stop codons[b]	Latent score[c]	Authentic score[c]	Log score ratio[d]	Fold change
CCDC18	coiled-coil domain containing 18	chr1:93464818	17	225	4	91.77	90.36	0.02	1.99
BIRC6	baculoviral IAP repeat containing 6	chr2:32556236	35	82	2	92.23	92.94	-0.01	1.99
C18orf8	chromosome 18 open reading frame 8	chr18:19353671	9	536	13	90.50	81.73	0.15	1.99
RARS	arginyl-tRNA synthetase	chr5:167866956	11	543	17	91.41	92.91	-0.02	1.98
BMPER	BMP binding endothelial regulator	chr7:33981453	7	533	7	89.94	86.16	0.06	1.98
POLR3GL	polymerase (RNA) III (DNA directed) polypeptide G (32kD)-like	chr1:144170144	3	864	11	91.43	89.22	0.04	1.98
PEX14	peroxisomal biogenesis factor 14	chr1:10610337	8	331	2	89.06	83.85	0.09	1.97
DAD1	defender against cell death 1	chr14:22126732	1	960	17	90.80	85.82	0.08	1.97
TSKS	testis-specific serine kinase substrate	chr19:54936380	9	573	13	90.27	78.90	0.19	1.96
ACBD3	acyl-CoA binding domain containing 3	chr1:224408138	6	832	17	94.18	94.14	0.00	1.96
TMED6	transmembrane emp24 protein transport domain containing 6	chr16:67942438	1	506	10	94.60	91.89	0.04	1.95
DPF3	D4, zinc and double PHD fingers, family 3	chr14:72429713	1	804	6	88.72	84.81	0.07	1.95
COL5A1	collagen, type V, alpha 1	chr9:136764378	9	585	11	94.89	93.09	0.03	1.95
FAAH	fatty acid amide hydrolase	chr1:46649775	11	663	9	89.94	89.57	0.01	1.95
PSMD11	proteasome (prosome, macropain) 26S subunit, non-ATPase, 11	chr17:27829295	9	582	6	90.99	98.00	-0.11	1.95
EXTL3	exostoses (multiple)-like 3	chr8:28657516	6	867	10	93.53	96.22	-0.04	1.95
C5	complement component 5	chr9:122770543	33	616	13	94.18	96.83	-0.04	1.95
DHTKD1	dehydrogenase E1 and transketolase domain containing 1	chr10:12151947	1	756	4	92.64	84.91	0.13	1.95
SUSD1	sushi domain containing 1	chr9:113860375	14	153	3	88.90	85.16	0.06	1.95
LBP	lipopolysaccharide binding protein	chr20:36413711	3	956	8	91.43	86.86	0.07	1.94
ASPHD1	aspartate beta-hydroxylase domain containing 1	chr16:29824540	2	754	19	90.04	84.63	0.09	1.94
SLC34A1	solute carrier family 34 (sodium phosphate), member 1	chr5:176748533	8	555	8	90.46	99.13	-0.13	1.94
KLHL35	kelch-like 35 (Drosophila)	chr11:74816389	2	745	13	97.36	93.09	0.06	1.94
C3orf19	chromosome 3 open reading frame 19	chr3:14682031	6	398	9	88.96	86.16	0.05	1.94
C19orf75	chromosome 19 open reading frame 75	chr19:56459576	2	417	9	89.90	91.36	-0.02	1.93
CCDC37	coiled-coil domain containing 37	chr3:127618584	5	544	5	90.97	92.13	-0.02	1.93
COL1A2	collagen, type I, alpha 2	chr7:93893951	46	174	3	96.22	85.11	0.18	1.92
TMPRSS2	transmembrane protease, serine 2	chr21:41766678	9	443	3	89.11	83.55	0.09	1.92

Gene symbol	Gene name	Location of latent 5'SS	Intron number	Position of latent 5'SS[a]	Number of stop codons[b]	Latent score[c]	Authentic score[c]	Log score ratio[d]	Fold change
KCNQ3	potassium voltage-gated channel, KQT-like subfamily, member 3	chr8:133266939	2	580	6	88.65	92.54	-0.06	1.91
LRP5	low density lipoprotein receptor-related protein 5	chr11:67931448	9	592	13	88.65	84.60	0.07	1.91
ARL2	ADP-ribosylation factor-like 2	chr11:64543311	3	531	7	91.49	89.93	0.02	1.91
SRBD1	S1 RNA binding domain 1	chr2:45626961	14	413	13	98.87	93.53	0.08	1.90
NAP1L4	nucleosome assembly protein 1-like 4	chr11:2947033	7	575	10	91.84	97.70	-0.09	1.90
TAF11	TAF11 RNA polymerase II, TATA box binding protein (TBP)-associated factor, 28kDa	chr6:34958510	2	176	3	90.50	86.47	0.07	1.89
PIM2	pim-2 oncogene	chrX:48657033	4	207	4	92.66	92.40	0.00	1.89
COL4A1	collagen, type IV, alpha 1	chr13:109657382	13	368	5	92.04	90.17	0.03	1.89
TLL2	tolloid-like 2	chr10:98136282	14	417	6	93.30	84.49	0.14	1.89
GADD45A	growth arrest and DNA-damage-inducible, alpha	chr1:67925606	3	749	13	89.63	92.83	-0.05	1.88
NBEAL2	neurobeachin-like 2	chr3:47021450	38	367	5	90.17	90.49	-0.01	1.88
LPO	lactoperoxidase	chr17:53680613	4	616	9	90.27	84.91	0.09	1.88
LRP2	low density lipoprotein receptor-related protein 2	chr2:169746441	51	481	4	92.07	75.49	0.29	1.88
SLC36A1	solute carrier family 36 (proton/amino acid symporter), member 1	chr5:150825269	5	347	5	92.83	91.09	0.03	1.87
NOL11	nucleolar protein 11	chr17:63165175	14	243	6	89.46	70.15	0.35	1.87
C4orf34	chromosome 4 open reading frame 34	chr4:39249934	3	495	4	89.46	90.42	-0.02	1.87
ADAM23	ADAM metallopeptidase domain 23	chr2:207019152	2	660	19	90.35	91.84	-0.02	1.87
KCNB2	potassium voltage-gated channel, Shab-related subfamily, member 2	chr8:73643775	2	674	8	91.41	90.17	0.02	1.87
TMEM126A	transmembrane protein 126A	chr11:85043377	3	430	5	88.65	92.54	-0.06	1.86
CENPV	centromere protein V	chr17:16193843	2	126	4	89.19	92.04	-0.05	1.86
AGPS	alkylglycerone phosphate synthase	chr2:177966507	1	485	10	93.58	92.69	0.01	1.86
EVC	Ellis van Creveld syndrome	chr4:5809278	11	289	1	96.36	86.68	0.15	1.85
KIAA1147	KIAA1147	chr7:141031097	3	649	10	89.93	85.47	0.07	1.85
PCNXL2	pecanex-like 2 (Drosophila)	chr1:231438450	3	763	17	90.68	89.90	0.01	1.85
IRAK2	interleukin-1 receptor-associated kinase 2	chr3:10243485	10	369	6	91.43	93.53	-0.03	1.85
ERCC4	excision repair cross-complementing rodent repair deficiency, complementation group 4	chr16:13934491	6	849	13	95.69	85.16	0.17	1.84
TCTN2	tectonic family member 2	chr12:122723090	3	459	6	90.01	91.79	-0.03	1.84
TM6SF2	transmembrane 6 superfamily member 2	chr19:19241185	5	310	1	88.86	89.14	0.00	1.84

Gene symbol	Gene name	Location of latent 5’SS	Intron number	Position of latent 5’SS[a]	Number of stop codons[b]	Latent score[c]	Authentic score[c]	Log score ratio[d]	Fold change
DEPTOR	DEP domain containing MTOR-interacting protein	chr8:121083454	5	325	7	89.46	91.77	-0.04	1.84
ZNF236	zinc finger protein 236	chr18:72721949	8	690	6	95.05	89.90	0.08	1.84
HAUS5	HAUS augmin-like complex, subunit 5	chr19:40798670	6	543	8	95.18	89.93	0.08	1.84
SPTA1	spectrin, alpha, erythrocytic 1 (elliptocytosis 2)	chr1:156848686	51	544	8	93.76	82.77	0.18	1.83
WDR63	WD repeat domain 63	chr1:85369016	22	634	27	89.49	84.68	0.08	1.83
PCSK5	proprotein convertase subtilisin/kexin type 5	chr9:78133684	5	710	4	92.11	83.38	0.14	1.83
YIPF1	Yip1 domain family, member 1	chr1:54120834	4	539	11	90.50	79.47	0.19	1.83
CA2	carbonic anhydrase II	chr8:86564319	1	724	4	91.50	93.73	-0.03	1.82
BIRC6	baculoviral IAP repeat containing 6	chr2:32566926	41	555	21	89.65	91.30	-0.03	1.82
GATA5	GATA binding protein 5	chr20:60481717	3	136	1	90.14	94.43	-0.07	1.82
FBN1	fibrillin 1	chr15:46583174	17	101	1	91.83	94.07	-0.03	1.82
COL4A1	collagen, type IV, alpha 1	chr13:109661452	8	542	4	95.23	85.54	0.15	1.82
ITCH	itchy E3 ubiquitin protein ligase homolog (mouse)	chr20:32544729	23	667	18	91.83	85.43	0.10	1.82
NAB2	NGFI-A binding protein 2 (EGR1 binding protein 2)	chr12:55769733	1	330	1	91.50	83.26	0.14	1.82
TMEM206	transmembrane protein 206	chr1:210626604	3	251	1	90.33	86.05	0.07	1.82
RAB2A	RAB2A, member RAS oncogene family	chr8:61634315	2	280	6	91.40	90.91	0.01	1.82
RNF213	ring finger protein 213	chr17:75957464	19	927	7	92.12	83.91	0.13	1.82
TRIP4	thyroid hormone receptor interactor 4	chr15:62503674	10	268	3	88.64	88.32	0.01	1.81
TOX	thymocyte selection-associated high mobility group box	chr8:60014234	3	180	6	88.64	90.36	-0.03	1.81
COMMD9	COMM domain containing 9	chr11:36267325	1	162	1	89.01	98.87	-0.15	1.81
NUAK2	NUAK family, SNF1-like kinase, 2	chr1:203556712	1	436	6	89.25	94.43	-0.08	1.80
KDM4A	lysine (K)-specific demethylase 4A	chr1:43904634	6	632	8	90.43	83.06	0.12	1.80
KCTD20	potassium channel tetramerisation domain containing 20	chr6:36551469	3	653	15	91.81	91.09	0.01	1.80
SLC24A1	solute carrier family 24 (sodium/potassium/calcium exchanger), member 1	chr15:63719692	2	598	5	91.86	96.56	-0.07	1.80
TMEM206	transmembrane protein 206	chr1:210624997	4	241	1	89.92	84.95	0.08	1.80
EIF2C2	eukaryotic translation initiation factor 2C, 2	chr8:141622852	14	641	7	94.18	82.87	0.18	1.79
ZBTB40	zinc finger and BTB domain containing 40	chr1:22711799	11	586	6	91.36	94.14	-0.04	1.79
TP53TG5	TP53 target 5	chr20:43437064	4	28	0	91.96	93.27	-0.02	1.79
CLPTM1	cleft lip and palate associated transmembrane protein 1	chr19:50181361	6	961	5	89.46	91.41	-0.03	1.78

Gene symbol	Gene name	Location of latent 5'SS	Intron number	Position of latent 5'SS[a]	Number of stop codons[b]	Latent score[c]	Authentic score[c]	Log score ratio[d]	Fold change
ST8SIA3	ST8 alpha-N-acetyl-neuraminide alpha-2,8-sialyltransferase 3	chr18:53171544	1	291	2	91.99	94.87	-0.04	1.78
STK38L	serine/threonine kinase 38 like	chr12:27342403	2	350	9	89.93	84.84	0.08	1.78
CA11	carbonic anhydrase XI	chr19:53838946	3	549	8	88.65	97.10	-0.13	1.78
NUP35	nucleoporin 35kDa	chr2:183731529	7	146	2	90.83	91.78	-0.02	1.78
LPCAT4	lysophosphatidylcholine acyltransferase 4	chr15:32444773	2	278	3	93.12	90.73	0.04	1.77
PTPN4	protein tyrosine phosphatase, non-receptor type 4 (megakaryocyte)	chr2:120394927	12	641	15	92.54	95.30	-0.04	1.77
SLC6A3	solute carrier family 6 (neurotransmitter transporter, dopamine), member 3	chr5:1467080	8	725	6	99.13	91.43	0.12	1.77
SFPQ	splicing factor proline/glutamine-rich	chr1:35425136	9	52	0	88.80	84.23	0.08	1.77
ZBTB41	zinc finger and BTB domain containing 41	chr1:195411685	7	631	22	90.96	84.31	0.11	1.76
SLC25A12	solute carrier family 25 (mitochondrial carrier, Aralar), member 12	chr2:172374568	12	411	14	89.25	86.16	0.05	1.76
NLRP2	NLR family, pyrin domain containing 2	chr19:60181756	4	754	7	91.43	93.27	-0.03	1.76
CTNND2	catenin (cadherin-associated protein), delta 2 (neural plakophilin-related arm-repeat protein)	chr5:11288802	10	1000	20	90.43	89.22	0.02	1.76
DRP2	dystrophin related protein 2	chrX:100384837	9	211	3	89.93	81.43	0.14	1.76
TNR	tenascin R (restrictin, janusin)	chr1:173597736	12	685	9	89.14	91.64	-0.04	1.76
ZNF26	zinc finger protein 26	chr12:132074424	1	893	13	95.18	91.36	0.06	1.75
RFX1	regulatory factor X, 1 (influences HLA class II expression)	chr19:13939618	12	758	8	93.20	87.72	0.09	1.75
REM1	RAS (RAD and GEM)-like GTP-binding 1	chr20:29528802	2	554	8	91.84	91.33	0.01	1.75
RAB10	RAB10, member RAS oncogene family	chr2:26203937	4	332	5	91.00	89.77	0.02	1.75
LOXL1	lysyl oxidase-like 1	chr15:72027052	4	436	2	90.29	83.85	0.11	1.74
CTNND2	catenin (cadherin-associated protein), delta 2 (neural plakophilin-related arm-repeat protein)	chr5:11163632	14	337	7	88.59	89.19	-0.01	1.74
EVI5	ecotropic viral integration site 5	chr1:92942335	3	364	8	93.20	82.46	0.18	1.74
KIAA0247	KIAA0247	chr14:69246115	5	542	10	98.87	93.30	0.08	1.74
NPC1	Niemann-Pick disease, type C1	chr18:19366561	24	755	12	89.90	92.83	-0.05	1.74
SLC25A12	solute carrier family 25 (mitochondrial carrier, Aralar), member 12	chr2:172408221	5	903	14	90.46	96.83	-0.10	1.73
SMG5	Smg-5 homolog, nonsense mediated mRNA decay factor (C. elegans)	chr1:154513739	3	600	12	89.27	97.10	-0.12	1.73

Gene symbol	Gene name	Location of latent 5'SS	Intron number	Position of latent 5'SS[a]	Number of stop codons[b]	Latent score[c]	Authentic score[c]	Log score ratio[d]	Fold change
TTC33	tetratricopeptide repeat domain 33	chr5:40782295	2	361	8	92.54	92.94	-0.01	1.73
NUP153	nucleoporin 153kDa	chr6:17747904	15	244	10	93.10	92.54	0.01	1.73
C19orf30	chromosome 19 open reading frame 30	chr19:4721230	2	462	6	97.23	90.46	0.10	1.73
CEP120	centrosomal protein 120kDa	chr5:122786061	2	481	9	92.07	95.69	-0.06	1.73
OGDHL	oxoglutarate dehydrogenase-like	chr10:50630384	4	305	3	91.66	88.59	0.05	1.73
ABCC8	ATP-binding cassette, sub-family C (CFTR/MRP), member 8	chr11:17405607	15	382	6	92.23	90.12	0.03	1.73
ABCC2	ATP-binding cassette, sub-family C (CFTR/MRP), member 2	chr10:101560537	14	573	10	92.23	83.45	0.14	1.73
DHX35	DEAH (Asp-Glu-Ala-His) box polypeptide 35	chr20:37072693	13	233	3	92.91	91.34	0.02	1.73
CBLC	Cas-Br-M (murine) ecotropic retroviral transforming sequence c	chr19:49978091	4	504	12	93.73	90.77	0.05	1.73
SCNN1B	sodium channel, nonvoltage-gated 1, beta	chr16:23272568	3	673	16	88.90	88.22	0.01	1.72
RFX6	regulatory factor X, 6	chr6:117310759	4	476	11	91.41	89.01	0.04	1.72
FBXO2	F-box protein 2	chr1:11636485	1	477	1	89.49	85.20	0.07	1.72
CRAMP1L	Crm, cramped-like (Drosophila)	chr16:1628556	4	648	6	89.11	84.24	0.08	1.72
CX3CL1	chemokine (C-X3-C motif) ligand 1	chr16:55964332	1	282	4	91.43	95.30	-0.06	1.72
HDDC2	HD domain containing 2	chr6:125639799	5	147	4	92.99	83.66	0.15	1.71
TNC	tenascin C	chr9:116837760	21	437	5	93.76	83.91	0.16	1.71
FAM83E	family with sequence similarity 83, member E	chr19:53798377	4	185	2	92.11	94.18	-0.03	1.71
TMEM146	transmembrane protein 146	chr19:5672761	1	943	9	89.93	83.97	0.10	1.71
CBL	Cas-Br-M (murine) ecotropic retroviral transforming sequence	chr11:118674691	15	232	5	88.86	92.04	-0.05	1.71
ZNF426	zinc finger protein 426	chr19:9504223	6	297	4	90.01	98.00	-0.12	1.71
INSRR	insulin receptor-related receptor	chr1:155081309	12	182	2	95.23	82.79	0.20	1.71
EIF3M	eukaryotic translation initiation factor 3, subunit M	chr11:32567059	3	206	5	89.49	84.68	0.08	1.71
VWCE	von Willebrand factor C and EGF domains	chr11:60800114	11	526	8	92.71	100.00	-0.11	1.71
CMYA5	cardiomyopathy associated 5	chr5:79123506	11	859	14	92.94	86.50	0.10	1.71
C21orf128	chromosome 21 open reading frame 128	chr21:42401205	1	269	2	91.43	92.23	-0.01	1.70
ALG14	asparagine-linked glycosylation 14 homolog (S. cerevisiae)	chr1:95310401	1	505	6	89.06	95.74	-0.10	1.70
ODZ1	odz, odd Oz/ten-m homolog 1(Drosophila)	chrX:123367176	25	671	15	91.36	96.10	-0.07	1.70
FBXL2	F-box and leucine-rich repeat protein 2	chr3:33395730	13	494	6	88.65	94.43	-0.09	1.70

Gene symbol	Gene name	Location of latent 5'SS	Intron number	Position of latent 5'SS[a]	Number of stop codons[b]	Latent score[c]	Authentic score[c]	Log score ratio[d]	Fold change
IFI30	interferon, gamma-inducible protein 30	chr19:18146368	1	586	2	89.90	92.37	-0.04	1.70
TOP1MT	topoisomerase (DNA) I, mitochondrial	chr8:144470485	10	782	6	96.36	90.39	0.09	1.70
FAM70B	family with sequence similarity 70, member B	chr13:113611154	5	396	5	99.13	94.65	0.07	1.70
EMID2	EMI domain containing 2	chr7:100970908	5	859	15	89.46	94.87	-0.08	1.70
EVC	Ellis van Creveld syndrome	chr4:5863508	20	431	2	91.36	91.30	0.00	1.69
EPRS	glutamyl-prolyl-tRNA synthetase	chr1:218245780	15	290	1	89.38	88.79	0.01	1.69
PPM1D	protein phosphatase, Mg2+/Mn2+ dependent, 1D	chr17:56089182	5	199	2	89.82	85.48	0.07	1.69
TMPRSS4	transmembrane protease, serine 4	chr11:117491518	10	314	4	90.96	92.64	-0.03	1.69
SUPV3L1	suppressor of var1, 3-like 1 (S. cerevisiae)	chr10:70632472	12	192	6	92.64	85.73	0.11	1.69
BAIAP2L1	BAI1-associated protein 2-like 1	chr7:97761085	13	236	3	89.25	92.98	-0.06	1.69
ATP10A	ATPase, class V, type 10A	chr15:23576788	3	470	4	91.99	86.86	0.08	1.69
PBX4	pre-B-cell leukemia homeobox 4	chr19:19542218	3	176	3	88.94	88.65	0.00	1.69
LRRC40	leucine rich repeat containing 40	chr1:70418521	5	835	18	88.63	84.32	0.07	1.69
ULK1	unc-51-like kinase 1 (C. elegans)	chr12:130970318	25	231	1	90.36	91.99	-0.03	1.69
FAM193A	family with sequence similarity 193, member A	chr4:2668467	16	350	5	92.11	85.67	0.10	1.68
HIRA	HIR histone cell cycle regulation defective homolog A (S. cerevisiae)	chr22:17717915	24	965	12	91.17	88.40	0.04	1.68
CPD	carboxypeptidase D	chr17:25795616	11	502	17	97.70	93.76	0.06	1.68
TMEM63A	transmembrane protein 63A	chr1:224115771	13	769	9	91.53	87.35	0.07	1.68
DHCR24	24-dehydrocholesterol reductase	chr1:55124880	1	269	1	92.23	96.83	-0.07	1.68
FAM55C	family with sequence similarity 55, member C	chr3:103018889	7	355	10	89.93	85.63	0.07	1.68
GTF2H3	general transcription factor IIH, polypeptide 3, 34kDa	chr12:122701653	5	98	3	90.68	69.11	0.39	1.68
DIP2C	DIP2 disco-interacting protein 2 homolog C (Drosophila)	chr10:450977	7	731	16	91.49	95.23	-0.06	1.67
SKA2	spindle and kinetochore associated complex subunit 2	chr17:54587100	1	173	3	93.99	93.30	0.01	1.67
TRAF1	TNF receptor-associated factor 1	chr9:122727214	2	820	14	89.27	95.18	-0.09	1.67
NDUFAB1	NADH dehydrogenase (ubiquinone) 1, alpha/beta subcomplex, 1, 8kDa	chr16:23503681	3	476	3	96.36	89.14	0.11	1.67
MYSM1	Myb-like, SWIRM and MPN domains 1	chr1:58914972	5	213	3	88.64	82.90	0.10	1.67
PHOX2B	paired-like homeobox 2b	chr4:41443827	2	295	1	89.76	91.83	-0.03	1.67
ARL6IP1	ADP-ribosylation factor-like 6 interacting protein 1	chr16:18712653	5	768	15	91.99	95.60	-0.06	1.67

Gene symbol	Gene name	Location of latent 5'SS	Intron number	Position of latent 5'SS[a]	Number of stop codons[b]	Latent score[c]	Authentic score[c]	Log score ratio[d]	Fold change
GTPBP1	GTP binding protein 1	chr22:37456437	11	825	8	91.99	96.83	-0.07	1.66
EXOSC2	exosome component 2	chr9:132559905	1	785	11	89.21	87.69	0.02	1.66
TRMT1L	TRM1 tRNA methyltransferase 1-like	chr1:183363652	11	771	15	88.65	87.20	0.02	1.66
XYLT2	xylosyltransferase II	chr17:45791537	10	638	8	92.11	96.56	-0.07	1.66
C19orf56	chromosome 19 open reading frame 56	chr19:12640577	2	364	2	91.64	90.97	0.01	1.65
KIAA0247	KIAA0247	chr14:69240789	3	728	12	88.64	86.03	0.04	1.65
AVL9	AVL9 homolog (S. cerevisiase)	chr7:32582686	13	478	12	89.93	89.48	0.01	1.65
KIAA0302	KIAA0302	chr11:66210783	4	257	3	92.37	86.09	0.10	1.65
ATP1A3	ATPase, Na+/K+ transporting, alpha 3 polypeptide	chr19:47165828	18	348	3	96.36	88.27	0.13	1.65
HERC1	hect (homologous to the E6-AP (UBE3A) carboxyl terminus) domain and RCC1 (CHC1)-like domain (RLD) 1	chr15:61827666	10	952	29	99.13	97.23	0.03	1.64
EDAR	ectodysplasin A receptor	chr2:108888333	11	862	7	90.49	96.56	-0.09	1.64
BIRC6	baculoviral IAP repeat containing 6	chr2:32555756	34	841	24	88.96	90.17	-0.02	1.64
NKAIN4	Na+/K+ transporting ATPase interacting 4	chr20:61350745	2	986	6	90.90	92.26	-0.02	1.64
SUPV3L1	suppressor of var1, 3-like 1 (S. cerevisiae)	chr10:70628781	9	468	15	95.18	87.13	0.13	1.64
CCND1	cyclin D1	chr11:69173058	4	968	6	90.49	95.74	-0.08	1.64
CDC42BPB	CDC42 binding protein kinase beta (DMPK-like)	chr14:102522177	6	399	6	92.23	81.43	0.18	1.64
KIF7	kinesin family member 7	chr15:87974010	12	511	11	95.23	90.97	0.07	1.63
SORL1	sortilin-related receptor, L(DLR class) A repeats containing	chr11:120931402	18	166	1	93.58	91.89	0.03	1.63
RNASEK	ribonuclease, RNase K	chr17:6857247	1	444	3	91.37	88.27	0.05	1.63
OGDHL	oxoglutarate dehydrogenase-like	chr10:50621615	14	430	3	89.94	92.66	-0.04	1.63
LMAN1	lectin, mannose-binding, 1	chr18:55150683	11	619	9	90.27	86.40	0.06	1.62
MAPK4	mitogen-activated protein kinase 4	chr18:46507031	5	489	11	90.50	82.79	0.13	1.62
MAN1A1	mannosidase, alpha, class 1A, member 1	chr6:119663998	4	853	15	93.46	93.13	0.01	1.62
TELO2	TEL2, telomere maintenance 2, homolog (S. cerevisiae)	chr16:1493271	15	268	4	97.36	89.38	0.12	1.62
STXBP3	syntaxin binding protein 3	chr1:109139487	13	436	6	89.77	94.07	-0.07	1.62
HERC5	hect domain and RLD 5	chr4:89597897	1	190	4	90.41	87.80	0.04	1.62
IQGAP1	IQ motif containing GTPase activating protein 1	chr15:88799654	14	867	16	91.43	86.67	0.08	1.62
OSGEPL1	O-sialoglycoprotein endopeptidase-like 1	chr2:190325416	7	207	5	94.73	85.85	0.14	1.62
RND1	Rho family GTPase 1	chr12:47545414	1	283	1	97.36	94.65	0.04	1.61
SUSD1	sushi domain containing 1	chr9:113879841	12	797	13	88.91	92.54	-0.06	1.61

Gene symbol	Gene name	Location of latent 5'SS	Intron number	Position of latent 5'SS[a]	Number of stop codons[b]	Latent score[c]	Authentic score[c]	Log score ratio[d]	Fold change
LOXL2	lysyl oxidase-like 2	chr8:23234749	7	838	10	93.00	88.05	0.08	1.61
ZUFSP	zinc finger with UFM1-specific peptidase domain	chr6:117088090	3	501	9	93.00	92.94	0.00	1.61
SKIV2L2	superkiller viralicidic activity 2-like 2 (S. cerevisiae)	chr5:54755157	26	591	13	89.25	90.27	-0.02	1.61
BAG2	BCL2-associated athanogene 2	chr6:57155253	2	356	4	90.99	90.96	0.00	1.61
TG	thyroglobulin	chr8:133993791	19	832	10	91.53	84.98	0.11	1.61
TWSG1	twisted gastrulation homolog 1 (Drosophila)	chr18:9387245	4	702	9	88.59	91.09	-0.04	1.61
KIF26B	kinesin family member 26B	chr1:243914941	11	622	2	90.46	97.10	-0.10	1.61
SDF2	stromal cell-derived factor 2	chr17:24011914	1	956	13	92.11	90.06	0.03	1.60
CHIC2	cysteine-rich hydrophobic domain 2	chr4:54609488	3	390	12	88.64	87.54	0.02	1.60
CHAF1B	chromatin assembly factor 1, subunit B (p60)	chr21:36680923	2	494	6	91.64	84.87	0.11	1.60
IGFBP4	insulin-like growth factor binding protein 4	chr17:35853979	1	118	1	94.14	93.53	0.01	1.60
CLPX	ClpX caseinolytic peptidase X homolog (E. coli)	chr15:63235758	9	476	15	91.99	90.61	0.02	1.60
NUP133	nucleoporin 133kDa	chr1:227689393	10	442	3	92.98	83.77	0.15	1.60
EVC	Ellis van Creveld syndrome	chr4:5861439	18	485	7	95.23	83.91	0.18	1.60
SCNN1G	sodium channel, nonvoltage-gated 1, gamma	chr16:23116480	6	232	1	95.18	92.54	0.04	1.60
CMYA5	cardiomyopathy associated 5	chr5:79077520	4	487	10	89.06	86.16	0.05	1.60
PVRL4	poliovirus receptor-related 4	chr1:159310882	5	142	3	89.25	88.61	0.01	1.59
EFHC1	EF-hand domain (C-terminal) containing 1	chr6:52452936	9	393	15	92.30	92.07	0.00	1.59
RAB6B	RAB6B, member RAS oncogene family	chr3:135041993	4	825	7	91.64	92.04	-0.01	1.59
SAP30	Sin3A-associated protein, 30kDa	chr4:174530182	1	960	11	89.37	84.81	0.08	1.59
ASB13	ankyrin repeat and SOCS box containing 13	chr10:5734247	2	584	4	90.94	99.13	-0.12	1.59
GRAMD3	GRAM domain containing 3	chr5:125844623	8	326	6	90.33	95.30	-0.08	1.59
HDAC4	histone deacetylase 4	chr2:239676453	18	188	3	88.70	90.27	-0.03	1.59
CCDC69	coiled-coil domain containing 69	chr5:150547022	5	117	1	88.78	96.36	-0.12	1.59
AX747133	cDNA FLJ34375 fis, clone FEBRA2017533.	chr18:33107941	1	330	4	91.99	86.47	0.09	1.59
GNL3L	guanine nucleotide binding protein-like 3 (nucleolar)-like	chrX:54587275	7	776	15	89.45	85.31	0.07	1.58
FAF2	Fas associated factor family member 2	chr5:175854386	7	505	10	89.74	93.58	-0.06	1.58
PRPF8	PRP8 pre-mRNA processing factor 8 homolog (S. cerevisiae)	chr17:1525461	19	202	3	88.92	83.92	0.08	1.58
FAM116A	family with sequence similarity 116, member A	chr3:57590985	18	202	8	93.10	79.54	0.23	1.58

Gene symbol	Gene name	Location of latent 5'SS	Intron number	Position of latent 5'SS[a]	Number of stop codons[b]	Latent score[c]	Authentic score[c]	Log score ratio[d]	Fold change
LANCL1	LanC lantibiotic synthetase component C-like 1 (bacterial)	chr2:211048873	2	411	11	92.40	93.76	-0.02	1.58
C11orf21	chromosome 11 open reading frame 21	chr11:2278710	1	851	5	91.30	94.65	-0.05	1.58
ADAMTS1	ADAM metallopeptidase with thrombospondin type 1 motif, 1	chr21:27135419	3	628	7	90.43	95.60	-0.08	1.58
BBS2	Bardet-Biedl syndrome 2	chr16:55091822	11	444	7	88.65	89.06	-0.01	1.58
TRIM21	tripartite motif containing 21	chr11:4366959	3	500	14	90.46	96.10	-0.09	1.58
TBK1	TANK-binding kinase 1	chr12:63176060	15	239	6	89.46	86.04	0.06	1.58
SCGN	secretagogin, EF-hand calcium binding protein	chr6:25791030	7	818	19	90.05	98.00	-0.12	1.58
CCDC134	coiled-coil domain containing 134	chr22:40539644	5	250	2	92.13	93.53	-0.02	1.58
ZNF689	zinc finger protein 689	chr16:30527354	2	992	11	92.23	89.86	0.04	1.57
SAT1	spermidine/spermine N1-acetyltransferase 1	chrX:23712388	3	468	10	90.05	88.79	0.02	1.57
P4HA3	prolyl 4-hydroxylase, alpha polypeptide III	chr11:73674094	7	407	4	90.46	90.49	0.00	1.57
ZNF330	zinc finger protein 330	chr4:142373854	9	609	14	91.53	91.14	0.01	1.57
SORL1	sortilin-related receptor, L(DLR class) A repeats containing	chr11:120946711	23	523	11	91.55	82.87	0.14	1.57
NUP37	nucleoporin 37kDa	chr12:100993789	7	915	18	89.01	90.06	-0.02	1.57
EME1	essential meiotic endonuclease 1 homolog 1 (S. pombe)	chr17:45812513	7	587	13	88.61	92.04	-0.05	1.56
HELZ	helicase with zinc finger	chr17:62587054	16	420	9	92.86	91.96	0.01	1.56
XPNPEP2	X-prolyl aminopeptidase (aminopeptidase P) 2, membrane-bound	chrX:128709855	7	446	6	92.11	90.96	0.02	1.56
LAD1	ladinin 1	chr1:199621636	3	449	2	90.41	83.32	0.12	1.56
M6PR	mannose-6-phosphate receptor (cation dependent)	chr12:8985999	6	279	5	96.22	86.47	0.15	1.56
PARVG	parvin, gamma	chr22:42920069	8	753	2	92.64	97.36	-0.07	1.56
C10orf92	chromosome 10 open reading frame 92	chr10:134473221	19	681	5	91.96	97.10	-0.08	1.55
LPCAT4	lysophosphatidylcholine acyltransferase 4	chr15:32438962	13	119	1	88.96	82.02	0.12	1.55
CCDC120	coiled-coil domain containing 120	chrX:48807435	4	360	6	89.93	90.92	-0.02	1.55
KIAA1199	KIAA1199	chr15:78968653	8	462	11	89.01	96.22	-0.11	1.55
WWC3	WWC family member 3	chrX:10038447	9	388	4	89.96	92.23	-0.04	1.55
COMMD9	COMM domain containing 9	chr11:36258353	2	484	12	91.09	90.73	0.01	1.55
ART1	ADP-ribosyltransferase 1	chr11:3638843	3	675	10	97.10	91.37	0.09	1.55
SORCS2	sortilin-related VPS10 domain containing receptor 2	chr4:7766107	15	628	6	89.01	92.11	-0.05	1.54
NUP133	nucleoporin 133kDa	chr1:227668140	16	863	14	89.88	82.77	0.12	1.54

Gene symbol	Gene name	Location of latent 5'SS	Intron number	Position of latent 5'SS[a]	Number of stop codons[b]	Latent score[c]	Authentic score[c]	Log score ratio[d]	Fold change
FGD1	FYVE, RhoGEF and PH domain containing 1	chrX:54537523	1	760	8	93.20	94.43	-0.02	1.54
TECTA	tectorin alpha	chr11:120506802	9	673	14	90.97	83.74	0.12	1.54
PROZ	protein Z, vitamin K-dependent plasma glycoprotein	chr13:112873780	7	936	9	90.39	91.55	-0.02	1.54
UNKL	unkempt homolog (Drosophila)-like	chr16:1390996	4	573	2	91.36	91.09	0.00	1.54
APOA2	apolipoprotein A-II	chr1:159459557	2	206	2	90.12	93.27	-0.05	1.54
UBE2Q1	ubiquitin-conjugating enzyme E2Q family member 1	chr1:152790165	11	331	4	96.22	90.01	0.10	1.54
CYB5R2	cytochrome b5 reductase 2	chr11:7643432	8	825	11	89.04	82.27	0.11	1.54
DCAF12	DDB1 and CUL4 associated factor 12	chr9:34115481	1	870	8	90.91	97.36	-0.10	1.54
MCM3	minichromosome maintenance complex component 3	chr6:52248912	9	112	3	90.01	94.63	-0.07	1.53
DVL2	dishevelled, dsh homolog 2 (Drosophila)	chr17:7077980	1	131	1	93.10	92.59	0.01	1.53
MACROD1	MACRO domain containing 1	chr11:63523172	8	206	1	88.90	98.00	-0.14	1.53
FNDC4	fibronectin type III domain containing 4	chr2:27570543	3	178	3	90.94	84.21	0.11	1.53
CDC73	cell division cycle 73, Paf1/RNA polymerase II complex component, homolog (S. cerevisiae)	chr1:191386209	9	75	1	92.86	85.67	0.12	1.53
TTC37	tetratricopeptide repeat domain 37	chr5:94881792	20	383	4	90.36	75.39	0.26	1.52
C11orf10	chromosome 11 open reading frame 10	chr11:61314424	2	116	4	97.10	71.31	0.45	1.52
RAB6B	RAB6B, member RAS oncogene family	chr3:135040318	5	721	15	90.01	93.10	-0.05	1.52
CELA1	chymotrypsin-like elastase family, member 1	chr12:50019151	6	759	10	99.13	89.37	0.15	1.52
LRP6	low density lipoprotein receptor-related protein 6	chr12:12202776	12	253	5	89.46	90.83	-0.02	1.52
C1orf163	chromosome 1 open reading frame 163	chr1:52936170	1	310	3	91.83	91.43	0.01	1.52
HK2	hexokinase 2	chr2:74948654	3	236	3	90.14	91.30	-0.02	1.52
VAMP8	vesicle-associated membrane protein 8 (endobrevin)	chr2:85659182	1	900	15	88.72	91.00	-0.04	1.52
ITGA11	integrin, alpha 11	chr15:66400382	19	427	7	91.46	90.68	0.01	1.52
MRPL38	mitochondrial ribosomal protein L38	chr17:71407028	7	163	4	90.94	84.83	0.10	1.52
LPIN2	lipin 2	chr18:2927325	7	364	5	88.65	88.39	0.00	1.52
CDR2L	cerebellar degeneration-related protein 2-like	chr17:70510395	4	478	9	89.25	91.01	-0.03	1.52
SLC25A21	solute carrier family 25 (mitochondrial oxodicarboxylate carrier), member 21	chr14:36273265	4	197	5	89.77	96.56	-0.11	1.52

Gene symbol	Gene name	Location of latent 5’SS	Intron number	Position of latent 5’SS[a]	Number of stop codons[b]	Latent score[c]	Authentic score[c]	Log score ratio[d]	Fold change
MCM3	minichromosome maintenance complex component 3	chr6:52245254	11	732	22	91.36	88.61	0.04	1.52
CAP2	CAP, adenylate cyclase-associated protein, 2 (yeast)	chr6:17648192	8	525	4	89.46	95.23	-0.09	1.51
ILF2	interleukin enhancer binding factor 2, 45kDa	chr1:151909377	1	640	11	90.14	97.82	-0.12	1.51
GPR143	G protein-coupled receptor 143	chrX:9692850	1	757	8	89.53	89.59	0.00	1.51
XDH	xanthine dehydrogenase	chr2:31415345	34	513	11	90.01	82.46	0.13	1.51
COL4A2	collagen, type IV, alpha 2	chr13:109909915	22	634	8	90.46	96.22	-0.09	1.51
DNAI1	dynein, axonemal, intermediate chain 1	chr9:34480215	6	94	1	95.23	78.49	0.28	1.51
JAG1	jagged 1	chr20:10580100	8	128	1	93.00	92.91	0.00	1.51
MAP7	microtubule-associated protein 7	chr6:136739908	7	677	14	89.04	89.77	-0.01	1.51
ITGA5	integrin, alpha 5 (fibronectin receptor, alpha polypeptide)	chr12:53091305	2	579	6	94.63	91.84	0.04	1.51
MED25	mediator complex subunit 25	chr19:55023757	4	142	1	93.20	88.51	0.07	1.50
KAT2B	K(lysine) acetyltransferase 2B	chr3:20117279	4	830	12	88.61	89.77	-0.02	1.50
NPRL3	nitrogen permease regulator-like 3 (S. cerevisiae)	chr16:82948	8	268	2	94.18	82.02	0.20	1.50
SP1	Sp1 transcription factor	chr12:52090123	5	512	8	92.23	96.83	-0.07	1.50

[a]Number of nt downstream from the authentic 5’SS.
[b]Number of in frame stop codons within the latent exon
[c]See Methods
[d]Latent score divided by authentic score

Supplementary Table 3. List of latent 5’SSs activated in MCF-7 breast cancer cells

Gene symbol	Gene name	Location of latent 5'SS	Intron number	Position of latent 5'SS[a]	Number of stop codons[b]	Latent score[c]	Authentic score[c]	Log score ratio[d]	Up in 1a[e]	Up in 1b[e]	Up in 1c[e]
ABCC2	ATP-binding cassette, sub-family C (CFTR/MRP), member 2	chr10:101532890	1	267	4	89.77	87.72	0.03	+	+	+
ABCC2	ATP-binding cassette, sub-family C (CFTR/MRP), member 2	chr10:101581339	21	742	12	90.46	88.24	0.04	+	+	+
ABCC2	ATP-binding cassette, sub-family C (CFTR/MRP), member 2	chr10:101596254	29	726	6	91.67	96.83	-0.08	+	+	+
ACTR5	ARP5 actin-related protein 5 homolog (yeast)	chr20:36812447	2	152	4	91.36	96.95	-0.09	+	+	+
ADA	adenosine deaminase	chr20:42685918	6	338	4	89.46	94.41	-0.08	+	+	+
ADCY9	adenylate cyclase 9	chr16:3982492	4	915	5	88.63	88.32	0	+	+	+
AKR1B1	aldo-keto reductase family 1, member B1 (aldose reductase)	chr7:133793873	1	415	1	90.29	94.14	-0.06	+	+	+
ALG14	asparagine-linked glycosylation 14 homolog (S. cerevisiae)	chr1:95310401	1	505	6	89.06	95.74	-0.1	+	+	+
ALK	anaplastic lymphoma receptor tyrosine kinase	chr2:29303130	18	161	2	93.27	89.14	0.07	+	+	+
APEH	N-acylaminoacyl-peptide hydrolase	chr3:49687994	3	249	4	91.5	86.5	0.08	+	+	+
APPBP2	amyloid beta precursor protein (cytoplasmic tail) binding protein 2	chr17:55893301	8	651	13	88.65	94.07	-0.09	+	+	+
ARHGAP18	Rho GTPase activating protein 18	chr6:129973715	8	655	10	90.97	88.39	0.04	+	+	+
ARHGAP18	Rho GTPase activating protein 18	chr6:129968361	10	353	9	88.64	87.96	0.01	+	+	+
ARHGEF12	Rho guanine nucleotide exchange factor (GEF) 12	chr11:119825518	21	386	7	90.33	86.98	0.05	+	+	+
ARHGEF37	Rho guanine nucleotide exchange factor (GEF) 37	chr5:148980813	8	594	12	96.36	81.59	0.24	+	+	+
ARL2BP	ADP-ribosylation factor-like 2 binding protein	chr16:55837221	1	404	5	92.3	81.59	0.18	+	+	+
ASF1B	ASF1 anti-silencing function 1 homolog B (S. cerevisiae)	chr19:14093048	3	295	4	89.46	92.64	-0.05	+	+	+
ATP5F1	ATP synthase, H+ transporting, mitochondrial Fo complex, subunit B1	chr1:111799488	3	988	21	89.77	96.83	-0.11	+	+	+
ATP6V1D	ATPase, H+ transporting, lysosomal 34kDa, V1 subunit D	chr14:66879300	7	541	13	92.83	93.3	-0.01	+	+	+
ATPAF2	ATP synthase mitochondrial F1 complex assembly factor 2	chr17:17882304	1	615	8	93.3	97.23	-0.06	+	+	+
AVEN	apoptosis, caspase activation inhibitor	chr15:31946537	5	450	7	89.82	91.17	-0.02	+	+	+
BAG2	BCL2-associated athanogene 2	chr6:57155253	2	356	4	90.99	90.96	0	+	+	+
BAIAP2L1	BAI1-associated protein 2-like 1	chr7:97761085	13	236	3	89.25	92.98	-0.06	+	+	+
BAIAP2L2	BAI1-associated protein 2-like 2	chr22:36812518	11	558	7	91.49	86.36	0.08	+	+	+
BLMH	bleomycin hydrolase	chr17:25641461	2	993	23	89.63	91.43	-0.03	+	+	+
C18orf45	chromosome 18 open reading frame 45	chr18:19190053	12	502	13	88.65	89.53	-0.01	+	+	+

Supplementary Table 3. List of latent 5'SSs activated in MCF-7 breast cancer cells (continued)

Gene symbol	Gene name	Location of latent 5'SS	Intron number	Position of latent 5'SS[a]	Number of stop codons[b]	Latent score[c]	Authentic score[c]	Log score ratio[d]	Up in 1a[e]	Up in 1b[e]	Up in 1c[e]
C1orf97	chromosome 1 open reading frame 97	chr1:209632207	3	267	4	99.13	92.94	0.09	+	+	+
C20orf11	chromosome 20 open reading frame 11	chr20:61043683	2	267	7	90.73	90.06	0.01	+	+	+
C6orf126	chromosome 6 open reading frame 126	chr6:35852556	1	100	1	92.23	95.69	-0.05	+	+	+
C6orf192	chromosome 6 open reading frame 192	chr6:133141512	7	587	15	91.43	98.87	-0.11	+	+	+
C6orf192	chromosome 6 open reading frame 192	chr6:133136212	9	841	29	91.66	90.69	0.02	+	+	+
C9orf50	chromosome 9 open reading frame 50	chr9:131415172	6	34	1	92.13	92.11	0	+	+	+
CA13	carbonic anhydrase XIII	chr8:86346017	1	672	12	91.46	91.43	0	+	+	+
CA2	carbonic anhydrase II	chr8:86564319	1	724	4	91.5	93.73	-0.03	+	+	+
CA3	carbonic anhydrase III, muscle specific	chr8:86546098	6	321	7	93.53	96.83	-0.05	+	+	+
CACNA1S	calcium channel, voltage-dependent, L type, alpha 1S subunit	chr1:199287329	32	978	9	89.3	78.64	0.18	+	+	+
CALY	calcyon neuron-specific vesicular protein	chr10:134990844	3	554	3	94.43	84.55	0.16	+	+	+
CARD11	caspase recruitment domain family, member 11	chr7:2929563	15	829	3	88.94	82.93	0.1	+	+	+
CARS2	cysteinyl-tRNA synthetase 2, mitochondrial (putative)	chr13:110096662	11	786	5	89.06	95.05	-0.09	+	+	+
CARS2	cysteinyl-tRNA synthetase 2, mitochondrial (putative)	chr13:110092333	14	329	1	88.65	98	-0.14	+	+	+
CCDC82	coiled-coil domain containing 82	chr11:95745982	6	204	4	98.87	94.17	0.07	+	+	+
CCNG1	cyclin G1	chr5:162801906	5	180	6	90.5	92.94	-0.04	+	+	+
CD244	CD244 molecule, natural killer cell receptor 2B4	chr1:159077248	3	375	9	90.05	94.63	-0.07	+	+	+
CDC40	cell division cycle 40 homolog (S. cerevisiae)	chr6:110640757	7	590	15	88.72	96.56	-0.12	+	+	+
CHAF1B	chromatin assembly factor 1, subunit B (p60)	chr21:36703940	10	308	3	90.43	84	0.11	+	+	+
CLCA2	chloride channel accessory 2	chr1:86686736	11	688	9	90.24	89.46	0.01	+	+	+
CLMP	CXADR-like membrane protein	chr11:122450499	6	120	6	90.58	81.58	0.15	+	+	+
CLPX	ClpX caseinolytic peptidase X homolog (E. coli)	chr15:63234889	10	193	2	90.97	98.87	-0.12	+	+	+
CLSPN	claspin	chr1:35975887	22	292	7	90.33	91	-0.01	+	+	+
CLYBL	citrate lyase beta like	chr13:99315603	5	438	5	88.84	78.26	0.18	+	+	+
CNIH4	cornichon homolog 4 (Drosophila)	chr1:222620599	3	284	8	88.65	90.47	-0.03	+	+	+
COL17A1	collagen, type XVII, alpha 1	chr10:105823700	6	231	4	91.87	84.98	0.11	+	+	+
COL17A1	collagen, type XVII, alpha 1	chr10:105812697	11	797	20	90.97	91.4	-0.01	+	+	+
COL17A1	collagen, type XVII, alpha 1	chr10:105786889	46	494	9	89.93	84.19	0.1	+	+	+
COL4A1	collagen, type IV, alpha 1	chr13:109661452	8	542	4	95.23	85.54	0.15	+	+	+
COL4A1	collagen, type IV, alpha 1	chr13:109657382	13	368	5	92.04	90.17	0.03	+	+	+
COL4A2	collagen, type IV, alpha 2	chr13:109900628	19	492	7	91.67	87.88	0.06	+	+	+
COL4A2	collagen, type IV, alpha 2	chr13:109913054	24	323	6	91.41	85.05	0.1	+	+	+
CPD	carboxypeptidase D	chr17:25806900	15	287	7	90.91	94.07	-0.05	+	+	+

Supplementary Table 3. List of latent 5'SSs activated in MCF-7 breast cancer cells (continued)

Gene symbol	Gene name	Location of latent 5'SS	Intron number	Position of latent 5'SS[a]	Number of stop codons[b]	Latent score[c]	Authentic score[c]	Log score ratio[d]	Up in 1a[e]	Up in 1b[e]	Up in 1c[e]
CPS1	carbamoyl-phosphate synthase 1, mitochondrial	chr2:211146689	2	314	9	90.12	93.76	-0.06	+	+	+
CPS1	carbamoyl-phosphate synthase 1, mitochondrial	chr2:211213960	24	933	23	91.66	80.68	0.18	+	+	+
CPT2	carnitine palmitoyltransferase 2	chr1:53440827	3	139	5	90.36	91.04	-0.01	+	+	+
CTC1	CTS telomere maintenance complex component 1	chr17:8081927	4	146	4	89.64	91.83	-0.03	+	+	+
CTSZ	cathepsin Z	chr20:57014379	2	392	8	88.72	94.89	-0.1	+	+	+
CUL5	cullin 5	chr11:107450364	10	931	19	89.19	85.48	0.06	+	+	+
CUTC	cutC copper transporter homolog (E. coli)	chr10:101482304	1	149	1	90.29	87.23	0.05	+	+	+
DARS	aspartyl-tRNA synthetase	chr2:136387580	12	928	25	90.96	91.37	-0.01	+	+	+
DCHS1	dachsous 1 (Drosophila)	chr11:6609235	7	187	1	94.73	94.07	0.01	+	+	+
DERA	deoxyribose-phosphate aldolase (putative)	chr12:16001791	2	558	16	89.27	98.23	-0.14	+	+	+
DHCR24	24-dehydrocholesterol reductase	chr1:55103265	6	298	3	91.43	97.36	-0.09	+	+	+
DHX8	DEAH (Asp-Glu-Ala-His) box polypeptide 8	chr17:38938069	12	351	7	90.97	96.36	-0.08	+	+	+
DHX8	DEAH (Asp-Glu-Ala-His) box polypeptide 8	chr17:38955309	22	190	4	89.94	89.45	0.01	+	+	+
DIEXF	digestive organ expansion factor homolog (zebrafish)	chr1:208084130	11	467	5	89.59	89.64	0	+	+	+
DLAT	dihydrolipoamide S-acetyltransferase	chr11:111437963	13	856	15	92.96	94.63	-0.03	+	+	+
DNAJC17	DnaJ (Hsp40) homolog, subfamily C, member 17	chr15:38854651	7	391	4	90.94	95.69	-0.07	+	+	+
DOCK1	dedicator of cytokinesis 1	chr10:129128328	49	850	11	92.66	84.53	0.13	+	+	+
DTX1	deltex homolog 1 (Drosophila)	chr12:112018457	8	856	15	89.46	93.27	-0.06	+	+	+
DYNC1LI1	dynein, cytoplasmic 1, light intermediate chain 1	chr3:32550177	7	829	17	89.46	87.16	0.04	+	+	+
EFHD2	EF-hand domain family, member D2	chr1:15627176	3	810	9	90.54	96.22	-0.09	+	+	+
EFTUD2	elongation factor Tu GTP binding domain containing 2	chr17:40313151	8	296	3	98	92.51	0.08	+	+	+
EIF3M	eukaryotic translation initiation factor 3, subunit M	chr11:32567059	3	206	5	89.49	84.68	0.08	+	+	+
EPB49	erythrocyte membrane protein band 4.9 (dematin)	chr8:21994199	12	284	3	91.81	42.29	1.12	+	+	+
EPHX4	epoxide hydrolase 4	chr1:92281997	3	873	22	91.46	90.88	0.01	+	+	+
ETFDH	electron-transferring-flavoprotein dehydrogenase	chr4:159823777	3	752	18	90.43	84.88	0.09	+	+	+
EVC	Ellis van Creveld syndrome	chr4:5863508	20	431	2	91.36	91.3	0	+	+	+
EVI5	ecotropic viral integration site 5	chr1:92845569	15	156	5	89.1	91.41	-0.04	+	+	+
EXOSC1	exosome component 1	chr10:99187077	6	369	7	91.5	77.12	0.25	+	+	+
EXOSC5	exosome component 5	chr19:46587260	4	249	5	92.23	94.14	-0.03	+	+	+
FAF1	Fas (TNFRSF6) associated factor 1	chr1:50982207	5	736	22	93.26	85.31	0.13	+	+	+
FGD1	FYVE, RhoGEF and PH domain containing 1	chrX:54490201	17	267	3	88.59	91.77	-0.05	+	+	+
FLOT2	flotillin 2	chr17:24234110	4	141	2	91.36	88.7	0.04	+	+	+
FLOT2	flotillin 2	chr17:24232560	8	397	4	89.44	88.14	0.02	+	+	+

Supplementary Table 3. List of latent 5'SSs activated in MCF-7 breast cancer cells (continued)

Gene symbol	Gene name	Location of latent 5'SS	Intron number	Position of latent 5'SS[a]	Number of stop codons[b]	Latent score[c]	Authentic score[c]	Log score ratio[d]	Up in 1a[e]	Up in 1b[e]	Up in 1c[e]
GALNT8	UDP-N-acetyl-alpha-D-galactosamine:polypeptide N-acetylgalactosaminyltransferase 8 (GalNAc-T8)	chr12:4740677	7	108	1	92.86	99.13	-0.09	+	+	+
GAPDHS	glyceraldehyde-3-phosphate dehydrogenase, spermatogenic	chr19:40725618	6	269	4	91.81	87.88	0.06	+	+	+
GCLM	glutamate-cysteine ligase, modifier subunit	chr1:94141910	2	756	20	90.42	94.63	-0.07	+	+	+
GLB1L2	galactosidase, beta 1-like 2	chr11:133747699	15	766	7	100	93.27	0.1	+	+	+
GLB1L2	galactosidase, beta 1-like 2	chr11:133749045	16	134	1	95.18	92.23	0.05	+	+	+
GMDS	GDP-mannose 4,6-dehydratase	chr6:1905405	5	601	9	89.01	90.53	-0.02	+	+	+
GNB4	guanine nucleotide binding protein (G protein), beta polypeptide 4	chr3:180614550	6	816	23	90.69	96.83	-0.09	+	+	+
GNL2	guanine nucleotide binding protein-like 2 (nucleolar)	chr1:37831094	2	855	22	89.01	96.83	-0.12	+	+	+
GNL2	guanine nucleotide binding protein-like 2 (nucleolar)	chr1:37814283	9	332	3	94.14	93	0.02	+	+	+
GOLIM4	golgi integral membrane protein 4	chr3:169232710	9	291	4	99.13	89.12	0.15	+	+	+
GPC1	glypican 1	chr2:241052309	4	708	8	95.52	88.19	0.12	+	+	+
GRAMD3	GRAM domain containing 3	chr5:125847771	9	607	11	90.68	80.47	0.17	+	+	+
GYS1	glycogen synthase 1 (muscle)	chr19:54182032	3	230	3	89.14	90.8	-0.03	+	+	+
HELZ	helicase with zinc finger	chr17:62587054	16	420	9	92.86	91.96	0.01	+	+	+
HHLA1	HERV-H LTR-associating 1	chr8:133152640	5	128	2	97.1	90.05	0.11	+	+	+
HOMER1	homer homolog 1 (Drosophila)	chr5:78843380	1	968	16	88.8	91.81	-0.05	+	+	+
HSPA4L	heat shock 70kDa protein 4-like	chr4:128961746	14	577	15	90.27	91.89	-0.03	+	+	+
INTS10	integrator complex subunit 10	chr8:19726048	7	214	5	89.93	83.66	0.1	+	+	+
ITCH	itchy E3 ubiquitin protein ligase homolog (mouse)	chr20:32494743	10	975	22	90.46	91.36	-0.01	+	+	+
ITGA2	integrin, alpha 2 (CD49B, alpha 2 subunit of VLA-2 receptor)	chr5:52377449	4	771	20	89.67	84.62	0.08	+	+	+
ITGA5	integrin, alpha 5 (fibronectin receptor, alpha polypeptide)	chr12:53084550	13	203	3	88.78	94.43	-0.09	+	+	+
JAM3	junctional adhesion molecule 3	chr11:133516204	3	330	6	94.73	85.93	0.14	+	+	+
KCTD20	potassium channel tetramerisation domain containing 20	chr6:36551469	3	653	15	91.81	91.09	0.01	+	+	+
KIAA0368	KIAA0368	chr9:113194259	29	609	3	91.36	91.17	0	+	+	+
KLHL22	kelch-like 22 (Drosophila)	chr22:19130209	6	520	4	91.43	99.13	-0.12	+	+	+
KRT4	keratin 4	chr12:51488953	4	480	4	91.4	90.92	0.01	+	+	+
KRT7	keratin 7	chr12:50914398	1	728	5	89.71	95.69	-0.09	+	+	+
KRTDAP	keratinocyte differentiation-associated protein	chr19:40672531	1	566	5	89.59	94.65	-0.08	+	+	+
L2HGDH	L-2-hydroxyglutarate dehydrogenase	chr14:49829674	4	908	20	94.07	94.63	-0.01	+	+	+
LECT2	leukocyte cell-derived chemotaxin 2	chr5:135314550	3	260	6	89.06	95.05	-0.09	+	+	+
LMNB2	lamin B2	chr19:2382031	11	514	3	90.46	92.51	-0.03	+	+	+
LPCAT4	lysophosphatidylcholine acyltransferase 4	chr15:32443994	3	506	5	90.39	94.18	-0.06	+	+	+

Supplementary Table 3. List of latent 5'SSs activated in MCF-7 breast cancer cells (continued)

Gene symbol	Gene name	Location of latent 5'SS	Intron number	Position of latent 5'SS[a]	Number of stop codons[b]	Latent score[c]	Authentic score[c]	Log score ratio[d]	Up in 1a[e]	Up in 1b[e]	Up in 1c[e]
LPXN	leupaxin	chr11:58074835	5	278	7	95.05	95.23	0	+	+	+
LRFN3	leucine rich repeat and fibronectin type III domain containing 3	chr19:41124018	2	437	8	89.04	84.04	0.08	+	+	+
LRP6	low density lipoprotein receptor-related protein 6	chr12:12287854	2	608	10	90.43	91.36	-0.01	+	+	+
LRRC47	leucine rich repeat containing 47	chr1:3688145	5	939	10	90.43	92.37	-0.03	+	+	+
MCOLN2	mucolipin 2	chr1:85175383	11	642	15	93.3	92.07	0.02	+	+	+
MET	met proto-oncogene (hepatocyte growth factor receptor)	chr7:116197942	12	862	15	90.68	89.46	0.02	+	+	+
MFSD8	major facilitator superfamily domain containing 8	chr4:129072634	9	955	25	89.06	89.05	0	+	+	+
MIS18BP1	MIS18 binding protein 1	chr14:44748782	14	550	13	89.64	88.14	0.02	+	+	+
MOCOS	molybdenum cofactor sulfurase	chr18:32029587	2	281	1	92.26	84.94	0.12	+	+	+
MPHOSPH6	M-phase phosphoprotein 6	chr16:80760524	1	706	8	93.76	90.36	0.05	+	+	+
MRPS15	mitochondrial ribosomal protein S15	chr1:36701405	2	588	18	90.97	92.51	-0.02	+	+	+
MRTO4	mRNA turnover 4 homolog (S. cerevisiae)	chr1:19456459	4	234	2	92.56	100	-0.11	+	+	+
MST1R	macrophage stimulating 1 receptor (c-met-related tyrosine kinase)	chr3:49906922	14	681	5	88.65	90.29	-0.03	+	+	+
MTFMT	mitochondrial methionyl-tRNA formyltransferase	chr15:63095555	6	271	9	92.96	87.08	0.09	+	+	+
NAA15	N(alpha)-acetyltransferase 15, NatA auxiliary subunit	chr4:140475331	2	470	13	90.53	97.82	-0.11	+	+	+
NAA15	N(alpha)-acetyltransferase 15, NatA auxiliary subunit	chr4:140501984	13	753	9	90.68	87.54	0.05	+	+	+
NAA15	N(alpha)-acetyltransferase 15, NatA auxiliary subunit	chr4:140519713	17	256	6	91.55	89.37	0.03	+	+	+
NCAPG	non-SMC condensin I complex, subunit G	chr4:17452231	19	818	15	98.23	83.16	0.24	+	+	+
NMNAT1	nicotinamide nucleotide adenylyltransferase 1	chr1:9955368	2	536	7	89.46	92.51	-0.05	+	+	+
NMU	neuromedin U	chr4:56165707	7	491	9	94.41	82.07	0.2	+	+	+
NPC1	Niemann-Pick disease, type C1	chr18:19378099	13	206	4	89.01	84.31	0.08	+	+	+
NPC1	Niemann-Pick disease, type C1	chr18:19373461	18	311	5	91.4	79.62	0.2	+	+	+
NUP133	nucleoporin 133kDa	chr1:227689393	10	442	3	92.98	83.77	0.15	+	+	+
NUP133	nucleoporin 133kDa	chr1:227668140	16	863	14	89.88	82.77	0.12	+	+	+
NUP133	nucleoporin 133kDa	chr1:227666691	18	302	8	92.66	81.09	0.19	+	+	+
NUP133	nucleoporin 133kDa	chr1:227650582	24	913	14	88.65	88.51	0	+	+	+
NUP35	nucleoporin 35kDa	chr2:183731529	7	146	2	90.83	91.78	-0.02	+	+	+
PARP1	poly (ADP-ribose) polymerase 1	chr1:224622077	16	445	3	89.46	85.73	0.06	+	+	+
PARP1	poly (ADP-ribose) polymerase 1	chr1:224621072	17	731	9	91.86	88.32	0.06	+	+	+
PBK	PDZ binding kinase	chr8:27724008	7	385	6	91.46	90.17	0.02	+	+	+
PCCA	propionyl CoA carboxylase, alpha polypeptide	chr13:99606167	5	821	14	88.64	84.24	0.07	+	+	+
PDE6A	phosphodiesterase 6A, cGMP-specific, rod, alpha	chr5:149255711	12	400	8	92.94	95.18	-0.03	+	+	+
PEX3	peroxisomal biogenesis factor 3	chr6:143838422	9	737	16	91	92.94	-0.03	+	+	+

Supplementary Table 3. List of latent 5'SSs activated in MCF-7 breast cancer cells (continued)

Gene symbol	Gene name	Location of latent 5'SS	Intron number	Position of latent 5'SS[a]	Number of stop codons[b]	Latent score[c]	Authentic score[c]	Log score ratio[d]	Up in 1a[e]	Up in 1b[e]	Up in 1c[e]
PEX6	peroxisomal biogenesis factor 6	chr6:43044359	6	230	4	90.33	96.22	-0.09	+	+	+
PEX6	peroxisomal biogenesis factor 6	chr6:43042846	8	237	1	91.43	85.6	0.1	+	+	+
PFDN2	prefoldin subunit 2	chr1:159337462	3	999	24	90.96	96.83	-0.09	+	+	+
PGM1	phosphoglucomutase 1	chr1:63868562	3	216	2	88.67	80.87	0.13	+	+	+
PGM1	phosphoglucomutase 1	chr1:63890513	9	403	4	91.55	85.05	0.11	+	+	+
PI4K2B	phosphatidylinositol 4-kinase type 2 beta	chr4:24870711	5	802	13	91.83	84.13	0.13	+	+	+
PLK3	polo-like kinase 3	chr1:45041470	7	59	2	88.59	84.34	0.07	+	+	+
PNPT1	polyribonucleotide nucleotidyltransferase 1	chr2:55741980	14	614	15	92.23	91.78	0.01	+	+	+
POLE2	polymerase (DNA directed), epsilon 2 (p59 subunit)	chr14:49201357	8	217	3	91.4	43.84	1.06	+	+	+
POLR2F	polymerase (RNA) II (DNA directed) polypeptide F	chr22:36683136	2	342	7	95.52	89.22	0.1	+	+	+
POLR2H	polymerase (RNA) II (DNA directed) polypeptide H	chr3:185566053	3	346	7	89.46	97.36	-0.12	+	+	+
PPIH	peptidylprolyl isomerase H (cyclophilin H)	chr1:42904466	7	142	3	90.46	90.91	-0.01	+	+	+
PPM1D	protein phosphatase, Mg2+/Mn2+ dependent, 1D	chr17:56056867	2	976	20	89.96	87.28	0.04	+	+	+
PPP1R14C	protein phosphatase 1, regulatory (inhibitor) subunit 14C	chr6:150578636	2	981	18	97.36	97.7	-0.01	+	+	+
PRDX3	peroxiredoxin 3	chr10:120927498	1	758	6	90.5	90.47	0	+	+	+
PRDX3	peroxiredoxin 3	chr10:120922895	4	343	5	88.65	91.17	-0.04	+	+	+
PRKAA2	protein kinase, AMP-activated, alpha 2 catalytic subunit	chr1:56931599	4	837	29	90.46	85.41	0.08	+	+	+
PRPF4B	PRP4 pre-mRNA processing factor 4 homolog B (yeast)	chr6:4004799	14	753	14	92.37	94.63	-0.03	+	+	+
PRSS12	protease, serine, 12 (neurotrypsin, motopsin)	chr4:119453195	7	608	12	91.09	88.72	0.04	+	+	+
PSMD5	proteasome (prosome, macropain) 26S subunit, non-ATPase, 5	chr9:122630775	5	422	12	89.93	93.2	-0.05	+	+	+
PSME4	proteasome (prosome, macropain) activator subunit 4	chr2:54016153	7	574	12	93.2	90.69	0.04	+	+	+
PSME4	proteasome (prosome, macropain) activator subunit 4	chr2:53975028	34	619	10	90.5	95.52	-0.08	+	+	+
PTDSS2	phosphatidylserine synthase 2	chr11:477339	5	267	2	91.43	88.59	0.05	+	+	+
PTPRM	protein tyrosine phosphatase, receptor type, M	chr18:8361108	22	105	3	92.98	98.87	-0.09	+	+	+
RAB38	RAB38, member RAS oncogene family	chr11:87547133	1	865	12	89.14	96.83	-0.12	+	+	+
RAC2	ras-related C3 botulinum toxin substrate 2 (rho family, small GTP binding protein Rac2)	chr22:35958211	3	575	4	88.91	85.5	0.06	+	+	+
RAD21	RAD21 homolog (S. pombe)	chr8:117947543	2	462	13	89.45	92.94	-0.06	+	+	+
RANBP10	RAN binding protein 10	chr16:66335550	4	141	1	91.67	84.11	0.12	+	+	+
RB1	retinoblastoma 1	chr13:47936448	21	477	17	90.91	93.1	-0.03	+	+	+
RCBTB1	regulator of chromosome condensation (RCC1) and BTB (POZ) domain containing protein 1	chr13:49013320	11	492	12	89.25	79.79	0.16	+	+	+

Supplementary Table 3. List of latent 5'SSs activated in MCF-7 breast cancer cells (continued)

Gene symbol	Gene name	Location of latent 5'SS	Intron number	Position of latent 5'SS[a]	Number of stop codons[b]	Latent score[c]	Authentic score[c]	Log score ratio[d]	Up in 1a[e]	Up in 1b[e]	Up in 1c[e]
RDH12	retinol dehydrogenase 12 (all-trans/9-cis/11-cis)	chr14:67262995	4	371	6	90.8	91.36	-0.01	+	+	+
RETSAT	retinol saturase (all-trans-retinol 13,14-reductase)	chr2:85426148	6	460	3	89.94	89.77	0	+	+	+
RINT1	RAD50 interactor 1	chr7:104961057	2	506	8	90.27	89.48	0.01	+	+	+
RNF20	ring finger protein 20	chr9:103349969	9	349	7	88.59	91.37	-0.04	+	+	+
RPUSD2	RNA pseudouridylate synthase domain containing 2	chr15:38651684	2	294	3	89.46	99.13	-0.15	+	+	+
SAMD12	sterile alpha motif domain containing 12	chr8:119661394	2	740	25	88.86	92.13	-0.05	+	+	+
SCNN1B	sodium channel, nonvoltage-gated 1, beta	chr16:23268209	2	478	4	89.46	88.19	0.02	+	+	+
SCPEP1	serine carboxypeptidase 1	chr17:52428266	8	272	8	96.95	89.77	0.11	+	+	+
SHCBP1	SHC SH2-domain binding protein 1	chr16:45194294	8	657	13	89.93	98	-0.12	+	+	+
SIRPB2	signal-regulatory protein beta 2	chr20:1405736	4	246	3	89.96	96.83	-0.11	+	+	+
SLC20A2	solute carrier family 20 (phosphate transporter), member 2	chr8:42412931	8	732	5	90.46	92.23	-0.03	+	+	+
SLC25A12	solute carrier family 25 (mitochondrial carrier, Aralar), member 12	chr2:172378981	10	895	10	90.43	96.95	-0.1	+	+	+
SLC25A12	solute carrier family 25 (mitochondrial carrier, Aralar), member 12	chr2:172374568	12	411	14	89.25	86.16	0.05	+	+	+
SLC25A26	solute carrier family 25, member 26	chr3:66480114	4	593	11	90.27	91.78	-0.02	+	+	+
SLC28A3	solute carrier family 28 (sodium-coupled nucleoside transporter), member 3	chr9:86096548	11	854	8	91.3	92.91	-0.03	+	+	+
SLC7A1	solute carrier family 7 (cationic amino acid transporter, y+ system), member 1	chr13:28988708	11	572	8	88.59	95.69	-0.11	+	+	+
SMARCA5	SWI/SNF related, matrix associated, actin dependent regulator of chromatin, subfamily a, member 5	chr4:144665525	4	456	11	89.06	81.39	0.13	+	+	+
SNAPC3	small nuclear RNA activating complex, polypeptide 3, 50kDa	chr9:15424368	3	735	18	91.55	86	0.09	+	+	+
SNRNP40	small nuclear ribonucleoprotein 40kDa (U5)	chr1:31537201	3	145	3	90.27	99.13	-0.14	+	+	+
SNX9	sorting nexin 9	chr6:158277769	14	710	15	89.91	83.19	0.11	+	+	+
SORL1	sortilin-related receptor, L(DLR class) A repeats containing	chr11:120931402	18	166	1	93.58	91.89	0.03	+	+	+
SORL1	sortilin-related receptor, L(DLR class) A repeats containing	chr11:120987915	39	839	17	91.43	88.92	0.04	+	+	+
SPATS1	spermatogenesis associated, serine-rich 1	chr6:44419570	1	622	13	90.49	63.91	0.5	+	+	+
SRP54	signal recognition particle 54kDa	chr14:34539290	3	685	11	92.23	88.51	0.06	+	+	+
SSRP1	structure specific recognition protein 1	chr11:56854517	10	387	2	93.76	83.78	0.16	+	+	+
STAT5A	signal transducer and activator of transcription 5A	chr17:37710442	12	247	3	88.72	95.23	-0.1	+	+	+
STX8	syntaxin 8	chr17:9221927	7	666	13	93	90.36	0.04	+	+	+

Supplementary Table 3. List of latent 5'SSs activated in MCF-7 breast cancer cells (continued)

Gene symbol	Gene name	Location of latent 5'SS	Intron number	Position of latent 5'SS[a]	Number of stop codons[b]	Latent score[c]	Authentic score[c]	Log score ratio[d]	Up in 1a[e]	Up in 1b[e]	Up in 1c[e]
SUPV3L1	suppressor of var1, 3-like 1 (S. cerevisiae)	chr10:70617022	3	711	6	89.01	86.8	0.04	+	+	+
TBC1D8	TBC1 domain family, member 8 (with GRAM domain)	chr2:101021146	5	208	2	96.36	86.47	0.16	+	+	+
TBC1D8	TBC1 domain family, member 8 (with GRAM domain)	chr2:101010767	12	174	3	92.26	87.53	0.08	+	+	+
TFB1M	transcription factor B1, mitochondrial	chr6:155658926	4	852	15	92.33	97.82	-0.08	+	+	+
TFCP2L1	transcription factor CP2-like 1	chr2:121711276	10	392	4	95.18	96.83	-0.02	+	+	+
TFCP2L1	transcription factor CP2-like 1	chr2:121705092	13	779	5	89.59	95.18	-0.09	+	+	+
TIMP3	TIMP metallopeptidase inhibitor 3	chr22:31584514	4	390	6	92.98	96.56	-0.05	+	+	+
TLL1	tolloid-like 1	chr4:167240335	20	207	4	88.9	86.13	0.05	+	+	+
TMED6	transmembrane emp24 protein transport domain containing 6	chr16:67942438	1	506	10	94.6	91.89	0.04	+	+	+
TMEM222	transmembrane protein 222	chr1:27530212	2	331	4	90.5	83.38	0.12	+	+	+
TNC	tenascin C	chr9:116847627	17	882	19	91.43	84.19	0.12	+	+	+
TNC	tenascin C	chr9:116837760	21	437	5	93.76	83.91	0.16	+	+	+
TNFRSF11A	tumor necrosis factor receptor superfamily, member 11a, NFKB activator	chr18:58167043	2	582	14	93.27	84.38	0.14	+	+	+
TNFSF13B	tumor necrosis factor (ligand) superfamily, member 13b	chr13:107737754	3	547	13	89.77	87.92	0.03	+	+	+
TNKS2	tankyrase, TRF1-interacting ankyrin-related ADP-ribose polymerase 2	chr10:93567196	3	231	7	90.94	95.3	-0.07	+	+	+
TNKS2	tankyrase, TRF1-interacting ankyrin-related ADP-ribose polymerase 2	chr10:93592184	16	57	1	89.77	100	-0.16	+	+	+
TOR1B	torsin family 1, member B (torsin B)	chr9:131610363	3	901	14	90.97	89.1	0.03	+	+	+
TPMT	thiopurine S-methyltransferase	chr6:18255312	3	720	17	89.21	85.47	0.06	+	+	+
TPR	translocated promoter region (to activated MET oncogene)	chr1:184586600	20	484	11	91.28	88.51	0.04	+	+	+
TPR	translocated promoter region (to activated MET oncogene)	chr1:184549850	50	533	11	94.63	89.49	0.08	+	+	+
TRMT6	tRNA methyltransferase 6 homolog (S. cerevisiae)	chr20:5878107	1	816	6	92.66	91.78	0.01	+	+	+
TTC27	tetratricopeptide repeat domain 27	chr2:32707661	1	711	8	88.61	96.36	-0.12	+	+	+
TTC37	tetratricopeptide repeat domain 37	chr5:94859743	33	86	3	96.1	92.54	0.05	+	+	+
TULP3	tubby like protein 3	chr12:2911351	6	685	17	90.05	90.17	0	+	+	+
UBA7	ubiquitin-like modifier activating enzyme 7	chr3:49821695	17	131	3	88.91	92.04	-0.05	+	+	+
UBASH3B	ubiquitin associated and SH3 domain containing B	chr11:122173224	9	274	2	89.1	91.36	-0.04	+	+	+
UBE2V2	ubiquitin-conjugating enzyme E2 variant 2	chr8:49118736	2	443	15	96.22	86.03	0.16	+	+	+
UBLCP1	ubiquitin-like domain containing CTD phosphatase 1	chr5:158643817	10	893	27	88.61	98.87	-0.16	+	+	+
UFC1	ubiquitin-fold modifier conjugating enzyme 1	chr1:159391324	1	791	12	95.23	86.11	0.15	+	+	+

Supplementary Table 3. List of latent 5'SSs activated in MCF-7 breast cancer cells (continued)

Gene symbol	Gene name	Location of latent 5'SS	Intron number	Position of latent 5'SS[a]	Number of stop codons[b]	Latent score[c]	Authentic score[c]	Log score ratio[d]	Up in 1a[e]	Up in 1b[e]	Up in 1c[e]
UGCG	UDP-glucose ceramide glucosyltransferase	chr9:113725443	3	392	13	91.02	95.6	-0.07	+	+	+
USP24	ubiquitin specific peptidase 24	chr1:55345254	2	245	2	90.14	82.73	0.12	+	+	+
UTP18	UTP18, small subunit (SSU) processome component, homolog (yeast)	chr17:46720948	11	386	6	91.78	83.45	0.14	+	+	+
UTP20	UTP20, small subunit (SSU) processome component, homolog (yeast)	chr12:100209229	8	433	9	92.23	92.04	0	+	+	+
UTRN	utrophin	chr6:145121013	57	196	6	88.8	92.91	-0.07	+	+	+
VWCE	von Willebrand factor C and EGF domains	chr11:60795488	14	234	1	91.49	91.67	0	+	+	+
WDFY2	WD repeat and FYVE domain containing 2	chr13:51228260	9	665	11	93.2	99.13	-0.09	+	+	+
WDFY2	WD repeat and FYVE domain containing 2	chr13:51230719	11	283	4	98	96.1	0.03	+	+	+
WDR41	WD repeat domain 41	chr5:76766931	12	910	25	90.61	81.95	0.14	+	+	+
WDR66	WD repeat domain 66	chr12:120881684	14	302	5	94.07	85.11	0.14	+	+	+
WDR66	WD repeat domain 66	chr12:120884919	16	469	4	89.19	97.7	-0.13	+	+	+
WNT8B	wingless-type MMTV integration site family, member 8B	chr10:102213351	1	369	13	88.59	95.3	-0.11	+	+	+
WRN	Werner syndrome, RecQ helicase-like	chr8:31036969	3	647	18	91.43	95.6	-0.06	+	+	+
XDH	xanthine dehydrogenase	chr2:31459854	10	270	2	90.46	91.83	-0.02	+	+	+
XDH	xanthine dehydrogenase	chr2:31415345	34	513	11	90.01	82.46	0.13	+	+	+
XDH	xanthine dehydrogenase	chr2:31413204	35	806	22	92.64	86.61	0.1	+	+	+
ZNF426	zinc finger protein 426	chr19:9504223	6	297	5	90.01	98	-0.12	+	+	+
ABCC2	ATP-binding cassette, sub-family C (CFTR/MRP), member 2	chr10:101547767	7	690	10	89.94	87.53	0.04	+	+	
ABCC8	ATP-binding cassette, sub-family C (CFTR/MRP), member 8	chr11:17390350	21	438	10	89.93	90.04	0	+	+	
ACAT2	acetyl-CoA acetyltransferase 2	chr6:160119500	8	199	6	95.05	89.64	0.08	+	+	
ACBD3	acyl-CoA binding domain containing 3	chr1:224408138	6	832	17	94.18	94.14	0	+	+	
ACTR1A	ARP1 actin-related protein 1 homolog A, centractin alpha (yeast)	chr10:104232237	7	520	3	93.76	93.1	0.01	+	+	
AHSA1	AHA1, activator of heat shock 90kDa protein ATPase homolog 1 (yeast)	chr14:77004363	7	84	1	88.65	94.43	-0.09	+	+	
ARID3A	AT rich interactive domain 3A (BRIGHT-like)	chr19:916930	6	851	6	90.39	97.1	-0.1	+	+	
ATP6V1G1	ATPase, H+ transporting, lysosomal 13kDa, V1 subunit G1	chr9:116390777	1	757	10	88.65	90.91	-0.04	+	+	
ATP6V1G1	ATPase, H+ transporting, lysosomal 13kDa, V1 subunit G1	chr9:116395351	2	599	7	89.46	86.19	0.05	+	+	
AVL9	AVL9 homolog (S. cerevisiase)	chr7:32566289	10	689	20	88.61	89.64	-0.02	+	+	
BCAS2	breast carcinoma amplified sequence 2	chr1:114924650	2	792	20	88.7	90.73	-0.03	+	+	
BET1	blocked early in transport 1 homolog (S. cerevisiae)	chr7:93470988	1	457	4	91.41	88.59	0.05	+	+	

Supplementary Table 3. List of latent 5'SSs activated in MCF-7 breast cancer cells (continued)

Gene symbol	Gene name	Location of latent 5'SS	Intron number	Position of latent 5'SS[a]	Number of stop codons[b]	Latent score[c]	Authentic score[c]	Log score ratio[d]	Up in 1a[e]	Up in 1b[e]	Up in 1c[e]
BIRC6	baculoviral IAP repeat containing 6	chr2:32556236	35	82	2	92.23	92.94	-0.01	+	+	
BNIPL	BCL2/adenovirus E1B 19kD interacting protein like	chr1:149276431	1	566	10	90.36	91.36	-0.02	+	+	
C2orf44	chromosome 2 open reading frame 44	chr2:24114001	2	49	1	94.87	92.54	0.04	+	+	
C3orf46	chromosome 3 open reading frame 46	chr3:127865399	2	358	3	90.49	89.46	0.02	+	+	
C3orf58	chromosome 3 open reading frame 58	chr3:145188377	2	1000	21	91.53	91.78	0	+	+	
CA2	carbonic anhydrase II	chr8:86574374	4	478	13	93.2	89.21	0.06	+	+	
CAMSAP1	calmodulin regulated spectrin-associated protein 1	chr9:137848742	14	901	12	91.4	91.79	-0.01	+	+	
CBARA1	calcium binding atopy-related autoantigen 1	chr10:73995438	2	958	17	90.9	96.95	-0.09	+	+	
CCDC113	coiled-coil domain containing 113	chr16:56870898	8	857	8	90.14	94.65	-0.07	+	+	
CCDC18	coiled-coil domain containing 18	chr1:93430456	5	206	6	95.23	90.36	0.08	+	+	
CCNB2	cyclin B2	chr15:57185046	1	264	1	89.74	93.73	-0.06	+	+	
CCNB2	cyclin B2	chr15:57203515	8	398	6	89.19	84.35	0.08	+	+	
CD163L1	CD163 molecule-like 1	chr12:7417915	13	389	7	90.36	85.47	0.08	+	+	
CENPQ	centromere protein Q	chr6:49546382	2	433	6	90.91	88.92	0.03	+	+	
CGA	glycoprotein hormones, alpha polypeptide	chr6:87852563	3	123	1	92.64	91.86	0.01	+	+	
CHAF1A	chromatin assembly factor 1, subunit A (p150)	chr19:4361305	3	550	9	89.46	86.67	0.05	+	+	
CHRNB1	cholinergic receptor, nicotinic, beta 1 (muscle)	chr17:7289891	2	440	3	90.58	92.66	-0.03	+	+	
CHUK	conserved helix-loop-helix ubiquitous kinase	chr10:101939929	20	686	13	91.43	94.17	-0.04	+	+	
CLCN1	chloride channel 1, skeletal muscle	chr7:142724164	1	558	10	90.14	90.77	-0.01	+	+	
COL5A2	collagen, type V, alpha 2	chr2:189635405	29	430	9	89.91	89.81	0	+	+	
CYBB	cytochrome b-245 beta polypeptide	chrX:37554505	12	618	16	91.33	86.74	0.07	+	+	
DDX1	DEAD (Asp-Glu-Ala-Asp) box polypeptide 1	chr2:15685624	20	905	29	89.77	83.06	0.11	+	+	
DDX27	DEAD (Asp-Glu-Ala-Asp) box polypeptide 27	chr20:47293508	19	884	9	89.94	83.87	0.1	+	+	
DHCR24	24-dehydrocholesterol reductase	chr1:55124880	1	269	1	92.23	96.83	-0.07	+	+	
DTWD2	DTW domain containing 2	chr5:118351602	1	285	3	88.96	89.06	0	+	+	
ESF1	ESF1, nucleolar pre-rRNA processing protein, homolog (S. cerevisiae)	chr20:13645507	13	507	7	88.59	87.75	0.01	+	+	
ETV6	ets variant 6	chr12:11897999	4	238	5	90.29	83.06	0.12	+	+	
EXOSC1	exosome component 1	chr10:99190514	4	403	11	92.23	92.97	-0.01	+	+	
EXOSC2	exosome component 2	chr9:132565459	5	858	13	88.65	70.18	0.34	+	+	
EXOSC2	exosome component 2	chr9:132566971	6	829	17	91.3	91.78	-0.01	+	+	
EXTL3	exostoses (multiple)-like 3	chr8:28657516	6	867	10	93.53	96.22	-0.04	+	+	
FAIM3	Fas apoptotic inhibitory molecule 3	chr1:205150798	4	899	19	89.01	95.05	-0.09	+	+	

Supplementary Table 3. List of latent 5'SSs activated in MCF-7 breast cancer cells (continued)

Gene symbol	Gene name	Location of latent 5'SS	Intron number	Position of latent 5'SS[a]	Number of stop codons[b]	Latent score[c]	Authentic score[c]	Log score ratio[d]	Up in 1a[e]	Up in 1b[e]	Up in 1c[e]
FAM3D	family with sequence similarity 3, member D	chr3:58600764	7	129	3	89.32	93.1	-0.06	+	+	
FIBCD1	fibrinogen C domain containing 1	chr9:132803057	1	686	7	90.46	91.83	-0.02	+	+	
FKBP3	FK506 binding protein 3, 25kDa	chr14:44656393	7	587	18	92.83	76.91	0.27	+	+	
GALNTL4	UDP-N-acetyl-alpha-D-galactosamine:polypeptide N-acetylgalactosaminyltransferase-like 4	chr11:11304417	9	791	9	93.27	99.13	-0.09	+	+	
GEMIN5	gem (nuclear organelle) associated protein 5	chr5:154250759	26	234	2	89.93	93.13	-0.05	+	+	
GNL3L	guanine nucleotide binding protein-like 3 (nucleolar)-like	chrX:54582535	3	277	3	89.32	98.23	-0.14	+	+	
GNPAT	glyceronephosphate O-acyltransferase	chr1:229478345	14	479	10	89.46	89.38	0	+	+	
GPR126	G protein-coupled receptor 126	chr6:142665661	1	501	6	89.76	91.55	-0.03	+	+	
GTF2H3	general transcription factor IIH, polypeptide 3, 34kDa	chr12:122696729	3	669	11	88.79	90.17	-0.02	+	+	
HDAC4	histone deacetylase 4	chr2:239667476	22	251	1	90.53	96.83	-0.1	+	+	
HELZ	helicase with zinc finger	chr17:62627592	7	114	2	91.5	96.05	-0.07	+	+	
HGSNAT	heparan-alpha-glucosaminide N-acetyltransferase	chr8:43171574	11	250	5	90.12	86.86	0.05	+	+	
IRAK2	interleukin-1 receptor-associated kinase 2	chr3:10243485	10	369	6	91.43	93.53	-0.03	+	+	
ISCU	iron-sulfur cluster scaffold homolog (E. coli)	chr12:107482749	3	453	8	93.3	92.3	0.02	+	+	
ITCH	itchy E3 ubiquitin protein ligase homolog (mouse)	chr20:32544729	23	667	18	91.83	85.43	0.1	+	+	
KBTBD2	kelch repeat and BTB (POZ) domain containing 2	chr7:32880656	3	472	8	88.65	97.7	-0.14	+	+	
KCTD20	potassium channel tetramerisation domain containing 20	chr6:36558221	6	708	15	89.37	84.29	0.08	+	+	
KIF11	kinesin family member 11	chr10:94371949	10	740	20	89.96	93.3	-0.05	+	+	
KIF26B	kinesin family member 26B	chr1:243929871	14	910	9	90.36	88.65	0.03	+	+	
KRR1	KRR1, small subunit (SSU) processome component, homolog (yeast)	chr12:74185867	5	495	14	90.49	94.18	-0.06	+	+	
LCN2	lipocalin 2	chr9:129955076	5	693	5	92.64	81.39	0.19	+	+	
LMAN1	lectin, mannose-binding, 1	chr18:55150683	11	619	9	90.27	86.4	0.06	+	+	
LOH12CR1	loss of heterozygosity, 12, chromosomal region 1	chr12:12480832	3	847	23	91.66	87.88	0.06	+	+	
LRP2	low density lipoprotein receptor-related protein 2	chr2:169736479	58	291	4	89.18	92.26	-0.05	+	+	
LRP5	low density lipoprotein receptor-related protein 5	chr11:67931448	9	592	13	88.65	84.6	0.07	+	+	
LRP5	low density lipoprotein receptor-related protein 5	chr11:67964447	21	488	4	91.99	93.09	-0.02	+	+	
LRRC47	leucine rich repeat containing 47	chr1:3689882	4	537	6	89.67	92.11	-0.04	+	+	
LRRC50	leucine rich repeat containing 50	chr16:82762160	8	698	13	91.84	88.92	0.05	+	+	
LYRM4	LYR motif containing 4	chr6:5161258	2	591	11	92.23	97.23	-0.08	+	+	
MAP6D1	MAP6 domain containing 1	chr3:185024664	1	964	7	92.37	92.3	0	+	+	

Supplementary Table 3. List of latent 5'SSs activated in MCF-7 breast cancer cells (continued)

Gene symbol	Gene name	Location of latent 5'SS	Intron number	Position of latent 5'SS[a]	Number of stop codons[b]	Latent score[c]	Authentic score[c]	Log score ratio[d]	Up in 1a[e]	Up in 1b[e]	Up in 1c[e]
MPO	myeloperoxidase	chr17:53704411	10	696	10	95.18	97.36	-0.03	+	+	
MRPS9	mitochondrial ribosomal protein S9	chr2:105022034	1	918	9	91.67	98.23	-0.1	+	+	
MTM1	myotubularin 1	chrX:149534439	5	610	11	91.5	89.01	0.04	+	+	
MTMR4	myotubularin related protein 4	chr17:53948461	2	478	9	88.59	93.3	-0.07	+	+	
MYH7B	myosin, heavy chain 7B, cardiac muscle, beta	chr20:33030100	3	541	8	90.46	94.6	-0.06	+	+	
NCAPD2	non-SMC condensin I complex, subunit D2	chr12:6490015	4	456	6	89.86	83.89	0.1	+	+	
NCBP1	nuclear cap binding protein subunit 1, 80kDa	chr9:99472466	22	658	9	96.56	94.07	0.04	+	+	
NDUFAB1	NADH dehydrogenase (ubiquinone) 1, alpha/beta subcomplex, 1, 8kDa	chr16:23503681	3	476	3	96.36	89.14	0.11	+	+	
NDUFAF4	NADH dehydrogenase (ubiquinone) 1 alpha subcomplex, assembly factor 4	chr6:97451639	1	623	6	89.46	82.44	0.12	+	+	
NID1	nidogen 1	chr1:234267322	5	704	5	90.43	90.69	0	+	+	
NKD2	naked cuticle homolog 2 (Drosophila)	chr5:1088361	7	344	20	92.04	93.76	-0.03	+	+	
NOC3L	nucleolar complex associated 3 homolog (S. cerevisiae)	chr10:96101352	8	704	10	90.39	85.48	0.08	+	+	
NOL11	nucleolar protein 11	chr17:63165175	14	243	6	89.46	70.15	0.35	+	+	
NUP88	nucleoporin 88kDa	chr17:5235153	10	451	5	89.46	79.86	0.16	+	+	
PADI4	peptidyl arginine deiminase, type IV	chr1:17538134	5	898	15	89.01	89.64	-0.01	+	+	
PAFAH1B2	platelet-activating factor acetylhydrolase 1b, catalytic subunit 2 (30kDa)	chr11:116540099	5	282	2	90.97	84.98	0.1	+	+	
PDCD11	programmed cell death 11	chr10:105191276	30	680	4	90.43	96.22	-0.09	+	+	
PDHX	pyruvate dehydrogenase complex, component X	chr11:34909738	2	132	2	90.42	96.22	-0.09	+	+	
PEX14	peroxisomal biogenesis factor 14	chr1:10478362	2	398	12	97.7	95.3	0.04	+	+	
PIP4K2A	phosphatidylinositol-5-phosphate 4-kinase, type II, alpha	chr10:22936614	3	247	8	88.91	99.13	-0.16	+	+	
PIPOX	pipecolic acid oxidase	chr17:24406062	5	228	5	88.59	95.69	-0.11	+	+	
PLK2	polo-like kinase 2	chr5:57790469	2	99	2	89.18	92.13	-0.05	+	+	
POLR3A	polymerase (RNA) III (DNA directed) polypeptide A, 155kDa	chr10:79443107	11	306	10	96.1	87.65	0.13	+	+	
PPP1R3C	protein phosphatase 1, regulatory (inhibitor) subunit 3C	chr10:93382367	1	325	6	89.49	95.28	-0.09	+	+	
PPWD1	peptidylprolyl isomerase domain and WD repeat containing 1	chr5:64895525	1	437	8	89.45	86.37	0.05	+	+	
PRPF4B	PRP4 pre-mRNA processing factor 4 homolog B (yeast)	chr6:3967351	1	653	7	89.01	87.54	0.02	+	+	
RAD21	RAD21 homolog (S. pombe)	chr8:117932757	11	610	13	90.36	87.29	0.05	+	+	
RARS	arginyl-tRNA synthetase	chr5:167866956	11	543	17	91.41	92.91	-0.02	+	+	
RNF213	ring finger protein 213	chr17:75979223	42	633	16	92.23	90.12	0.03	+	+	
RPF1	ribosome production factor 1 homolog (S. cerevisiae)	chr1:84718277	1	498	6	90.58	90.7	0	+	+	

Supplementary Table 3. List of latent 5'SSs activated in MCF-7 breast cancer cells (continued)

Gene symbol	Gene name	Location of latent 5'SS	Intron number	Position of latent 5'SS[a]	Number of stop codons[b]	Latent score[c]	Authentic score[c]	Log score ratio[d]	Up in 1a[e]	Up in 1b[e]	Up in 1c[e]
RPF1	ribosome production factor 1 homolog (S. cerevisiae)	chr1:84735415	8	775	15	90.43	92.94	-0.04	+	+	
RRM1	ribonucleotide reductase M1	chr11:4086259	4	919	19	91.43	83.45	0.13	+	+	
SASS6	spindle assembly 6 homolog (C. elegans)	chr1:100360857	3	496	8	92.3	91.09	0.02	+	+	
SCNN1G	sodium channel, nonvoltage-gated 1, gamma	chr16:23116480	6	232	1	95.18	92.54	0.04	+	+	
SCPEP1	serine carboxypeptidase 1	chr17:52431683	10	826	14	91.53	83.78	0.13	+	+	
SCPEP1	serine carboxypeptidase 1	chr17:52434014	11	663	14	88.65	83.87	0.08	+	+	
SLC46A2	solute carrier family 46, member 2	chr9:114687866	3	694	13	88.9	88.11	0.01	+	+	
SMCHD1	structural maintenance of chromosomes flexible hinge domain containing 1	chr18:2734066	3	141	1	88.92	97.82	-0.14	+	+	
SMCHD1	structural maintenance of chromosomes flexible hinge domain containing 1	chr18:2775412	19	794	20	90.8	95.6	-0.07	+	+	
SORCS2	sortilin-related VPS10 domain containing receptor 2	chr4:7766107	15	628	6	89.01	92.11	-0.05	+	+	
SRRM4	serine/arginine repetitive matrix 4	chr12:118039867	4	672	8	90.97	84.13	0.11	+	+	
STK39	serine threonine kinase 39	chr2:168577179	16	210	4	93.27	88.14	0.08	+	+	
STS	steroid sulfatase (microsomal), isozyme S	chrX:7186035	4	407	12	95.23	87.9	0.12	+	+	
TBK1	TANK-binding kinase 1	chr12:63176060	15	239	6	89.46	86.04	0.06	+	+	
TEP1	telomerase-associated protein 1	chr14:19938674	9	308	7	91.37	76.96	0.25	+	+	
TNKS2	tankyrase, TRF1-interacting ankyrin-related ADP-ribose polymerase 2	chr10:93595405	17	641	20	89.74	93.58	-0.06	+	+	
TOR1B	torsin family 1, member B (torsin B)	chr9:131607052	2	615	11	90.96	84.03	0.11	+	+	
TPX2	TPX2, microtubule-associated, homolog (Xenopus laevis)	chr20:29822107	6	173	6	91.53	85.31	0.1	+	+	
TRIM21	tripartite motif containing 21	chr11:4366959	3	500	14	90.46	96.1	-0.09	+	+	
TRUB1	TruB pseudouridine (psi) synthase homolog 1 (E. coli)	chr10:116721133	5	945	18	94.63	87.29	0.12	+	+	
TSNAX	translin-associated factor X	chr1:229745329	4	350	13	89.63	100	-0.16	+	+	
TSR1	TSR1, 20S rRNA accumulation, homolog (S. cerevisiae)	chr17:2183534	6	66	4	90.27	86.03	0.07	+	+	
TSSC1	tumor suppressing subtransferable candidate 1	chr2:3360102	1	323	6	91.37	94.41	-0.05	+	+	
TXLNG	taxilin gamma	chrX:16768386	9	427	9	89.19	94.18	-0.08	+	+	
UAP1	UDP-N-acteylglucosamine pyrophosphorylase 1	chr1:160824456	6	375	11	92.23	85.21	0.11	+	+	
UBA7	ubiquitin-like modifier activating enzyme 7	chr3:49819868	21	304	6	88.86	91.43	-0.04	+	+	
UCK2	uridine-cytidine kinase 2	chr1:164133166	4	974	14	90.42	91.09	-0.01	+	+	
UHRF1BP1	UHRF1 binding protein 1	chr6:34913062	8	960	20	94.18	84.06	0.16	+	+	
WDR60	WD repeat domain 60	chr7:158416352	21	386	10	88.65	97.23	-0.13	+	+	
WDR76	WD repeat domain 76	chr15:41906940	1	359	3	88.65	95.23	-0.1	+	+	
ZNF318	zinc finger protein 318	chr6:43418081	7	310	3	90.96	85.63	0.09	+	+	

Supplementary Table 3. List of latent 5'SSs activated in MCF-7 breast cancer cells (continued)

Gene symbol	Gene name	Location of latent 5'SS	Intron number	Position of latent 5'SS[a]	Number of stop codons[b]	Latent score[c]	Authentic score[c]	Log score ratio[d]	Up in 1a[e]	Up in 1b[e]	Up in 1c[e]
ZNF445	zinc finger protein 445	chr3:44466685	6	257	6	91.64	91.36	0	+	+	
ZNF530	zinc finger protein 530	chr19:62803956	1	643	7	94.63	96.83	-0.03	+	+	
ABCC2	ATP-binding cassette, sub-family C (CFTR/MRP), member 2	chr10:101544431	6	217	2	90.43	92.51	-0.03	+		+
ABCC2	ATP-binding cassette, sub-family C (CFTR/MRP), member 2	chr10:101595012	28	801	13	88.65	94.14	-0.09	+		+
ADAM8	ADAM metallopeptidase domain 8	chr10:134930681	21	164	1	89.1	96.83	-0.12	+		+
AKAP6	A kinase (PRKA) anchor protein 6	chr14:32139456	6	994	15	89.19	92.54	-0.05	+		+
ALDH9A1	aldehyde dehydrogenase 9 family, member A1	chr1:163904406	8	380	9	91.36	82.58	0.15	+		+
ANKRD35	ankyrin repeat domain 35	chr1:144261695	1	984	7	90.46	88.84	0.03	+		+
AOX1	aldehyde oxidase 1	chr2:201230012	27	108	1	88.72	97.23	-0.13	+		+
APPL1	adaptor protein, phosphotyrosine interaction, PH domain and leucine zipper containing 1	chr3:57257190	9	646	17	90.24	90.73	-0.01	+		+
ARFGAP3	ADP-ribosylation factor GTPase activating protein 3	chr22:41523996	15	992	14	89.9	91.4	-0.02	+		+
ARV1	ARV1 homolog (S. cerevisiae)	chr1:229182124	1	477	10	89.14	82.05	0.12	+		+
BFSP2	beaded filament structural protein 2, phakinin	chr3:134651003	3	825	12	90.35	90.33	0	+		+
BTBD8	BTB (POZ) domain containing 8	chr1:92377751	6	177	3	90.22	85.43	0.08	+		+
C10orf27	chromosome 10 open reading frame 27	chr10:72204497	8	453	7	91.99	91.99	0	+		+
C14orf23	chromosome 14 open reading frame 23	chr14:28312989	2	600	10	93.12	81.52	0.19	+		+
C17orf48	chromosome 17 open reading frame 48	chr17:10549654	2	86	2	88.64	90.54	-0.03	+		+
C17orf69	chromosome 17 open reading frame 69	chr17:41074082	1	266	8	91.43	50.89	0.85	+		+
C4orf3	chromosome 4 open reading frame 3	chr4:120440805	1	133	2	90.17	93.3	-0.05	+		+
CD2AP	CD2-associated protein	chr6:47671801	12	81	4	94.07	92.91	0.02	+		+
CERK	ceramide kinase	chr22:45475356	7	840	6	88.65	87.73	0.02	+		+
CHGB	chromogranin B (secretogranin 1)	chr20:5846060	3	496	9	90.22	88.67	0.02	+		+
COL5A1	collagen, type V, alpha 1	chr9:136764378	9	585	11	94.89	93.09	0.03	+		+
COL5A2	collagen, type V, alpha 2	chr2:189625508	40	211	5	90.42	87.29	0.05	+		+
CPS1	carbamoyl-phosphate synthase 1, mitochondrial	chr2:211231936	31	280	5	90.96	88.14	0.05	+		+
CTSH	cathepsin H	chr15:77010424	8	423	4	91.99	88.76	0.05	+		+
CTSZ	cathepsin Z	chr20:57005439	4	613	10	90.46	97.36	-0.11	+		+
CYP2J2	cytochrome P450, family 2, subfamily J, polypeptide 2	chr1:60164691	1	105	1	89.25	87.81	0.02	+		+
DCLK1	doublecortin-like kinase 1	chr13:35259840	17	508	6	88.61	90.17	-0.03	+		+
DCP1A	DCP1 decapping enzyme homolog A (S. cerevisiae)	chr3:53300761	7	377	7	90.17	90.06	0	+		+

Supplementary Table 3. List of latent 5'SSs activated in MCF-7 breast cancer cells (continued)

Gene symbol	Gene name	Location of latent 5'SS	Intron number	Position of latent 5'SS[a]	Number of stop codons[b]	Latent score[c]	Authentic score[c]	Log score ratio[d]	Up in 1a[e]	Up in 1b[e]	Up in 1c[e]
DDX46	DEAD (Asp-Glu-Ala-Asp) box polypeptide 46	chr5:134152850	12	671	17	94.14	93.13	0.02	+		+
DHX38	DEAH (Asp-Glu-Ala-His) box polypeptide 38	chr16:70701783	25	874	14	88.91	97.36	-0.13	+		+
DOCK3	dedicator of cytokinesis 3	chr3:51293595	26	921	16	89.96	91.84	-0.03	+		+
DPM2	dolichyl-phosphate mannosyltransferase polypeptide 2, regulatory subunit	chr9:129738210	3	442	3	88.72	91.36	-0.04	+		+
ENTPD7	ectonucleoside triphosphate diphosphohydrolase 7	chr10:101446614	9	746	22	89.11	86.68	0.04	+		+
EXT1	exostosin 1	chr8:118916644	3	219	1	90.77	98.23	-0.11	+		+
FAM151B	family with sequence similarity 151, member B	chr5:79854057	5	345	8	90.43	94.07	-0.06	+		+
FN3KRP	fructosamine 3 kinase related protein	chr17:78272127	3	600	8	90.49	83.66	0.11	+		+
GGCX	gamma-glutamyl carboxylase	chr2:85632665	10	384	5	89.71	89.93	0	+		+
GNAI3	guanine nucleotide binding protein (G protein), alpha inhibiting activity polypeptide 3	chr1:109927634	5	925	21	90.43	92.91	-0.04	+		+
GSX1	GS homeobox 1	chr13:27265454	1	216	3	93.58	89.06	0.07	+		+
HERC1	hect (homologous to the E6-AP (UBE3A) carboxyl terminus) domain and RCC1 (CHC1)-like domain (RLD) 1	chr15:61807512	16	975	18	97.7	96.95	0.01	+		+
HIRA	HIR histone cell cycle regulation defective homolog A (S. cerevisiae)	chr22:17752969	12	74	1	91.36	95.23	-0.06	+		+
HIVEP2	human immunodeficiency virus type I enhancer binding protein 2	chr6:143127485	7	143	3	92.91	82.77	0.17	+		+
IFI30	interferon, gamma-inducible protein 30	chr19:18146368	1	586	2	89.9	92.37	-0.04	+		+
IFI30	interferon, gamma-inducible protein 30	chr19:18149303	5	202	3	98	90.81	0.11	+		+
IFNGR1	interferon gamma receptor 1	chr6:137562826	6	884	21	92.54	80.96	0.19	+		+
IFT57	intraflagellar transport 57 homolog (Chlamydomonas)	chr3:109419300	3	771	18	89.77	88.64	0.02	+		+
INMT	indolethylamine N-methyltransferase	chr7:30759102	1	658	11	93.76	92.13	0.03	+		+
IRAK2	interleukin-1 receptor-associated kinase 2	chr3:10194862	2	159	4	91.49	85.29	0.1	+		+
JAG1	jagged 1	chr20:10578341	9	553	9	91.5	92.83	-0.02	+		+
KBTBD5	kelch repeat and BTB (POZ) domain containing 5	chr3:42703376	1	111	2	90.99	85.49	0.09	+		+
KDM3A	lysine (K)-specific demethylase 3A	chr2:86551662	11	621	14	92.07	95.18	-0.05	+		+
KIAA0776	KIAA0776	chr6:97080142	4	152	4	91.78	84.28	0.12	+		+
KPNA3	karyopherin alpha 3 (importin alpha 4)	chr13:49182183	11	844	15	88.65	92.91	-0.07	+		+
LACRT	lacritin	chr12:53312957	2	273	6	91.36	86.42	0.08	+		+
LAD1	ladinin 1	chr1:199621636	3	449	2	90.41	83.32	0.12	+		+
LPO	lactoperoxidase	chr17:53680613	4	616	9	90.27	84.91	0.09	+		+
MAPK4	mitogen-activated protein kinase 4	chr18:46507031	5	489	11	90.5	82.79	0.13	+		+

Supplementary Table 3. List of latent 5'SSs activated in MCF-7 breast cancer cells (continued)

Gene symbol	Gene name	Location of latent 5'SS	Intron number	Position of latent 5'SS[a]	Number of stop codons[b]	Latent score[c]	Authentic score[c]	Log score ratio[d]	Up in 1a[e]	Up in 1b[e]	Up in 1c[e]
MLNR	motilin receptor	chr13:48694034	1	660	5	90.94	81.55	0.16	+		+
MTHFD1	methylenetetrahydrofolate dehydrogenase (NADP+ dependent) 1, methenyltetrahydrofolate cyclohydrolase, formyltetrahydrofolate synthetase	chr14:63975771	17	129	1	89.86	84.06	0.1	+		+
MYH13	myosin, heavy chain 13, skeletal muscle	chr17:10167936	23	127	3	88.77	95.69	-0.11	+		+
MYH7B	myosin, heavy chain 7B, cardiac muscle, beta	chr20:33033080	7	557	8	92.23	89.31	0.05	+		+
NDUFS6	NADH dehydrogenase (ubiquinone) Fe-S protein 6, 13kDa (NADH-coenzyme Q reductase)	chr5:1855236	1	574	6	89.06	90.46	-0.02	+		+
NLRP6	NLR family, pyrin domain containing 6	chr11:272590	4	752	3	95.18	93.26	0.03	+		+
NOP58	NOP58 ribonucleoprotein homolog (yeast)	chr2:202855947	4	508	8	91.37	89.64	0.03	+		+
NUBPL	nucleotide binding protein-like	chr14:31385727	9	180	1	90.53	92.83	-0.04	+		+
ORC2	origin recognition complex, subunit 2	chr2:201485638	17	624	16	92.23	96.1	-0.06	+		+
ORM2	orosomucoid 2	chr9:116134974	5	945	17	90.61	89.11	0.02	+		+
OXCT1	3-oxoacid CoA transferase 1	chr5:41841312	9	113	3	90.69	78.17	0.21	+		+
PCSK7	proprotein convertase subtilisin/kexin type 7	chr11:116602644	5	438	3	88.77	92.23	-0.06	+		+
PDE6A	phosphodiesterase 6A, cGMP-specific, rod, alpha	chr5:149242283	17	901	24	89.94	86.29	0.06	+		+
PHOX2A	paired-like homeobox 2a	chr11:71629113	2	680	8	94.89	93.26	0.02	+		+
PIGL	phosphatidylinositol glycan anchor biosynthesis, class L	chr17:16161575	5	825	15	88.81	97.1	-0.13	+		+
PLEKHA6	pleckstrin homology domain containing, family A member 6	chr1:202485335	10	961	10	95.18	86.61	0.14	+		+
PPL	periplakin	chr16:4878548	19	431	5	95.23	95.69	-0.01	+		+
PTPN4	protein tyrosine phosphatase, non-receptor type 4 (megakaryocyte)	chr2:120394927	12	641	15	92.54	95.3	-0.04	+		+
PWP1	PWP1 homolog (S. cerevisiae)	chr12:106623265	11	612	15	95.23	88.14	0.11	+		+
ROCK1	Rho-associated, coiled-coil containing protein kinase 1	chr18:16839476	16	833	18	88.7	87.92	0.01	+		+
ROCK1	Rho-associated, coiled-coil containing protein kinase 1	chr18:16826217	17	572	10	92.54	91.41	0.02	+		+
SEC23A	Sec23 homolog A (S. cerevisiae)	chr14:38605603	10	524	12	88.65	96.1	-0.12	+		+
SERHL2	serine hydrolase-like 2	chr22:41299154	11	676	5	88.63	95.05	-0.1	+		+
SLC25A4	solute carrier family 25 (mitochondrial carrier; adenine nucleotide translocator), member 4	chr4:186304647	3	601	9	88.79	91.28	-0.04	+		+
SLC5A2	solute carrier family 5 (sodium/glucose cotransporter), member 2	chr16:31408822	12	663	8	92.23	89.31	0.05	+		+
SND1	staphylococcal nuclease and tudor domain containing 1	chr7:127123016	3	779	11	89.46	95.69	-0.1	+		+

Supplementary Table 3. List of latent 5'SSs activated in MCF-7 breast cancer cells (continued)

Gene symbol	Gene name	Location of latent 5'SS	Intron number	Position of latent 5'SS[a]	Number of stop codons[b]	Latent score[c]	Authentic score[c]	Log score ratio[d]	Up in 1a[e]	Up in 1b[e]	Up in 1c[e]
SORL1	sortilin-related receptor, L(DLR class) A repeats containing	chr11:120873666	6	699	16	92.64	93.13	-0.01	+		+
SORL1	sortilin-related receptor, L(DLR class) A repeats containing	chr11:120946711	23	523	11	91.55	82.87	0.14	+		+
SORL1	sortilin-related receptor, L(DLR class) A repeats containing	chr11:120965706	29	398	11	90.96	90.79	0	+		+
TCTE1	t-complex-associated-testis-expressed 1	chr6:44362906	2	357	2	89.78	86.67	0.05	+		+
TLL2	tolloid-like 2	chr10:98169804	7	898	9	91.96	82.77	0.15	+		+
TMEM104	transmembrane protein 104	chr17:70298246	5	217	1	90.91	81.55	0.16	+		+
TMEM206	transmembrane protein 206	chr1:210626604	3	251	1	90.33	86.05	0.07	+		+
TMOD2	tropomodulin 2 (neuronal)	chr15:49846441	2	386	11	89.65	88.17	0.02	+		+
TRIM35	tripartite motif containing 35	chr8:27201660	5	935	12	90.43	92.13	-0.03	+		+
UNC80	unc-80 homolog (C. elegans)	chr2:210362770	6	197	5	91.43	88.17	0.05	+		+
UTP15	UTP15, U3 small nucleolar ribonucleoprotein, homolog (S. cerevisiae)	chr5:72899396	2	382	12	94.17	98	-0.06	+		+
ZBTB40	zinc finger and BTB domain containing 40	chr1:22711799	11	586	6	91.36	94.14	-0.04	+		+
ACCS	1-aminocyclopropane-1-carboxylate synthase homolog (Arabidopsis)(non-functional)	chr11:44057336	9	427	4	90.27	95.18	-0.08		+	+
ADCY2	adenylate cyclase 2 (brain)	chr5:7811697	16	999	19	89.37	94.17	-0.08		+	+
AFG3L2	AFG3 ATPase family gene 3-like 2 (S. cerevisiae)	chr18:12333691	14	439	6	90.49	88.77	0.03		+	+
ALKBH7	alkB, alkylation repair homolog 7 (E. coli)	chr19:6325025	1	991	4	93.76	89.92	0.06		+	+
AQR	aquarius homolog (mouse)	chr15:33027536	5	220	6	89.55	90.05	-0.01		+	+
ARFGAP3	ADP-ribosylation factor GTPase activating protein 3	chr22:41534131	13	585	10	88.65	87.61	0.02		+	+
ARHGAP1	Rho GTPase activating protein 1	chr11:46673089	3	696	7	92.66	97.7	-0.08		+	+
AVL9	AVL9 homolog (S. cerevisiase)	chr7:32561380	8	648	12	95.05	90.23	0.08		+	+
BAIAP2L1	BAI1-associated protein 2-like 1	chr7:97787094	5	211	3	91.49	89.05	0.04		+	+
BIRC6	baculoviral IAP repeat containing 6	chr2:32673522	67	892	19	89.49	78.54	0.19		+	+
BLMH	bleomycin hydrolase	chr17:25639850	3	666	12	90.43	91.17	-0.01		+	+
C12orf49	chromosome 12 open reading frame 49	chr12:115644490	2	764	11	88.59	88.7	0		+	+
C14orf166B	chromosome 14 open reading frame 166B	chr14:76403033	12	863	10	88.8	88.72	0		+	+
C16orf80	chromosome 16 open reading frame 80	chr16:56706496	4	177	2	89.49	84.19	0.09		+	+
C1orf163	chromosome 1 open reading frame 163	chr1:52936170	1	310	3	91.83	91.43	0.01		+	+
C1orf63	chromosome 1 open reading frame 63	chr1:25445070	2	451	4	90.17	93.27	-0.05		+	+
C1R	complement component 1, r subcomponent	chr12:7132902	5	127	1	91.36	84.91	0.11		+	+
C3orf22	chromosome 3 open reading frame 22	chr3:127754144	2	690	6	90.46	91.49	-0.02		+	+
C4orf14	chromosome 4 open reading frame 14	chr4:57533503	3	567	8	91.09	76.99	0.24		+	+

Supplementary Table 3. List of latent 5'SSs activated in MCF-7 breast cancer cells (continued)

Gene symbol	Gene name	Location of latent 5'SS	Intron number	Position of latent 5'SS[a]	Number of stop codons[b]	Latent score[c]	Authentic score[c]	Log score ratio[d]	Up in 1a[e]	Up in 1b[e]	Up in 1c[e]
C4orf14	chromosome 4 open reading frame 14	chr4:57526920	5	622	10	92.3	98.23	-0.09		+	+
C5	complement component 5	chr9:122815202	17	769	21	88.65	85.78	0.05		+	+
C9orf41	chromosome 9 open reading frame 41	chr9:76802951	5	382	10	89.82	86.47	0.05		+	+
CAPN7	calpain 7	chr3:15257481	14	118	3	92.23	92.23	0		+	+
CBL	Cas-Br-M (murine) ecotropic retroviral transforming sequence	chr11:118664329	12	464	12	93.09	100	-0.1		+	+
CCDC93	coiled-coil domain containing 93	chr2:118404568	23	514	9	96.36	95.05	0.02		+	+
CCNB1	cyclin B1	chr5:68499065	1	200	1	95.23	91.46	0.06		+	+
CDC37L1	cell division cycle 37 homolog (S. cerevisiae)-like 1	chr9:4688690	5	812	26	91.5	91.78	0		+	+
CDC42BPB	CDC42 binding protein kinase beta (DMPK-like)	chr14:102501472	18	899	19	92.51	90.58	0.03		+	+
CEP120	centrosomal protein 120kDa	chr5:122756224	7	668	19	91.99	99.13	-0.11		+	+
CHD1	chromodomain helicase DNA binding protein 1	chr5:98232619	29	738	16	90.63	89.01	0.03		+	+
CHRAC1	chromatin accessibility complex 1	chr8:141594079	2	304	7	89.04	85.96	0.05		+	+
CHUK	conserved helix-loop-helix ubiquitous kinase	chr10:101949324	16	393	6	93.27	97.23	-0.06		+	+
CLMN	calmin (calponin-like, transmembrane)	chr14:94750767	5	963	16	89.96	97.23	-0.11		+	+
COL4A1	collagen, type IV, alpha 1	chr13:109636536	26	196	2	89.06	97.36	-0.13		+	+
COPA	coatomer protein complex, subunit alpha	chr1:158530351	25	546	6	92.66	89.37	0.05		+	+
CPD	carboxypeptidase D	chr17:25795616	11	502	17	97.7	93.76	0.06		+	+
CPOX	coproporphyrinogen oxidase	chr3:99791994	3	171	5	90.14	92.4	-0.04		+	+
CSRP1	cysteine and glycine-rich protein 1	chr1:199720679	5	354	7	95.18	93.27	0.03		+	+
CTNND2	catenin (cadherin-associated protein), delta 2 (neural plakophilin-related arm-repeat protein)	chr5:11617436	3	619	11	90.42	81.02	0.16		+	+
CTR9	Ctr9, Paf1/RNA polymerase II complex component, homolog (S. cerevisiae)	chr11:10750333	19	595	13	92.3	90.06	0.04		+	+
CTSH	cathepsin H	chr15:77016104	4	610	6	90.39	85.91	0.07		+	+
CTSZ	cathepsin Z	chr20:57009086	3	828	8	92.64	87.6	0.08		+	+
DDOST	dolichyl-diphosphooligosaccharide--protein glycosyltransferase	chr1:20854190	5	329	5	89.93	90.49	-0.01		+	+
DDX1	DEAD (Asp-Glu-Ala-Asp) box polypeptide 1	chr2:15688122	25	438	5	89.04	90.83	-0.03		+	+
DDX51	DEAD (Asp-Glu-Ala-Asp) box polypeptide 51	chr12:131192178	6	169	2	90.94	84.33	0.11		+	+
DDX51	DEAD (Asp-Glu-Ala-Asp) box polypeptide 51	chr12:131191553	8	219	1	88.81	84	0.08		+	+
DRP2	dystrophin related protein 2	chrX:100393505	16	778	27	88.79	93.51	-0.07		+	+
EFTUD2	elongation factor Tu GTP binding domain containing 2	chr17:40300444	13	256	3	89.93	99.13	-0.14		+	+

Supplementary Table 3. List of latent 5'SSs activated in MCF-7 breast cancer cells (continued)

Gene symbol	Gene name	Location of latent 5'SS	Intron number	Position of latent 5'SS[a]	Number of stop codons[b]	Latent score[c]	Authentic score[c]	Log score ratio[d]	Up in 1a[e]	Up in 1b[e]	Up in 1c[e]
EHHADH	enoyl-CoA, hydratase/3-hydroxyacyl CoA dehydrogenase	chr3:186429363	4	550	12	90.39	91.4	-0.02		+	+
EIF2C4	eukaryotic translation initiation factor 2C, 4	chr1:36071234	11	477	10	92.23	90.91	0.02		+	+
EPRS	glutamyl-prolyl-tRNA synthetase	chr1:218245780	15	290	1	89.38	88.79	0.01		+	+
EPRS	glutamyl-prolyl-tRNA synthetase	chr1:218222254	23	482	16	90.77	96.36	-0.09		+	+
EPS8	epidermal growth factor receptor pathway substrate 8	chr12:15701488	12	791	12	88.96	90.53	-0.03		+	+
ERO1LB	ERO1-like beta (S. cerevisiae)	chr1:234451531	14	315	1	88.64	86.61	0.03		+	+
EVC	Ellis van Creveld syndrome	chr4:5809278	11	289	1	96.36	86.68	0.15		+	+
EVC	Ellis van Creveld syndrome	chr4:5861439	18	485	7	95.23	83.91	0.18		+	+
EXOC3	exocyst complex component 3	chr5:519084	12	125	1	91.49	98.23	-0.1		+	+
FAM81B	family with sequence similarity 81, member B	chr5:94790624	6	433	10	91.55	90.69	0.01		+	+
FIG4	FIG4 homolog, SAC1 lipid phosphatase domain containing (S. cerevisiae)	chr6:110172144	10	477	10	92.11	90.17	0.03		+	+
FN3KRP	fructosamine 3 kinase related protein	chr17:78274273	4	223	7	93.27	99.13	-0.09		+	+
GLB1L2	galactosidase, beta 1-like 2	chr11:133745125	11	137	1	91.02	84.06	0.11		+	+
GLI3	GLI family zinc finger 3	chr7:42031068	9	319	3	90.5	92.51	-0.03		+	+
GMDS	GDP-mannose 4,6-dehydratase	chr6:1671181	9	467	6	89.63	91.36	-0.03		+	+
GTPBP1	GTP binding protein 1	chr22:37456437	11	825	8	91.99	96.83	-0.07		+	+
GYS1	glycogen synthase 1 (muscle)	chr19:54185441	2	929	10	89.22	90.5	-0.02		+	+
H2AFY2	H2A histone family, member Y2	chr10:71526340	6	826	13	92.33	93.83	-0.02		+	+
HAUS5	HAUS augmin-like complex, subunit 5	chr19:40798670	6	543	8	95.18	89.93	0.08		+	+
HDDC2	HD domain containing 2	chr6:125660781	3	777	27	89.64	89.22	0.01		+	+
HSD17B11	hydroxysteroid (17-beta) dehydrogenase 11	chr4:88514538	3	336	12	90.54	90.49	0		+	+
HTT	huntingtin	chr4:3176014	43	342	8	91.78	83.31	0.14		+	+
IGFBP7	insulin-like growth factor binding protein 7	chr4:57670387	1	412	4	90.01	90.97	-0.02		+	+
ILF2	interleukin enhancer binding factor 2, 45kDa	chr1:151909377	1	640	11	90.14	97.82	-0.12		+	+
INTS10	integrator complex subunit 10	chr8:19727077	8	315	4	97.36	84.49	0.2		+	+
IPO7	importin 7	chr11:9389205	4	997	20	95.3	84.28	0.18		+	+
IQGAP1	IQ motif containing GTPase activating protein 1	chr15:88784629	6	995	27	91.41	98.23	-0.1		+	+
IRF9	interferon regulatory factor 9	chr14:23703638	6	456	8	93.51	94.14	-0.01		+	+
ITGA2	integrin, alpha 2 (CD49B, alpha 2 subunit of VLA-2 receptor)	chr5:52407605	23	715	16	90.91	94.14	-0.05		+	+
JAGN1	jagunal homolog 1 (Drosophila)	chr3:9907924	1	430	5	89.45	89.12	0.01		+	+
KDM4A	lysine (K)-specific demethylase 4A	chr1:43899465	4	796	17	88.65	96.22	-0.12		+	+
KDM5B	lysine (K)-specific demethylase 5B	chr1:200995695	8	470	3	89.86	89.06	0.01		+	+
KIAA0368	KIAA0368	chr9:113242214	7	258	12	90.5	90.49	0		+	+
KIAA0368	KIAA0368	chr9:113184619	36	692	18	89.93	90.9	-0.02		+	+

Supplementary Table 3. List of latent 5'SSs activated in MCF-7 breast cancer cells (continued)

Gene symbol	Gene name	Location of latent 5'SS	Intron number	Position of latent 5'SS[a]	Number of stop codons[b]	Latent score[c]	Authentic score[c]	Log score ratio[d]	Up in 1a[e]	Up in 1b[e]	Up in 1c[e]
KIAA1199	KIAA1199	chr15:78968653	8	462	11	89.01	96.22	-0.11		+	+
KPNB1	karyopherin (importin) beta 1	chr17:43101467	10	693	11	91.33	88.9	0.04		+	+
KPTN	kaptin (actin binding protein)	chr19:52678811	1	192	4	89.63	90.14	-0.01		+	+
LANCL1	LanC lantibiotic synthetase component C-like 1 (bacterial)	chr2:211048873	2	411	11	92.4	93.76	-0.02		+	+
LCP2	lymphocyte cytosolic protein 2 (SH2 domain containing leukocyte protein of 76kDa)	chr5:169629595	7	705	17	90.27	89.57	0.01		+	+
LCT	lactase	chr2:136297511	4	429	4	89.71	98	-0.13		+	+
LIG1	ligase I, DNA, ATP-dependent	chr19:53317577	23	425	3	89.14	89.82	-0.01		+	+
LPCAT4	lysophosphatidylcholine acyltransferase 4	chr15:32444773	2	278	3	93.12	90.73	0.04		+	+
LPCAT4	lysophosphatidylcholine acyltransferase 4	chr15:32441511	10	177	4	88.9	91.36	-0.04		+	+
LPCAT4	lysophosphatidylcholine acyltransferase 4	chr15:32439904	11	988	20	89.06	90.77	-0.03		+	+
LPXN	leupaxin	chr11:58087379	3	824	17	96.22	85.9	0.16		+	+
LRP5	low density lipoprotein receptor-related protein 5	chr11:67941046	13	476	6	88.79	82.08	0.11		+	+
LRRC40	leucine rich repeat containing 40	chr1:70418521	5	835	18	88.63	84.32	0.07		+	+
LRRC45	leucine rich repeat containing 45	chr17:77581143	14	344	3	90.46	94.65	-0.07		+	+
LTA4H	leukotriene A4 hydrolase	chr12:94920585	18	283	8	91.09	90.14	0.02		+	+
MAN1A2	mannosidase, alpha, class 1A, member 2	chr1:117712928	1	299	5	89.12	84.42	0.08		+	+
MAN2A1	mannosidase, alpha, class 2A, member 1	chr5:109188271	16	835	24	89.77	91.86	-0.03		+	+
MAOB	monoamine oxidase B	chrX:43545432	5	930	20	89.14	84.28	0.08		+	+
MAP3K5	mitogen-activated protein kinase kinase kinase 5	chr6:137001733	13	640	22	89.86	85.91	0.06		+	+
MCM3	minichromosome maintenance complex component 3	chr6:52245254	11	732	22	91.36	88.61	0.04		+	+
METTL4	methyltransferase like 4	chr18:2556169	2	650	13	88.78	83.92	0.08		+	+
MPO	myeloperoxidase	chr17:53709210	7	976	11	90.43	89.1	0.02		+	+
MTDH	metadherin	chr8:98769410	4	402	7	94.43	85.48	0.14		+	+
MYOM2	myomesin (M-protein) 2, 165kDa	chr8:2034601	19	382	3	89.36	94.87	-0.09		+	+
NAT10	N-acetyltransferase 10 (GCN5-related)	chr11:34097395	8	770	10	93.2	99.13	-0.09		+	+
NCAPD2	non-SMC condensin I complex, subunit D2	chr12:6497496	13	111	3	89.19	85.89	0.05		+	+
NCBP1	nuclear cap binding protein subunit 1, 80kDa	chr9:99456790	11	780	14	88.63	68.68	0.37		+	+
NCL	nucleolin	chr2:232036542	1	748	9	89.32	89.11	0		+	+
NDUFA4L2	NADH dehydrogenase (ubiquinone) 1 alpha subcomplex, 4-like 2	chr12:55916316	4	326	1	92.59	86.08	0.11		+	+
NDUFS1	NADH dehydrogenase (ubiquinone) Fe-S protein 1, 75kDa (NADH-coenzyme Q reductase)	chr2:206725826	2	760	10	92.26	92.26	0		+	+
NEIL3	nei endonuclease VIII-like 3 (E. coli)	chr4:178495319	4	851	12	91.86	91.78	0		+	+

Supplementary Table 3. List of latent 5'SSs activated in MCF-7 breast cancer cells (continued)

Gene symbol	Gene name	Location of latent 5'SS	Intron number	Position of latent 5'SS[a]	Number of stop codons[b]	Latent score[c]	Authentic score[c]	Log score ratio[d]	Up in 1a[e]	Up in 1b[e]	Up in 1c[e]
NOP58	NOP58 ribonucleoprotein homolog (yeast)	chr2:202849072	2	916	25	93.27	90.42	0.04		+	+
NOP58	NOP58 ribonucleoprotein homolog (yeast)	chr2:202866775	9	905	19	88.65	89.19	-0.01		+	+
NOP58	NOP58 ribonucleoprotein homolog (yeast)	chr2:202869443	10	639	12	90.43	92.07	-0.03		+	+
NPC1	Niemann-Pick disease, type C1	chr18:19388628	9	91	3	94.65	94.6	0		+	+
NPC1	Niemann-Pick disease, type C1	chr18:19385237	10	351	7	97.23	94.17	0.05		+	+
NPC1	Niemann-Pick disease, type C1	chr18:19366561	24	755	12	89.9	92.83	-0.05		+	+
NPRL3	nitrogen permease regulator-like 3 (S. cerevisiae)	chr16:77819	11	877	12	92.23	94.41	-0.03		+	+
NPTX2	neuronal pentraxin II	chr7:98087803	2	697	13	98	91.02	0.11		+	+
NR1D1	nuclear receptor subfamily 1, group D, member 1	chr17:35503408	7	282	4	90.14	95.28	-0.08		+	+
NR1D2	nuclear receptor subfamily 1, group D, member 2	chr3:23976416	4	107	2	88.77	88.24	0.01		+	+
NUP133	nucleoporin 133kDa	chr1:227702322	4	803	25	94.43	95.28	-0.01		+	+
NUP153	nucleoporin 153kDa	chr6:17755474	13	542	8	90.81	82.89	0.13		+	+
NUP88	nucleoporin 88kDa	chr17:5262749	1	648	11	90.17	90.06	0		+	+
NXF1	nuclear RNA export factor 1	chr11:62324933	9	208	3	89.46	87.09	0.04		+	+
OIT3	oncoprotein induced transcript 3	chr10:74337218	4	737	9	89.06	95.23	-0.1		+	+
OSGEP	O-sialoglycoprotein endopeptidase	chr14:19986866	5	96	2	90.99	91.79	-0.01		+	+
OTOP3	otopetrin 3	chr17:70443627	1	31	1	97.7	81.17	0.27		+	+
PAH	phenylalanine hydroxylase	chr12:101758182	12	125	1	89.25	87.51	0.03		+	+
PAK2	p21 protein (Cdc42/Rac)-activated kinase 2	chr3:198024820	10	702	14	88.78	100	-0.17		+	+
PAPD7	PAP associated domain containing 7	chr5:6793643	4	679	15	90.42	93.73	-0.05		+	+
PDHX	pyruvate dehydrogenase complex, component X	chr11:34895835	1	898	8	91.4	92.71	-0.02		+	+
PEX14	peroxisomal biogenesis factor 14	chr1:10610337	8	331	2	89.06	83.85	0.09		+	+
PI4K2B	phosphatidylinositol 4-kinase type 2 beta	chr4:24875446	7	878	18	91.43	90.68	0.01		+	+
PIP4K2A	phosphatidylinositol-5-phosphate 4-kinase, type II, alpha	chr10:22870081	8	657	9	90.96	91.78	-0.01		+	+
PLOD1	procollagen-lysine 1, 2-oxoglutarate 5-dioxygenase 1	chr1:11940438	7	781	6	92.64	92.37	0		+	+
PLOD1	procollagen-lysine 1, 2-oxoglutarate 5-dioxygenase 1	chr1:11948064	13	636	3	90.94	89.1	0.03		+	+
PLOD3	procollagen-lysine, 2-oxoglutarate 5-dioxygenase 3	chr7:100638537	16	316	1	91.49	96.05	-0.07		+	+
PLP2	proteolipid protein 2 (colonic epithelium-enriched)	chrX:48915501	1	115	1	94.41	82.55	0.19		+	+
PMM1	phosphomannomutase 1	chr22:40310077	4	153	2	92.64	90.92	0.03		+	+
PNPLA3	patatin-like phospholipase domain containing 3	chr22:42672759	8	752	11	90.12	92.54	-0.04		+	+
POLD3	polymerase (DNA-directed), delta 3, accessory subunit	chr11:73981870	1	460	6	93.12	81.18	0.2		+	+

Supplementary Table 3. List of latent 5'SSs activated in MCF-7 breast cancer cells (continued)

Gene symbol	Gene name	Location of latent 5'SS	Intron number	Position of latent 5'SS[a]	Number of stop codons[b]	Latent score[c]	Authentic score[c]	Log score ratio[d]	Up in 1a[e]	Up in 1b[e]	Up in 1c[e]
PRRG4	proline rich Gla (G-carboxyglutamic acid) 4 (transmembrane)	chr11:32819152	5	792	20	88.65	94.17	-0.09		+	+
PSMD3	proteasome (prosome, macropain) 26S subunit, non-ATPase, 3	chr17:35405484	9	179	2	90.68	91.5	-0.01		+	+
PSMD5	proteasome (prosome, macropain) 26S subunit, non-ATPase, 5	chr9:122634611	2	799	19	89.46	91.3	-0.03		+	+
PSME4	proteasome (prosome, macropain) activator subunit 4	chr2:53988123	24	837	22	88.9	87.16	0.03		+	+
PSTK	phosphoseryl-tRNA kinase	chr10:124736796	5	329	8	97.1	90.17	0.11		+	+
PTPN4	protein tyrosine phosphatase, non-receptor type 4 (megakaryocyte)	chr2:120351922	5	335	6	89.49	93.27	-0.06		+	+
PTPRM	protein tyrosine phosphatase, receptor type, M	chr18:7558666	1	778	4	91.64	97.7	-0.09		+	+
PTPRM	protein tyrosine phosphatase, receptor type, M	chr18:8079062	11	214	7	92.04	92.3	0		+	+
PWP2	PWP2 periodic tryptophan protein homolog (yeast)	chr21:44360712	7	484	4	89.71	88.19	0.02		+	+
PYGB	phosphorylase, glycogen; brain	chr20:25177494	1	438	4	90.41	96.22	-0.09		+	+
PYGB	phosphorylase, glycogen; brain	chr20:25206331	7	370	1	88.65	88.18	0.01		+	+
RBM45	RNA binding motif protein 45	chr2:178696715	6	92	4	96.36	87.54	0.14		+	+
REEP5	receptor accessory protein 5	chr5:112250116	4	494	9	94.07	96.1	-0.03		+	+
RIOK3	RIO kinase 3 (yeast)	chr18:19297794	2	754	16	91.41	81.55	0.16		+	+
RNASEK	ribonuclease, RNase K	chr17:6857247	1	444	3	91.37	88.27	0.05		+	+
RNF125	ring finger protein 125	chr18:27900176	5	207	6	89.93	87.08	0.05		+	+
RPA1	replication protein A1, 70kDa	chr17:1742818	15	835	10	91.09	87.96	0.05		+	+
RPL35	ribosomal protein L35	chr9:126663712	1	297	2	89.9	90.53	-0.01		+	+
SCNN1B	sodium channel, nonvoltage-gated 1, beta	chr16:23272568	3	673	16	88.9	88.22	0.01		+	+
SCNN1B	sodium channel, nonvoltage-gated 1, beta	chr16:23295497	8	821	5	90.39	90.41	0		+	+
SDHB	succinate dehydrogenase complex, subunit B, iron sulfur (Ip)	chr1:17226674	5	156	1	94.89	78.53	0.27		+	+
SEC24A	SEC24 family, member A (S. cerevisiae)	chr5:134031499	3	915	19	89.01	98.87	-0.15		+	+
SEMA3C	sema domain, immunoglobulin domain (Ig), short basic domain, secreted, (semaphorin) 3C	chr7:80224737	15	845	19	92.26	91.43	0.01		+	+
SESTD1	SEC14 and spectrin domains 1	chr2:179685173	17	522	8	94.07	83.66	0.17		+	+
SH2D3A	SH2 domain containing 3A	chr19:6709903	4	701	7	94.63	91.36	0.05		+	+
SH3PXD2A	SH3 and PX domains 2A	chr10:105376415	9	420	3	91.43	96.22	-0.07		+	+
SIK1	salt-inducible kinase 1	chr21:43669579	3	135	3	91.41	98.23	-0.1		+	+
SKAP2	src kinase associated phosphoprotein 2	chr7:26869700	1	806	9	91.02	90.77	0		+	+
SKAP2	src kinase associated phosphoprotein 2	chr7:26732626	7	399	14	88.64	74.68	0.25		+	+
SKAP2	src kinase associated phosphoprotein 2	chr7:26695785	10	643	13	88.59	90.06	-0.02		+	+

Supplementary Table 3. List of latent 5'SSs activated in MCF-7 breast cancer cells (continued)

Gene symbol	Gene name	Location of latent 5'SS	Intron number	Position of latent 5'SS[a]	Number of stop codons[b]	Latent score[c]	Authentic score[c]	Log score ratio[d]	Up in 1a[e]	Up in 1b[e]	Up in 1c[e]
SLC25A20	solute carrier family 25 (carnitine/acylcarnitine translocase), member 20	chr3:48870756	8	187	2	95.18	92.69	0.04		+	+
SLC25A23	solute carrier family 25 (mitochondrial carrier; phosphate carrier), member 23	chr19:6394200	9	961	18	90.01	96.22	-0.1		+	+
SLC2A1	solute carrier family 2 (facilitated glucose transporter), member 1	chr1:43168484	4	399	2	88.86	97.7	-0.14		+	+
SLC30A4	solute carrier family 30 (zinc transporter), member 4	chr15:43565918	7	182	3	94.63	82.58	0.2		+	+
SLC35A5	solute carrier family 35, member A5	chr3:113765259	2	190	4	89.55	98.87	-0.14		+	+
SLC38A3	solute carrier family 38, member 3	chr3:50230921	12	116	1	96.95	79.88	0.28		+	+
SLC6A18	solute carrier family 6, member 18	chr5:1296374	8	397	1	91.43	85.23	0.1		+	+
SLC7A1	solute carrier family 7 (cationic amino acid transporter, y+ system), member 1	chr13:29007088	3	867	9	90.24	86.62	0.06		+	+
SMARCC1	SWI/SNF related, matrix associated, actin dependent regulator of chromatin, subfamily c, member 1	chr3:47744832	6	686	10	92.23	94.17	-0.03		+	+
SMCHD1	structural maintenance of chromosomes flexible hinge domain containing 1	chr18:2751273	9	537	10	89.93	82.12	0.13		+	+
SMCHD1	structural maintenance of chromosomes flexible hinge domain containing 1	chr18:2768953	18	717	15	88.92	91.89	-0.05		+	+
SNX9	sorting nexin 9	chr6:158282599	17	606	4	98.23	93.73	0.07		+	+
SPTBN5	spectrin, beta, non-erythrocytic 5	chr15:39931597	62	74	1	90.94	82.08	0.15		+	+
SPTLC2	serine palmitoyltransferase, long chain base subunit 2	chr14:77097857	6	634	17	88.8	86.64	0.04		+	+
SRRM2	serine/arginine repetitive matrix 2	chr16:2751218	10	718	20	88.77	91.89	-0.05		+	+
SS18L1	synovial sarcoma translocation gene on chromosome 18-like 1	chr20:60167668	2	470	5	91.64	85.6	0.1		+	+
ST6GALNAC5	ST6 (alpha-N-acetyl-neuraminyl-2,3-beta-galactosyl-1,3)-N-acetylgalactosaminide alpha-2,6-sialyltransferase 5	chr1:77283601	3	716	12	93.83	83.91	0.16		+	+
STX18	syntaxin 18	chr4:4472892	10	599	8	96.56	90.8	0.09		+	+
TBC1D4	TBC1 domain family, member 4	chr13:74833767	2	395	3	90.7	84.38	0.1		+	+
TELO2	TEL2, telomere maintenance 2, homolog (S. cerevisiae)	chr16:1489629	6	295	6	92.96	91.96	0.02		+	+
TELO2	TEL2, telomere maintenance 2, homolog (S. cerevisiae)	chr16:1493271	15	268	4	97.36	89.38	0.12		+	+
TFB1M	transcription factor B1, mitochondrial	chr6:155647794	5	189	6	89.11	97.82	-0.13		+	+
THG1L	tRNA-histidine guanylyltransferase 1-like (S. cerevisiae)	chr5:157092198	1	982	16	90.36	88.17	0.04		+	+
TMEM104	transmembrane protein 104	chr17:70293771	3	435	4	90.69	94.18	-0.05		+	+
TMEM206	transmembrane protein 206	chr1:210624997	4	241	1	89.92	84.95	0.08		+	+

Supplementary Table 3. List of latent 5'SSs activated in MCF-7 breast cancer cells (continued)

Gene symbol	Gene name	Location of latent 5'SS	Intron number	Position of latent 5'SS[a]	Number of stop codons[b]	Latent score[c]	Authentic score[c]	Log score ratio[d]	Up in 1a[e]	Up in 1b[e]	Up in 1c[e]
TNKS	tankyrase, TRF1-interacting ankyrin-related ADP-ribose polymerase	chr8:9665575	26	394	9	90.42	94.07	-0.06		+	+
TONSL	tonsoku-like, DNA repair protein	chr8:145635935	11	276	3	89.94	90.92	-0.02		+	+
TOP2B	topoisomerase (DNA) II beta 180kDa	chr3:25621837	32	700	18	96.56	88.24	0.13		+	+
TPP2	tripeptidyl peptidase II	chr13:102097124	20	384	7	91.64	92.91	-0.02		+	+
TPR	translocated promoter region (to activated MET oncogene)	chr1:184559181	43	259	3	89.27	83.13	0.1		+	+
TRIP13	thyroid hormone receptor interactor 13	chr5:965710	10	600	9	92.69	85.23	0.12		+	+
TSPEAR	thrombospondin-type laminin G domain and EAR repeats	chr21:44752564	10	945	8	88.72	91.77	-0.05		+	+
TSR1	TSR1, 20S rRNA accumulation, homolog (S. cerevisiae)	chr17:2183999	5	516	11	91.96	95.05	-0.05		+	+
TTC37	tetratricopeptide repeat domain 37	chr5:94901872	8	277	6	88.67	75.03	0.24		+	+
TTC37	tetratricopeptide repeat domain 37	chr5:94881792	20	383	4	90.36	75.39	0.26		+	+
TTC37	tetratricopeptide repeat domain 37	chr5:94867470	30	923	21	90.42	91.87	-0.02		+	+
TTC38	tetratricopeptide repeat domain 38	chr22:45056709	7	431	7	93.2	90.17	0.05		+	+
UBL3	ubiquitin-like 3	chr13:29320890	1	758	18	92.86	94.17	-0.02		+	+
UBXN2A	UBX domain protein 2A	chr2:24054345	4	897	6	88.96	92.71	-0.06		+	+
UHRF1BP1	UHRF1 binding protein 1	chr6:34931661	9	204	4	89.86	88.58	0.02		+	+
URB2	URB2 ribosome biogenesis 2 homolog (S. cerevisiae)	chr1:227854317	8	626	13	92.23	90.69	0.02		+	+
WDR73	WD repeat domain 73	chr15:82996520	3	428	10	95.05	91.09	0.06		+	+
WRN	Werner syndrome, RecQ helicase-like	chr8:31110395	24	832	15	93.3	82.89	0.17		+	+
ZDHHC2	zinc finger, DHHC-type containing 2	chr8:17059293	1	561	6	94.65	94.73	0		+	+
ZNF236	zinc finger protein 236	chr18:72778483	26	197	5	88.64	86.8	0.03		+	+
ZNF354C	zinc finger protein 354C	chr5:178437646	4	874	19	88.65	95.6	-0.11		+	+

[a]Number of nt downstream from the authentic 5'SS.
[b]Number of in frame stop codons within the latent exon.
[c]See Methods.
[d]Latent score divided by authentic score.
[e]1a, BC1a; 1b, BC1b; 1c, BC1c.

Supplementary Table 4. List of latent 5'SSs activated in gliomas

Gene symbol	Gene name	Location of latent 5'SS	Intron number	Position of latent 5'SS[a]	Number of stop codons[b]	Latent score[c]	Authentic score[c]	Log score ratio[d]	Up in GBM	Up in ODII	Up in ODIII
AAK1	AP2 associated kinase 1	chr2:69611005	7	255	7	92.54	86.64	0.1	+	+	+
ADAM23	ADAM metallopeptidase domain 23	chr2:207144652	16	873	13	98.23	88.4	0.15	+	+	+
ADRBK1	adrenergic, beta, receptor kinase 1	chr11:66808853	18	434	1	92.64	96.22	-0.05	+	+	+
AGXT2L1	alanine-glyoxylate aminotransferase 2-like 1	chr4:109896717	4	305	8	91.55	88.65	0.05	+	+	+
AGXT2L1	alanine-glyoxylate aminotransferase 2-like 1	chr4:109891044	7	496	5	91.5	81.61	0.17	+	+	+
AK095053	AK095053	chr18:10737953	1	515	10	90.46	93.27	-0.04	+	+	+
AKAP6	A kinase (PRKA) anchor protein 6	chr14:32139456	6	994	15	89.19	92.54	-0.05	+	+	+
ALOXE3	arachidonate lipoxygenase 3	chr17:7960206	2	612	13	89.14	92.11	-0.05	+	+	+
ANO3	anoctamin 3	chr11:26510433	8	975	29	94.18	87.78	0.1	+	+	+
ANO3	anoctamin 3	chr11:26516125	10	542	7	96.1	89.65	0.1	+	+	+
AP3B2	adaptor-related protein complex 3, beta 2 subunit	chr15:81130854	8	409	3	93.51	86.92	0.11	+	+	+
APBA1	amyloid beta (A4) precursor protein-binding, family A, member 1	chr9:71279803	3	980	10	92.54	91.33	0.02	+	+	+
AQP2	aquaporin 2 (collecting duct)	chr12:48635179	3	420	9	94.63	81.42	0.22	+	+	+
ARFGEF2	ADP-ribosylation factor guanine nucleotide-exchange factor 2 (brefeldin A-inhibited)	chr20:47035201	15	418	4	89.63	79.75	0.17	+	+	+
ARHGEF12	Rho guanine nucleotide exchange factor (GEF) 12	chr11:119714022	1	829	19	92.86	85.63	0.12	+	+	+
ASPDH	aspartate dehydrogenase domain containing	chr19:55708842	1	704	8	90.47	88.24	0.04	+	+	+
ASPDH	aspartate dehydrogenase domain containing	chr19:55707731	2	264	2	93.12	93	0	+	+	+
ATG2B	ATG2 autophagy related 2 homolog B (S. cerevisiae)	chr14:95860851	15	887	24	89.49	88.14	0.02	+	+	+
ATP1A3	ATPase, Na+/K+ transporting, alpha 3 polypeptide	chr19:47165828	18	348	3	96.36	88.27	0.13	+	+	+
ATP5F1	ATP synthase, H+ transporting, mitochondrial Fo complex, subunit B1	chr1:111799488	3	988	21	89.77	96.83	-0.11	+	+	+
ATP6V1D	ATPase, H+ transporting, lysosomal 34kDa, V1 subunit D	chr14:66884925	4	581	11	89.93	90.46	-0.01	+	+	+
AVL9	AVL9 homolog (S. cerevisiase)	chr7:32561380	8	648	12	95.05	90.23	0.08	+	+	+
BMP2	bone morphogenetic protein 2	chr20:6699874	2	756	7	90.5	96.22	-0.09	+	+	+
BRAF	v-raf murine sarcoma viral oncogene homolog B1	chr7:140140050	8	526	15	91.53	89.38	0.03	+	+	+

Supplementary Table 4. List of latent 5'SSs activated in gliomas (continued)

Gene symbol	Gene name	Location of latent 5'SS	Intron number	Position of latent 5'SS[a]	Number of stop codons[b]	Latent score[c]	Authentic score[c]	Log score ratio[d]	Up in GBM	Up in ODII	Up in ODIII
CA11	carbonic anhydrase XI	chr19:53838946	3	549	8	88.65	97.1	-0.13	+	+	+
CALY	calcyon neuron-specific vesicular protein	chr10:134990844	3	554	3	94.43	84.55	0.16	+	+	+
CAMSAP1	calmodulin regulated spectrin-associated protein 1	chr9:137897440	3	682	8	90.49	89.64	0.01	+	+	+
CAP2	CAP, adenylate cyclase-associated protein, 2 (yeast)	chr6:17648192	8	525	4	89.46	95.23	-0.09	+	+	+
CCDC64	coiled-coil domain containing 64	chr12:118984572	3	560	5	88.77	94.65	-0.09	+	+	+
CDKL2	cyclin-dependent kinase-like 2 (CDC2-related kinase)	chr4:76740097	10	357	5	89.49	83.82	0.09	+	+	+
CHD1	chromodomain helicase DNA binding protein 1	chr5:98232619	29	738	16	90.63	89.01	0.03	+	+	+
CLMP	CXADR-like membrane protein	chr11:122450499	6	120	6	90.58	81.58	0.15	+	+	+
COX7A2L	cytochrome c oxidase subunit VIIa polypeptide 2 like	chr2:42433139	2	716	23	90.36	97.36	-0.11	+	+	+
CPNE9	copine family member IX	chr3:9734058	14	271	5	92.26	90.53	0.03	+	+	+
CRABP1	cellular retinoic acid binding protein 1	chr15:76420983	2	366	1	89.9	91.99	-0.03	+	+	+
CYB5R2	cytochrome b5 reductase 2	chr11:7643432	8	825	11	89.04	82.27	0.11	+	+	+
CYP7B1	cytochrome P450, family 7, subfamily B, polypeptide 1	chr8:65678855	5	937	19	90.54	97.82	-0.11	+	+	+
DCC	deleted in colorectal carcinoma	chr18:49197124	21	601	5	90.77	91.17	-0.01	+	+	+
DENND4B	DENN/MADD domain containing 4B	chr1:152171019	24	284	4	89.46	91.4	-0.03	+	+	+
DEPTOR	DEP domain containing MTOR-interacting protein	chr8:121083454	5	325	7	89.46	91.77	-0.04	+	+	+
DHCR24	24-dehydrocholesterol reductase	chr1:55103265	6	298	3	91.43	97.36	-0.09	+	+	+
DOCK3	dedicator of cytokinesis 3	chr3:51172511	10	737	13	88.61	79.79	0.15	+	+	+
DRP2	dystrophin related protein 2	chrX:100384837	9	211	3	89.93	81.43	0.14	+	+	+
EFHD2	EF-hand domain family, member D2	chr1:15627176	3	810	9	90.54	96.22	-0.09	+	+	+
EIF2B2	eukaryotic translation initiation factor 2B, subunit 2 beta, 39kDa	chr14:74541728	4	373	4	91.99	98.23	-0.09	+	+	+
EMID2	EMI domain containing 2	chr7:100970908	5	859	15	89.46	94.87	-0.08	+	+	+
EPB49	erythrocyte membrane protein band 4.9 (dematin)	chr8:21994199	12	284	3	91.81	42.29	1.12	+	+	+
EPHA4	EPH receptor A4	chr2:222144301	1	820	2	90.46	96.1	-0.09	+	+	+
EPHX4	epoxide hydrolase 4	chr1:92281997	3	873	22	91.46	90.88	0.01	+	+	+
ESYT3	extended synaptotagmin-like protein 3	chr3:139669279	11	139	1	88.72	82.73	0.1	+	+	+

Supplementary Table 4. List of latent 5'SSs activated in gliomas (continued)

Gene symbol	Gene name	Location of latent 5'SS	Intron number	Position of latent 5'SS[a]	Number of stop codons[b]	Latent score[c]	Authentic score[c]	Log score ratio[d]	Up in GBM	Up in ODII	Up in ODIII
ESYT3	extended synaptotagmin-like protein 3	chr3:139676731	20	848	16	90.14	88	0.03	+	+	+
EXOSC1	exosome component 1	chr10:99187077	6	369	7	91.5	77.12	0.25	+	+	+
FAAH	fatty acid amide hydrolase	chr1:46649775	11	663	9	89.94	89.57	0.01	+	+	+
FABP3	fatty acid binding protein 3, muscle and heart (mammary-derived growth inhibitor)	chr1:31611882	3	944	6	91.53	92.54	-0.02	+	+	+
FN3KRP	fructosamine 3 kinase related protein	chr17:78271095	2	874	3	90.97	85.81	0.08	+	+	+
FN3KRP	fructosamine 3 kinase related protein	chr17:78272127	3	600	8	90.49	83.66	0.11	+	+	+
FRS3	fibroblast growth factor receptor substrate 3	chr6:41847929	5	584	7	93.12	91.53	0.02	+	+	+
GABRD	gamma-aminobutyric acid (GABA) A receptor, delta	chr1:1950098	6	508	3	90.41	85.19	0.09	+	+	+
GHITM	growth hormone inducible transmembrane protein	chr10:85894322	4	481	4	88.65	82.92	0.1	+	+	+
GNA14	guanine nucleotide binding protein (G protein), alpha 14	chr9:79233223	5	419	6	91.67	89.59	0.03	+	+	+
GPR142	G protein-coupled receptor 142	chr17:69877365	2	50	1	98.23	89.06	0.14	+	+	+
GRIN2B	glutamate receptor, ionotropic, N-methyl D-aspartate 2B	chr12:13909903	2	95	2	88.79	88.64	0	+	+	+
HERC1	hect (homologous to the E6-AP (UBE3A) carboxyl terminus) domain and RCC1 (CHC1)-like domain (RLD) 1	chr15:61714460	65	752	12	91.43	89.63	0.03	+	+	+
HPRT1	hypoxanthine phosphoribosyltransferase 1	chrX:133448713	4	488	14	91.99	92.94	-0.01	+	+	+
HTT	huntingtin	chr4:3103627	17	328	9	93.76	89.37	0.07	+	+	+
HTT	huntingtin	chr4:3176014	43	342	8	91.78	83.31	0.14	+	+	+
IKZF5	IKAROS family zinc finger 5 (Pegasus)	chr10:124745034	4	465	2	88.63	90.06	-0.02	+	+	+
IQCK	IQ motif containing K	chr16:19653504	4	855	15	90.5	90.68	0	+	+	+
ISCU	iron-sulfur cluster scaffold homolog (E. coli)	chr12:107482749	3	453	8	93.3	92.3	0.02	+	+	+
ISL1	ISL LIM homeobox 1	chr5:50722082	4	560	1	89.9	85.29	0.08	+	+	+
KCNH3	potassium voltage-gated channel, subfamily H (eag-related), member 3	chr12:48219888	1	347	1	91.81	85.07	0.11	+	+	+
KCNH3	potassium voltage-gated channel, subfamily H (eag-related), member 3	chr12:48223889	5	322	6	88.61	84.17	0.07	+	+	+
KCNQ3	potassium voltage-gated channel, KQT-like subfamily, member 3	chr8:133266939	2	580	6	88.65	92.54	-0.06	+	+	+
KCNQ3	potassium voltage-gated channel, KQT-like subfamily, member 3	chr8:133215153	13	565	10	89.46	92.64	-0.05	+	+	+

Supplementary Table 4. List of latent 5'SSs activated in gliomas (continued)

Gene symbol	Gene name	Location of latent 5'SS	Intron number	Position of latent 5'SS[a]	Number of stop codons[b]	Latent score[c]	Authentic score[c]	Log score ratio[d]	Up in GBM	Up in ODII	Up in ODIII
KEL	Kell blood group, metallo-endopeptidase	chr7:142360541	9	475	2	91.43	80	0.19	+	+	+
KIAA0319	KIAA0319	chr6:24685416	9	900	13	88.72	86.8	0.03	+	+	+
KIAA0319	KIAA0319	chr6:24664624	18	189	2	89.46	92.26	-0.04	+	+	+
KIAA1499	KIAA1499	chr17:77143940	2	940	8	96.22	93.26	0.05	+	+	+
KIF5A	kinesin family member 5A	chr12:56261752	25	135	3	94.14	85.17	0.14	+	+	+
KLHL35	kelch-like 35 (Drosophila)	chr11:74811861	5	522	13	94.18	93.13	0.02	+	+	+
KNTC1	kinetochore associated 1	chr12:121642512	42	560	5	89.25	84.27	0.08	+	+	+
LANCL1	LanC lantibiotic synthetase component C-like 1 (bacterial)	chr2:211048873	2	411	11	92.4	93.76	-0.02	+	+	+
LCT	lactase	chr2:136297511	4	429	4	89.71	98	-0.13	+	+	+
LPCAT4	lysophosphatidylcholine acyltransferase 4	chr15:32444773	2	278	3	93.12	90.73	0.04	+	+	+
LPCAT4	lysophosphatidylcholine acyltransferase 4	chr15:32443994	3	506	5	90.39	94.18	-0.06	+	+	+
LPCAT4	lysophosphatidylcholine acyltransferase 4	chr15:32441511	10	177	4	88.9	91.36	-0.04	+	+	+
LPCAT4	lysophosphatidylcholine acyltransferase 4	chr15:32439904	11	988	20	89.06	90.77	-0.03	+	+	+
LPCAT4	lysophosphatidylcholine acyltransferase 4	chr15:32439293	12	310	2	91.99	87.88	0.07	+	+	+
LPIN2	lipin 2	chr18:2927325	7	364	5	88.65	88.39	0	+	+	+
LRP2	low density lipoprotein receptor-related protein 2	chr2:169884775	2	757	14	89.37	93.56	-0.07	+	+	+
LRP2	low density lipoprotein receptor-related protein 2	chr2:169805262	25	481	14	91.3	86.47	0.08	+	+	+
LRP2	low density lipoprotein receptor-related protein 2	chr2:169739177	55	771	12	90.63	86.33	0.07	+	+	+
LRP2	low density lipoprotein receptor-related protein 2	chr2:169736479	58	291	4	89.18	92.26	-0.05	+	+	+
LRRC40	leucine rich repeat containing 40	chr1:70418521	5	835	18	88.63	84.32	0.07	+	+	+
M6PR	mannose-6-phosphate receptor (cation dependent)	chr12:8985999	6	279	5	96.22	86.47	0.15	+	+	+
MAP7	microtubule-associated protein 7	chr6:136750271	5	952	31	91.36	90.24	0.02	+	+	+
MAP7	microtubule-associated protein 7	chr6:136739908	7	677	14	89.04	89.77	-0.01	+	+	+
MAP9	microtubule-associated protein 9	chr4:156508421	5	766	13	95.18	85.87	0.15	+	+	+
MET	met proto-oncogene (hepatocyte growth factor receptor)	chr7:116197942	12	862	15	90.68	89.46	0.02	+	+	+
MGC42105	serine/threonine-protein kinase NIM1	chr5:43282100	2	175	4	88.91	91.37	-0.04	+	+	+
MKI67IP	MKI67 (FHA domain) interacting nucleolar phosphoprotein	chr2:122210229	1	562	8	96.05	96.83	-0.01	+	+	+
MPO	myeloperoxidase	chr17:53709210	7	976	11	90.43	89.1	0.02	+	+	+

Supplementary Table 4. List of latent 5'SSs activated in gliomas (continued)

Gene symbol	Gene name	Location of latent 5'SS	Intron number	Position of latent 5'SS[a]	Number of stop codons[b]	Latent score[c]	Authentic score[c]	Log score ratio[d]	Up in GBM	Up in ODII	Up in ODIII
NCL	nucleolin	chr2:232030957	8	263	6	95.18	88.7	0.1	+	+	+
NDUFS1	NADH dehydrogenase (ubiquinone) Fe-S protein 1, 75kDa (NADH-coenzyme Q reductase)	chr2:206700244	16	521	8	90.43	81.66	0.15	+	+	+
NEFM	neurofilament, medium polypeptide	chr8:24828712	1	422	4	89.1	92.51	-0.05	+	+	+
NIPSNAP1	nipsnap homolog 1 (C. elegans)	chr22:28306511	1	462	7	92.64	89.92	0.04	+	+	+
NUDCD3	NudC domain containing 3	chr7:44495948	1	584	8	90.83	97.36	-0.1	+	+	+
OCA2	oculocutaneous albinism II	chr15:25872961	17	937	16	96.05	82.93	0.21	+	+	+
OGDHL	oxoglutarate dehydrogenase-like	chr10:50630384	4	305	3	91.66	88.59	0.05	+	+	+
OGDHL	oxoglutarate dehydrogenase-like	chr10:50621615	14	430	3	89.94	92.66	-0.04	+	+	+
OIT3	oncoprotein induced transcript 3	chr10:74337218	4	737	9	89.06	95.23	-0.1	+	+	+
ORC2	origin recognition complex, subunit 2	chr2:201503610	11	696	17	90.36	87.51	0.05	+	+	+
PAH	phenylalanine hydroxylase	chr12:101758182	12	125	1	89.25	87.51	0.03	+	+	+
PCNXL2	pecanex-like 2 (Drosophila)	chr1:231410574	7	312	6	90.14	94.73	-0.07	+	+	+
PEX14	peroxisomal biogenesis factor 14	chr1:10606514	6	750	14	92.64	90.77	0.03	+	+	+
PEX14	peroxisomal biogenesis factor 14	chr1:10610337	8	331	2	89.06	83.85	0.09	+	+	+
PIK3CB	phosphoinositide-3-kinase, catalytic, beta polypeptide	chr3:139885554	15	602	18	95.23	88.22	0.11	+	+	+
PIK3CB	phosphoinositide-3-kinase, catalytic, beta polypeptide	chr3:139858589	20	632	7	88.84	94.87	-0.09	+	+	+
PIP5K1C	phosphatidylinositol-4-phosphate 5-kinase, type I, gamma	chr19:3615528	3	291	1	92.66	75.58	0.29	+	+	+
PLK2	polo-like kinase 2	chr5:57790469	2	99	2	89.18	92.13	-0.05	+	+	+
PPFIA2	protein tyrosine phosphatase, receptor type, f polypeptide (PTPRF), interacting protein (liprin), alpha 2	chr12:80286014	13	620	17	92.37	88.64	0.06	+	+	+
PPL	periplakin	chr16:4878548	19	431	5	95.23	95.69	-0.01	+	+	+
PRDM4	PR domain containing 4	chr12:106673775	3	963	23	88.65	99.13	-0.16	+	+	+
PRKAA2	protein kinase, AMP-activated, alpha 2 catalytic subunit	chr1:56931599	4	837	29	90.46	85.41	0.08	+	+	+
PRKAR2B	protein kinase, cAMP-dependent, regulatory, type II, beta	chr7:106585814	10	810	16	91.83	89.11	0.04	+	+	+

Supplementary Table 4. List of latent 5'SSs activated in gliomas (continued)

Gene symbol	Gene name	Location of latent 5'SS	Intron number	Position of latent 5'SS[a]	Number of stop codons[b]	Latent score[c]	Authentic score[c]	Log score ratio[d]	Up in GBM	Up in ODII	Up in ODIII
PRPF4B	PRP4 pre-mRNA processing factor 4 homolog B (yeast)	chr6:4004799	14	753	14	92.37	94.63	-0.03	+	+	+
PTGDS	prostaglandin D2 synthase 21kDa (brain)	chr9:138994962	5	406	3	89.46	93.09	-0.06	+	+	+
PTPN4	protein tyrosine phosphatase, non-receptor type 4 (megakaryocyte)	chr2:120394927	12	641	15	92.54	95.3	-0.04	+	+	+
RAB26	RAB26, member RAS oncogene family	chr16:2142115	5	206	3	90.97	91.4	-0.01	+	+	+
RAB3A	RAB3A, member RAS oncogene family	chr19:18169848	4	686	13	90.96	90.04	0.01	+	+	+
RAB6B	RAB6B, member RAS oncogene family	chr3:135040318	5	721	15	90.01	93.1	-0.05	+	+	+
RAD21	RAD21 homolog (S. pombe)	chr8:117947543	2	462	13	89.45	92.94	-0.06	+	+	+
RDH12	retinol dehydrogenase 12 (all-trans/9-cis/11-cis)	chr14:67262995	4	371	6	90.8	91.36	-0.01	+	+	+
RELN	reelin	chr7:103038559	21	734	10	90.49	90.73	0	+	+	+
RELN	reelin	chr7:103001013	30	761	25	92.04	89.46	0.04	+	+	+
RELN	reelin	chr7:102946422	49	583	9	91.86	80.18	0.2	+	+	+
RNF125	ring finger protein 125	chr18:27900176	5	207	6	89.93	87.08	0.05	+	+	+
RYR3	ryanodine receptor 3	chr15:31811640	48	454	5	89.93	92.91	-0.05	+	+	+
SAP18	Sin3A-associated protein, 18kDa	chr13:20613484	2	351	8	92.4	88.27	0.07	+	+	+
SEC23A	Sec23 homolog A (S. cerevisiae)	chr14:38605603	10	524	12	88.65	96.1	-0.12	+	+	+
SEMA3A	sema domain, immunoglobulin domain (Ig), short basic domain, secreted, (semaphorin) 3A	chr7:83481258	7	202	7	90.05	96.22	-0.1	+	+	+
SGSM3	small G protein signaling modulator 3	chr22:39132907	10	296	2	89.71	84.33	0.09	+	+	+
SH3BGRL	SH3 domain binding glutamic acid-rich protein like	chrX:80344728	1	329	1	94.43	91.79	0.04	+	+	+
SHISA4	shisa homolog 4 (Xenopus laevis)	chr1:200126878	3	541	6	89.27	83.24	0.1	+	+	+
SIDT1	SID1 transmembrane family, member 1	chr3:114804879	12	193	2	88.61	82.93	0.1	+	+	+
SIDT1	SID1 transmembrane family, member 1	chr3:114810976	17	904	10	90.5	83.16	0.12	+	+	+
SLC22A9	solute carrier family 22 (organic anion transporter), member 9	chr11:62895044	1	539	10	90.06	95.05	-0.08	+	+	+
SLC25A12	solute carrier family 25 (mitochondrial carrier, Aralar), member 12	chr2:172408221	5	903	14	90.46	96.83	-0.1	+	+	+
SLC25A12	solute carrier family 25 (mitochondrial carrier, Aralar), member 12	chr2:172378981	10	895	10	90.43	96.95	-0.1	+	+	+

Supplementary Table 4. List of latent 5'SSs activated in gliomas (continued)

Gene symbol	Gene name	Location of latent 5'SS	Intron number	Position of latent 5'SS[a]	Number of stop codons[b]	Latent score[c]	Authentic score[c]	Log score ratio[d]	Up in GBM	Up in ODII	Up in ODIII
SLC25A23	solute carrier family 25 (mitochondrial carrier; phosphate carrier), member 23	chr19:6406574	4	856	7	91.66	91.96	0	+	+	+
SLC25A23	solute carrier family 25 (mitochondrial carrier; phosphate carrier), member 23	chr19:6394200	9	961	18	90.01	96.22	-0.1	+	+	+
SLC25A4	solute carrier family 25 (mitochondrial carrier; adenine nucleotide translocator), member 4	chr4:186304647	3	601	9	88.79	91.28	-0.04	+	+	+
SLC30A3	solute carrier family 30 (zinc transporter), member 3	chr2:27332014	3	743	18	92.71	90.47	0.04	+	+	+
SLC30A3	solute carrier family 30 (zinc transporter), member 3	chr2:27333799	4	477	1	94.18	78.82	0.26	+	+	+
SLC9A5	solute carrier family 9 (sodium/hydrogen exchanger), member 5	chr16:65847106	4	232	1	89.27	82.74	0.11	+	+	+
SORL1	sortilin-related receptor, L(DLR class) A repeats containing	chr11:120873666	6	699	16	92.64	93.13	-0.01	+	+	+
SORL1	sortilin-related receptor, L(DLR class) A repeats containing	chr11:120965706	29	398	11	90.96	90.79	0	+	+	+
SPTBN2	spectrin, beta, non-erythrocytic 2	chr11:66236870	7	807	12	88.63	91.5	-0.05	+	+	+
SRRM4	serine/arginine repetitive matrix 4	chr12:118039867	4	672	8	90.97	84.13	0.11	+	+	+
ST6GALNAC5	ST6 (alpha-N-acetyl-neuraminyl-2,3-beta-galactosyl-1,3)-N-acetylgalactosaminide alpha-2,6-sialyltransferase 5	chr1:77107220	2	206	3	90.94	87.45	0.06	+	+	+
ST6GALNAC5	ST6 (alpha-N-acetyl-neuraminyl-2,3-beta-galactosyl-1,3)-N-acetylgalactosaminide alpha-2,6-sialyltransferase 5	chr1:77283601	3	716	12	93.83	83.91	0.16	+	+	+
STS	steroid sulfatase (microsomal), isozyme S	chrX:7186035	4	407	12	95.23	87.9	0.12	+	+	+
SV2B	synaptic vesicle glycoprotein 2B	chr15:89596515	2	283	8	91.5	89.83	0.03	+	+	+
TBR1	T-box, brain, 1	chr2:161982841	2	255	6	92.07	85.67	0.1	+	+	+
TIMP3	TIMP metallopeptidase inhibitor 3	chr22:31528762	1	655	14	89.36	89.04	0.01	+	+	+
TMEM151B	transmembrane protein 151B	chr6:44349830	2	610	10	89.46	97.36	-0.12	+	+	+
TMEM63A	transmembrane protein 63A	chr1:224110732	17	243	4	88.65	86.61	0.03	+	+	+
TMOD2	tropomodulin 2 (neuronal)	chr15:49846441	2	386	11	89.65	88.17	0.02	+	+	+

Supplementary Table 4. List of latent 5'SSs activated in gliomas (continued)

Gene symbol	Gene name	Location of latent 5'SS	Intron number	Position of latent 5'SS[a]	Number of stop codons[b]	Latent score[c]	Authentic score[c]	Log score ratio[d]	Up in GBM	Up in ODII	Up in ODIII
TOX	thymocyte selection-associated high mobility group box	chr8:60014234	3	180	6	88.64	90.36	-0.03	+	+	+
TTLL7	tubulin tyrosine ligase-like family, member 7	chr1:84171328	9	550	9	88.81	96.05	-0.11	+	+	+
TTLL7	tubulin tyrosine ligase-like family, member 7	chr1:84158795	12	562	9	91.4	87.83	0.06	+	+	+
VDAC3	voltage-dependent anion channel 3	chr8:42381065	8	930	16	88.76	80.12	0.15	+	+	+
VPS18	vacuolar protein sorting 18 homolog (S. cerevisiae)	chr15:38974696	1	348	5	89.46	93.99	-0.07	+	+	+
WDR41	WD repeat domain 41	chr5:76822775	1	975	11	88.92	88.06	0.01	+	+	+
WDR66	WD repeat domain 66	chr12:120884919	16	469	4	89.19	97.7	-0.13	+	+	+
YIPF6	Yip1 domain family, member 6	chrX:67636344	1	655	12	93.26	92.13	0.02	+	+	+
ZBTB41	zinc finger and BTB domain containing 41	chr1:195411685	7	631	22	90.96	84.31	0.11	+	+	+
ZUFSP	zinc finger with UFM1-specific peptidase domain	chr6:117088090	3	501	9	93	92.94	0	+	+	+
AHSA1	AHA1, activator of heat shock 90kDa protein ATPase homolog 1 (yeast)	chr14:76999276	4	422	9	89.46	84	0.09	+	+	
APOA2	apolipoprotein A-II	chr1:159459557	2	206	2	90.12	93.27	-0.05	+	+	
ARL6IP1	ADP-ribosylation factor-like 6 interacting protein 1	chr16:18712653	5	768	15	91.99	95.6	-0.06	+	+	
ASPHD1	aspartate beta-hydroxylase domain containing 1	chr16:29824540	2	754	19	90.04	84.63	0.09	+	+	
ATCAY	ataxia, cerebellar, Cayman type	chr19:3854436	3	894	13	90.46	87.29	0.05	+	+	
BAIAP2L2	BAI1-associated protein 2-like 2	chr22:36812518	11	558	7	91.49	86.36	0.08	+	+	
CACNA1S	calcium channel, voltage-dependent, L type, alpha 1S subunit	chr1:199311904	12	766	8	91	92.98	-0.03	+	+	
CAMSAP1	calmodulin regulated spectrin-associated protein 1	chr9:137848742	14	901	12	91.4	91.79	-0.01	+	+	
CCDC120	coiled-coil domain containing 120	chrX:48807435	4	360	6	89.93	90.92	-0.02	+	+	
CHGB	chromogranin B (secretogranin 1)	chr20:5846060	3	496	9	90.22	88.67	0.02	+	+	
CLYBL	citrate lyase beta like	chr13:99315603	5	438	5	88.84	78.26	0.18	+	+	
DAGLA	diacylglycerol lipase, alpha	chr11:61264101	17	393	2	91.5	90.58	0.01	+	+	
DOCK1	dedicator of cytokinesis 1	chr10:129128328	49	850	11	92.66	84.53	0.13	+	+	
EIF4G2	eukaryotic translation initiation factor 4 gamma, 2	chr11:10776318	21	796	17	90.96	100.00	-0.14	+	+	
FAF1	Fas (TNFRSF6) associated factor 1	chr1:51197486	1	540	8	91.86	89.90	0.03	+	+	

Supplementary Table 4. List of latent 5'SSs activated in gliomas (continued)

Gene symbol	Gene name	Location of latent 5'SS	Intron number	Position of latent 5'SS[a]	Number of stop codons[b]	Latent score[c]	Authentic score[c]	Log score ratio[d]	Up in GBM	Up in ODII	Up in ODIII
FAM3D	family with sequence similarity 3, member D	chr3:58614132	3	308	2	89.46	90.49	-0.02	+	+	
GNA14	guanine nucleotide binding protein (G protein), alpha 14	chr9:79238468	3	635	11	88.64	86.21	0.04	+	+	
HDAC4	histone deacetylase 4	chr2:239676453	18	188	3	88.7	90.27	-0.03	+	+	
HERC1	hect (homologous to the E6-AP (UBE3A) carboxyl terminus) domain and RCC1 (CHC1)-like domain (RLD) 1	chr15:61753227	38	366	3	89.93	92.94	-0.05	+	+	
HERC1	hect (homologous to the E6-AP (UBE3A) carboxyl terminus) domain and RCC1 (CHC1)-like domain (RLD) 1	chr15:61712181	67	918	18	100	91.4	0.13	+	+	
KHDRBS2	KH domain containing, RNA binding, signal transduction associated 2	chr6:63053198	1	523	10	93.13	91.37	0.03	+	+	
LPIN2	lipin 2	chr18:2940183	4	869	13	89.37	87.11	0.04	+	+	
MRPS31	mitochondrial ribosomal protein S31	chr13:40242751	1	369	9	89.27	91.01	-0.03	+	+	
MYSM1	Myb-like, SWIRM and MPN domains 1	chr1:58906691	10	200	1	89.55	89.14	0.01	+	+	
NLGN2	neuroligin 2	chr17:7260455	6	306	3	93.20	88.72	0.07	+	+	
PIK3R2	phosphoinositide-3-kinase, regulatory subunit 2 (beta)	chr19:18134730	9	415	7	91.49	92.64	-0.02	+	+	
PLXNC1	plexin C1	chr12:93099769	3	283	6	91.09	93.10	-0.03	+	+	
PNPLA3	patatin-like phospholipase domain containing 3	chr22:42672759	8	752	11	90.12	92.54	-0.04	+	+	
PRPF19	PRP19/PSO4 pre-mRNA processing factor 19 homolog (S. cerevisiae)	chr11:60421288	15	605	17	94.17	86.05	0.13	+	+	
RAB14	RAB14, member RAS oncogene family	chr9:122995260	2	199	6	89.71	90.91	-0.02	+	+	
RAB40B	RAB40B, member RAS oncogene family	chr17:78249036	1	583	1	91.36	92.59	-0.02	+	+	
SNTG1	syntrophin, gamma 1	chr8:51827960	18	737	14	90.22	89.11	0.02	+	+	
TLE2	transducin-like enhancer of split 2 (E(sp1) homolog, Drosophila)	chr19:2950814	19	830	10	88.65	83.66	0.08	+	+	
TLL1	tolloid-like 1	chr4:167240335	20	207	4	88.9	86.13	0.05	+	+	
TNFRSF21	tumor necrosis factor receptor superfamily, member 21	chr6:47361158	2	480	7	92.64	91.09	0.02	+	+	
TP53TG5	TP53 target 5	chr20:43437064	4	28	1	91.96	93.27	-0.02	+	+	
TSPEAR	thrombospondin-type laminin G domain and EAR repeats	chr21:44752564	10	945	8	88.72	91.77	-0.05	+	+	
USP9Y	ubiquitin specific peptidase 9, Y-linked	chrY:13438371	32	699	7	90.46	90.36	0	+	+	
ZFYVE20	zinc finger, FYVE domain containing 20	chr3:15111641	4	842	18	90.33	89.01	0.02	+	+	

Supplementary Table 4. List of latent 5'SSs activated in gliomas (continued)

Gene symbol	Gene name	Location of latent 5'SS	Intron number	Position of latent 5'SS[a]	Number of stop codons[b]	Latent score[c]	Authentic score[c]	Log score ratio[d]	Up in GBM	Up in ODII	Up in ODIII
AGPAT5	1-acylglycerol-3-phosphate O-acyltransferase 5 (lysophosphatidic acid acyltransferase, epsilon)	chr8:6554503	1	688	8	90.54	96.83	-0.10	+		+
AGXT2L1	alanine-glyoxylate aminotransferase 2-like 1	chr4:109888535	9	74	1	90.53	89.67	0.01	+		+
ARFGEF2	ADP-ribosylation factor guanine nucleotide-exchange factor 2 (brefeldin A-inhibited)	chr20:47068412	33	919	21	88.65	83.42	0.09	+		+
ARL2	ADP-ribosylation factor-like 2	chr11:64543311	3	531	7	91.49	89.93	0.02	+		+
ATP1A3	ATPase, Na+/K+ transporting, alpha 3 polypeptide	chr19:47177241	11	252	3	90.46	83.66	0.11	+		+
ATP4B	ATPase, H+/K+ exchanging, beta polypeptide	chr13:113354885	4	303	4	90.77	93.76	-0.05	+		+
ATP6V0D1	ATPase, H+ transporting, lysosomal 38kDa, V0 subunit d1	chr16:66035478	3	453	2	88.63	87.73	0.01	+		+
ATP6V1D	ATPase, H+ transporting, lysosomal 34kDa, V1 subunit D	chr14:66879300	7	541	13	92.83	93.3	-0.01	+		+
AVL9	AVL9 homolog (S. cerevisiase)	chr7:32582686	13	478	12	89.93	89.48	0.01	+		+
BHLHE40	basic helix-loop-helix family, member e40	chr3:4998025	3	933	7	90.5	87.51	0.05	+		+
BHMT	betaine--homocysteine S-methyltransferase	chr5:78453794	5	851	12	90.43	89.19	0.02	+		+
BHMT2	betaine--homocysteine S-methyltransferase 2	chr5:78414744	5	210	6	90.42	91.83	-0.02	+		+
BOD1L	biorientation of chromosomes in cell division 1-like	chr4:13237698	1	368	4	91.79	87.44	0.07	+		+
BTBD8	BTB (POZ) domain containing 8	chr1:92377751	6	177	3	90.22	85.43	0.08	+		+
C5orf4	chromosome 5 open reading frame 4	chr5:154183017	6	122	4	89.93	92.56	-0.04	+		+
CASQ1	calsequestrin 1 (fast-twitch, skeletal muscle)	chr1:158434773	7	746	10	91.43	90.49	0.01	+		+
CASQ1	calsequestrin 1 (fast-twitch, skeletal muscle)	chr1:158437188	10	850	25	90.5	88.51	0.03	+		+
CDC42BPB	CDC42 binding protein kinase beta (DMPK-like)	chr14:102522177	6	399	6	92.23	81.43	0.18	+		+
CDCP2	CUB domain containing protein 2	chr1:54382562	2	164	1	96.36	80.51	0.26	+		+
CDKN1B	cyclin-dependent kinase inhibitor 1B (p27, Kip1)	chr12:12762704	1	190	5	91.4	84.66	0.11	+		+
CENPV	centromere protein V	chr17:16193843	2	126	4	89.19	92.04	-0.05	+		+
CSRP1	cysteine and glycine-rich protein 1	chr1:199731265	2	677	6	89.19	85.60	0.06	+		+

Supplementary Table 4. List of latent 5'SSs activated in gliomas (continued)

Gene symbol	Gene name	Location of latent 5'SS	Intron number	Position of latent 5'SS[a]	Number of stop codons[b]	Latent score[c]	Authentic score[c]	Log score ratio[d]	Up in GBM	Up in ODII	Up in ODIII
CTC1	CTS telomere maintenance complex component 1	chr17:8081927	4	146	4	89.64	91.83	-0.03	+		+
CTR9	Ctr9, Paf1/RNA polymerase II complex component, homolog (S. cerevisiae)	chr11:10729920	1	341	7	89.93	97.82	-0.12	+		+
DAGLA	diacylglycerol lipase, alpha	chr11:61252621	7	287	2	89.96	94.65	-0.07	+		+
DEPTOR	DEP domain containing MTOR-interacting protein	chr8:121011890	3	532	2	88.65	85.85	0.05	+		+
DHCR24	24-dehydrocholesterol reductase	chr1:55124880	1	269	1	92.23	96.83	-0.07	+		+
DOCK3	dedicator of cytokinesis 3	chr3:51273218	22	422	5	90.97	82.18	0.15	+		+
DOCK3	dedicator of cytokinesis 3	chr3:51290996	25	772	16	94.07	76.5	0.3	+		+
DOCK3	dedicator of cytokinesis 3	chr3:51387358	49	301	1	90.69	89.06	0.03	+		+
DYNC1LI1	dynein, cytoplasmic 1, light intermediate chain 1	chr3:32550177	7	829	17	89.46	87.16	0.04	+		+
DYNC1LI2	dynein, cytoplasmic 1, light intermediate chain 2	chr16:65342352	2	324	3	90.50	88.17	0.04	+		+
EFNA5	ephrin-A5	chr5:107033337	1	751	6	89.46	97.70	-0.13	+		+
EXTL1	exostoses (multiple)-like 1	chr1:26233832	9	899	20	95.18	80.51	0.24	+		+
FAM151B	family with sequence similarity 151, member B	chr5:79851985	4	501	9	90.97	87.92	0.05	+		+
FGD1	FYVE, RhoGEF and PH domain containing 1	chrX:54537523	1	760	8	93.20	94.43	-0.02	+		+
FIG4	FIG4 homolog, SAC1 lipid phosphatase domain containing (S. cerevisiae)	chr6:110172144	10	477	10	92.11	90.17	0.03	+		+
FOXO4	forkhead box O4	chrX:70238530	2	216	2	92.94	84.91	0.13	+		+
GHITM	growth hormone inducible transmembrane protein	chr10:85892093	2	729	20	89.67	85.4	0.07	+		+
HERC1	hect (homologous to the E6-AP (UBE3A) carboxyl terminus) domain and RCC1 (CHC1)-like domain (RLD) 1	chr15:61807512	16	975	18	97.7	96.95	0.01	+		+
HINT3	histidine triad nucleotide binding protein 3	chr6:126330403	2	561	14	88.65	88.59	0	+		+
HIVEP2	human immunodeficiency virus type I enhancer binding protein 2	chr6:143132276	5	105	2	90.27	89.46	0.01	+		+
HIVEP2	human immunodeficiency virus type I enhancer binding protein 2	chr6:143127485	7	143	3	92.91	82.77	0.17	+		+
HSPA4L	heat shock 70kDa protein 4-like	chr4:128961746	14	577	15	90.27	91.89	-0.03	+		+
JARID2	jumonji, AT rich interactive domain 2	chr6:15355074	1	281	10	90.46	85.78	0.08	+		+
KCNH3	potassium voltage-gated channel, subfamily H (eag-related), member 3	chr12:48221649	2	468	3	89.93	90.92	-0.02	+		+
LMTK2	lemur tyrosine kinase 2	chr7:97609630	3	842	27	89.01	92.83	-0.06	+		+

Supplementary Table 4. List of latent 5'SSs activated in gliomas (continued)

Gene symbol	Gene name	Location of latent 5'SS	Intron number	Position of latent 5'SS[a]	Number of stop codons[b]	Latent score[c]	Authentic score[c]	Log score ratio[d]	Up in GBM	Up in ODII	Up in ODIII
LPIN2	lipin 2	chr18:2914799	14	422	6	91.43	83.48	0.13	+		+
LRP1B	low density lipoprotein receptor-related protein 1B	chr2:141488737	13	997	24	95.23	88.64	0.10	+		+
MOCS2	molybdenum cofactor synthesis 2	chr5:52433424	4	259	1	88.64	94.73	-0.1	+		+
NDUFA4	NADH dehydrogenase (ubiquinone) 1 alpha subcomplex, 4, 9kDa	chr7:10945287	1	880	17	89.01	84.81	0.07	+		+
NEURL	neuralized homolog (Drosophila)	chr10:105335100	4	129	1	98.00	90.47	0.12	+		+
NOS1	nitric oxide synthase 1 (neuronal)	chr12:116209594	5	667	12	91.36	85.67	0.09	+		+
NPAS2	neuronal PAS domain protein 2	chr2:100947699	8	630	14	91.46	85.67	0.09	+		+
NR1D2	nuclear receptor subfamily 1, group D, member 2	chr3:23976416	4	107	2	88.77	88.24	0.01	+		+
NSUN3	NOP2/Sun domain family, member 3	chr3:95296056	4	229	7	92.23	84.83	0.12	+		+
PIP5K1B	phosphatidylinositol-4-phosphate 5-kinase, type I, beta	chr9:70694525	7	657	13	90.01	92.4	-0.04	+		+
PLCG2	phospholipase C, gamma 2 (phosphatidylinositol-specific)	chr16:80472990	8	932	16	88.65	90.94	-0.04	+		+
PLEKHM2	pleckstrin homology domain containing, family M (with RUN domain) member 2	chr1:15927969	12	155	1	90.81	90.47	0.01	+		+
PPFIA2	protein tyrosine phosphatase, receptor type, f polypeptide (PTPRF), interacting protein (liprin), alpha 2	chr12:80292897	10	808	9	90.36	75.25	0.26	+		+
PPP1R16B	protein phosphatase 1, regulatory (inhibitor) subunit 16B	chr20:36970877	10	628	14	89.46	86.42	0.05	+		+
RAB6B	RAB6B, member RAS oncogene family	chr3:135041993	4	825	7	91.64	92.04	-0.01	+		+
RAI1	retinoic acid induced 1	chr17:17653820	5	343	1	88.77	92.37	-0.06	+		+
RANBP3L	RAN binding protein 3-like	chr5:36300157	6	660	11	90.88	95.23	-0.07	+		+
RANBP3L	RAN binding protein 3-like	chr5:36292081	10	718	14	90.94	84.34	0.11	+		+
RANBP3L	RAN binding protein 3-like	chr5:36286656	13	515	12	90.01	92.54	-0.04	+		+
REEP5	receptor accessory protein 5	chr5:112285077	1	591	1	90.33	85.31	0.08	+		+
RHO	rhodopsin	chr3:130734554	4	250	2	91.64	85.49	0.1	+		+
RND1	Rho family GTPase 1	chr12:47545414	1	283	1	97.36	94.65	0.04	+		+
SCGN	secretagogin, EF-hand calcium binding protein	chr6:25791030	7	818	19	90.05	98.00	-0.12	+		+
SGSM3	small G protein signaling modulator 3	chr22:39135119	19	152	1	88.63	92.04	-0.05	+		+

Supplementary Table 4. List of latent 5'SSs activated in gliomas (continued)

Gene symbol	Gene name	Location of latent 5'SS	Intron number	Position of latent 5'SS[a]	Number of stop codons[b]	Latent score[c]	Authentic score[c]	Log score ratio[d]	Up in GBM	Up in ODII	Up in ODIII
SLC30A4	solute carrier family 30 (zinc transporter), member 4	chr15:43565918	7	182	3	94.63	82.58	0.2	+		+
SLC30A9	solute carrier family 30 (zinc transporter), member 9	chr4:41688413	1	880	8	91.43	93.76	-0.04	+		+
SORCS2	sortilin-related VPS10 domain containing receptor 2	chr4:7766107	15	628	6	89.01	92.11	-0.05	+		+
SORCS2	sortilin-related VPS10 domain containing receptor 2	chr4:7786668	24	621	1	92.64	96.83	-0.06	+		+
SORL1	sortilin-related receptor, L(DLR class) A repeats containing	chr11:120931402	18	166	1	93.58	91.89	0.03	+		+
SREBF2	sterol regulatory element binding transcription factor 2	chr22:40630102	17	998	10	90.43	96.22	-0.09	+		+
STX18	syntaxin 18	chr4:4472892	10	599	8	96.56	90.80	0.09	+		+
SUSD1	sushi domain containing 1	chr9:113860375	14	153	3	88.90	85.16	0.06	+		+
TBC1D4	TBC1 domain family, member 4	chr13:74833767	2	395	3	90.70	84.38	0.10	+		+
TDRD7	tudor domain containing 7	chr9:99235067	4	727	18	89.86	85.90	0.07	+		+
TMEM38A	transmembrane protein 38A	chr19:16652839	3	448	10	96.36	91.41	0.08	+		+
TMOD2	tropomodulin 2 (neuronal)	chr15:49886980	9	971	16	91.99	83.19	0.15	+		+
TSPAN15	tetraspanin 15	chr10:70914230	2	593	4	90.94	85.6	0.09	+		+
ZRANB1	zinc finger, RAN-binding domain containing 1	chr10:126660762	6	375	14	96.83	97.36	-0.01	+		+
ABCA4	ATP-binding cassette, sub-family A (ABC1), member 4	chr1:94267277	30	311	4	100.00	86.36	0.21		+	+
ADAM23	ADAM metallopeptidase domain 23	chr2:207169918	24	788	12	97.23	79.04	0.3		+	+
ADAMTS1	ADAM metallopeptidase with thrombospondin type 1 motif, 1	chr21:27135419	3	628	7	90.43	95.6	-0.08		+	+
AKAP10	A kinase (PRKA) anchor protein 10	chr17:19779217	9	973	16	94.17	88.79	0.08		+	+
APOD	apolipoprotein D	chr3:196786559	2	939	7	90.54	88.64	0.03		+	+
APOD	apolipoprotein D	chr3:196779167	4	269	3	88.9	85.14	0.06		+	+
APOO	apolipoprotein O	chrX:23806801	3	151	4	91.5	83.91	0.13		+	+
APPL1	adaptor protein, phosphotyrosine interaction, PH domain and leucine zipper containing 1	chr3:57257190	9	646	17	90.24	90.73	-0.01		+	+
ARF5	ADP-ribosylation factor 5	chr7:127017298	3	415	5	88.67	85.18	0.06		+	+
ARHGEF37	Rho guanine nucleotide exchange factor (GEF) 37	chr5:148980813	8	594	12	96.36	81.59	0.24		+	+
ARSE	arylsulfatase E (chondrodysplasia punctata 1)	chrX:2885893	3	421	5	90.46	82.86	0.13		+	+

Supplementary Table 4. List of latent 5'SSs activated in gliomas (continued)

Gene symbol	Gene name	Location of latent 5'SS	Intron number	Position of latent 5'SS[a]	Number of stop codons[b]	Latent score[c]	Authentic score[c]	Log score ratio[d]	Up in GBM	Up in ODII	Up in ODIII
ATP5I	ATP synthase, H+ transporting, mitochondrial Fo complex, subunit E	chr4:656496	3	595	11	89.46	90.23	-0.01		+	+
ATP6V1G1	ATPase, H+ transporting, lysosomal 13kDa, V1 subunit G1	chr9:116390777	1	757	10	88.65	90.91	-0.04		+	+
ATP8B1	ATPase, aminophospholipid transporter, class I, type 8B, member 1	chr18:53506173	12	355	5	91.43	94.17	-0.04		+	+
BBS2	Bardet-Biedl syndrome 2	chr16:55093929	8	156	2	91.83	84.32	0.12		+	+
BBS2	Bardet-Biedl syndrome 2	chr16:55090200	12	990	17	92.96	93.76	-0.01		+	+
C10orf27	chromosome 10 open reading frame 27	chr10:72207796	6	522	9	90.22	92.23	-0.03		+	+
C11orf21	chromosome 11 open reading frame 21	chr11:2278710	1	851	5	91.3	94.65	-0.05		+	+
C16orf80	chromosome 16 open reading frame 80	chr16:56706006	5	208	1	90.96	96.56	-0.09		+	+
C2orf29	chromosome 2 open reading frame 29	chr2:101237042	1	671	10	89.19	86.36	0.05		+	+
CA2	carbonic anhydrase II	chr8:86564319	1	724	4	91.5	93.73	-0.03		+	+
CAPZA1	capping protein (actin filament) muscle Z-line, alpha 1	chr1:112999390	5	575	5	90.97	67.27	0.44		+	+
CAPZA2	capping protein (actin filament) muscle Z-line, alpha 2	chr7:116331935	5	263	5	90.53	86.16	0.07		+	+
CCDC11	coiled-coil domain containing 11	chr18:46042010	2	347	11	89.46	86.74	0.04		+	+
CDH1	cadherin 1, type 1, E-cadherin (epithelial)	chr16:67402567	6	823	9	90.97	86.61	0.07		+	+
CHUK	conserved helix-loop-helix ubiquitous kinase	chr10:101943324	18	406	10	90.50	82.92	0.13		+	+
CHUK	conserved helix-loop-helix ubiquitous kinase	chr10:101939929	20	686	13	91.43	94.17	-0.04		+	+
CLMN	calmin (calponin-like, transmembrane)	chr14:94750767	5	963	16	89.96	97.23	-0.11		+	+
COMMD9	COMM domain containing 9	chr11:36267325	1	162	1	89.01	98.87	-0.15		+	+
COPG	coatomer protein complex, subunit gamma	chr3:130462726	12	387	4	90.77	85.23	0.09		+	+
CPD	carboxypeptidase D	chr17:25795616	11	502	17	97.7	93.76	0.06		+	+
CSRP1	cysteine and glycine-rich protein 1	chr1:199720679	5	354	7	95.18	93.27	0.03		+	+
CUL7	cullin 7	chr6:43125476	6	202	1	88.7	83.87	0.08		+	+
DEK	DEK oncogene	chr6:18334083	10	300	6	96.22	90.36	0.09		+	+
DLAT	dihydrolipoamide S-acetyltransferase	chr11:111437963	13	856	15	92.96	94.63	-0.03		+	+
DNAH17	dynein, axonemal, heavy chain 17	chr17:74074569	6	106	1	88.65	86.62	0.03		+	+

Supplementary Table 4. List of latent 5'SSs activated in gliomas (continued)

Gene symbol	Gene name	Location of latent 5'SS	Intron number	Position of latent 5'SS[a]	Number of stop codons[b]	Latent score[c]	Authentic score[c]	Log score ratio[d]	Up in GBM	Up in ODII	Up in ODIII
DPM2	dolichyl-phosphate mannosyltransferase polypeptide 2, regulatory subunit	chr9:129738210	3	442	3	88.72	91.36	-0.04		+	+
DYNC1LI2	dynein, cytoplasmic 1, light intermediate chain 2	chr16:65325150	6	471	5	90.9	70.18	0.37		+	+
ETFDH	electron-transferring-flavoprotein dehydrogenase	chr4:159821879	2	671	19	88.64	98.87	-0.16		+	+
FBXO27	F-box protein 27	chr19:44213910	2	433	2	99.13	90.5	0.13		+	+
FKBP3	FK506 binding protein 3, 25kDa	chr14:44656393	7	587	18	92.83	76.91	0.27		+	+
FLOT2	flotillin 2	chr17:24234989	3	379	3	88.91	84.84	0.07		+	+
FTO	fat mass and obesity associated	chr16:52480911	7	548	7	91.30	93.13	-0.03		+	+
GGCX	gamma-glutamyl carboxylase	chr2:85633231	9	341	10	89.46	89.94	-0.01		+	+
GIMAP4	GTPase, IMAP family member 4	chr7:149898027	2	48	1	92.64	90.53	0.03		+	+
GPATCH3	G patch domain containing 3	chr1:27091171	6	317	5	92.11	93.58	-0.02		+	+
GPR143	G protein-coupled receptor 143	chrX:9673932	5	151	2	89.48	92.54	-0.05		+	+
HERC1	hect (homologous to the E6-AP (UBE3A) carboxyl terminus) domain and RCC1 (CHC1)-like domain (RLD) 1	chr15:61842374	3	973	23	90.50	91.96	-0.02		+	+
HERC1	hect (homologous to the E6-AP (UBE3A) carboxyl terminus) domain and RCC1 (CHC1)-like domain (RLD) 1	chr15:61795204	22	349	4	90.92	85.6	0.09		+	+
HIF1AN	hypoxia inducible factor 1, alpha subunit inhibitor	chr10:102290727	3	199	4	93.27	96.1	-0.04		+	+
HNRPUL2	heterogeneous nuclear ribonucleoprotein U-like 2	chr11:62249865	1	801	23	91.02	91.99	-0.02		+	+
HSD17B3	hydroxysteroid (17-beta) dehydrogenase 3	chr9:98052773	5	747	9	89.53	85.52	0.07		+	+
IKZF5	IKAROS family zinc finger 5 (Pegasus)	chr10:124747529	3	469	9	90.36	83.34	0.12		+	+
ILF3	interleukin enhancer binding factor 3, 90kDa	chr19:10659634	1	251	3	88.72	83.38	0.09		+	+
IMPAD1	inositol monophosphatase domain containing 1	chr8:58040350	4	953	17	90.96	89.37	0.03		+	+
IRAK2	interleukin-1 receptor-associated kinase 2	chr3:10243485	10	369	6	91.43	93.53	-0.03		+	+
ISCU	iron-sulfur cluster scaffold homolog (E. coli)	chr12:107484233	4	898	16	89.9	92.83	-0.05		+	+
ITGA2	integrin, alpha 2 (CD49B, alpha 2 subunit of VLA-2 receptor)	chr5:52377449	4	771	20	89.67	84.62	0.08		+	+
JAM3	junctional adhesion molecule 3	chr11:133516204	3	330	6	94.73	85.93	0.14		+	+

Supplementary Table 4. List of latent 5'SSs activated in gliomas (continued)

Gene symbol	Gene name	Location of latent 5'SS	Intron number	Position of latent 5'SS[a]	Number of stop codons[b]	Latent score[c]	Authentic score[c]	Log score ratio[d]	Up in GBM	Up in ODII	Up in ODIII
JAM3	junctional adhesion molecule 3	chr11:133521452	6	303	10	88.65	82.02	0.11		+	+
KAT2B	K(lysine) acetyltransferase 2B	chr3:20117279	4	830	12	88.61	89.77	-0.02		+	+
KIAA0368	KIAA0368	chr9:113242214	7	258	12	90.5	90.49	0		+	+
KIAA0657	KIAA0657	chr2:220129907	3	881	8	91.5	94.43	-0.05		+	+
KIAA0776	KIAA0776	chr6:97078783	2	896	17	91.78	88.4	0.05		+	+
KIAA1984	KIAA1984	chr9:138821487	13	149	1	89.18	76.24	0.23		+	+
KPNB1	karyopherin (importin) beta 1	chr17:43091495	5	471	8	89.63	97.82	-0.13		+	+
KPNB1	karyopherin (importin) beta 1	chr17:43101467	10	693	11	91.33	88.9	0.04		+	+
LBP	lipopolysaccharide binding protein	chr20:36413711	3	956	8	91.43	86.86	0.07		+	+
LLGL1	lethal giant larvae homolog 1 (Drosophila)	chr17:18070439	1	638	2	90.99	90.36	0.01		+	+
LRIG2	leucine-rich repeats and immunoglobulin-like domains 2	chr1:113444785	11	153	4	89.65	86.16	0.06		+	+
LTV1	LTV1 homolog (S. cerevisiae)	chr6:144209747	3	694	17	96.10	86.88	0.15		+	+
MAN2A2	mannosidase, alpha, class 2A, member 2	chr15:89252475	7	747	8	88.79	84.12	0.08		+	+
MAOB	monoamine oxidase B	chrX:43545432	5	930	20	89.14	84.28	0.08		+	+
MAP3K5	mitogen-activated protein kinase kinase kinase 5	chr6:137001733	13	640	22	89.86	85.91	0.06		+	+
MAPK4	mitogen-activated protein kinase 4	chr18:46507031	5	489	11	90.5	82.79	0.13		+	+
MCM3APAS	MCM3APAS	chr21:46486182	3	822	12	95.18	86.16	0.14		+	+
MRPS15	mitochondrial ribosomal protein S15	chr1:36701405	2	588	18	90.97	92.51	-0.02		+	+
MTFMT	mitochondrial methionyl-tRNA formyltransferase	chr15:63095555	6	271	9	92.96	87.08	0.09		+	+
N4BP2	NEDD4 binding protein 2	chr4:39831709	16	792	24	88.59	99.13	-0.16		+	+
NACC2	NACC family member 2, BEN and BTB (POZ) domain containing	chr9:138047184	3	747	7	90.43	97.36	-0.11		+	+
NDUFS6	NADH dehydrogenase (ubiquinone) Fe-S protein 6, 13kDa (NADH-coenzyme Q reductase)	chr5:1855819	2	332	5	91.02	96.95	-0.09		+	+
NINJ2	ninjurin 2	chr12:544903	2	473	4	89.71	88.27	0.02		+	+
NOC3L	nucleolar complex associated 3 homolog (S. cerevisiae)	chr10:96107513	3	316	9	90.58	84.66	0.10		+	+
NPHS2	nephrosis 2, idiopathic, steroid-resistant (podocin)	chr1:177792159	5	625	18	92.98	84.83	0.13		+	+
NR2F1	nuclear receptor subfamily 2, group F, member 1	chr5:92950886	2	981	11	90.06	97.36	-0.11		+	+
P2RX1	purinergic receptor P2X, ligand-gated ion channel, 1	chr17:3753700	5	270	5	92.69	83.02	0.16		+	+

Supplementary Table 4. List of latent 5'SSs activated in gliomas (continued)

Gene symbol	Gene name	Location of latent 5'SS	Intron number	Position of latent 5'SS[a]	Number of stop codons[b]	Latent score[c]	Authentic score[c]	Log score ratio[d]	Up in GBM	Up in ODII	Up in ODIII
PAFAH1B2	platelet-activating factor acetylhydrolase 1b, catalytic subunit 2 (30kDa)	chr11:116540099	5	282	2	90.97	84.98	0.1		+	+
PEX1	peroxisomal biogenesis factor 1	chr7:91983866	5	659	14	89.01	81.71	0.12		+	+
PEX3	peroxisomal biogenesis factor 3	chr6:143838422	9	737	16	91	92.94	-0.03		+	+
PFDN2	prefoldin subunit 2	chr1:159337462	3	999	24	90.96	96.83	-0.09		+	+
PIGX	phosphatidylinositol glycan anchor biosynthesis, class X	chr3:197939447	4	44	1	91.17	96.22	-0.08		+	+
PIM2	pim-2 oncogene	chrX:48657033	4	207	4	92.66	92.4	0		+	+
PMM1	phosphomannomutase 1	chr22:40310077	4	153	2	92.64	90.92	0.03		+	+
POLR2C	polymerase (RNA) II (DNA directed) polypeptide C, 33kDa	chr16:56055211	2	721	12	91.43	93.3	-0.03		+	+
PPIH	peptidylprolyl isomerase H (cyclophilin H)	chr1:42904466	7	142	3	90.46	90.91	-0.01		+	+
PRDM4	PR domain containing 4	chr12:106658312	10	540	10	88.65	100	-0.17		+	+
PSMD5	proteasome 26S non-ATPase subunit 5	chr9:122630775	5	422	12	89.93	93.2	-0.05		+	+
RAB15	RAB15, member RAS onocogene family	chr14:64485975	5	820	8	88.65	100	-0.17		+	+
RAB22A	RAB22A, member RAS oncogene family	chr20:56352603	3	343	4	93.76	88.67	0.08		+	+
RBP1	retinol binding protein 1, cellular	chr3:140740757	1	234	2	92.23	89.04	0.05		+	+
ROCK2	Rho-associated, coiled-coil containing protein kinase 2	chr2:11255699	25	368	7	92.64	86.98	0.09		+	+
RPA1	replication protein A1, 70kDa	chr17:1742818	15	835	10	91.09	87.96	0.05		+	+
RYR3	ryanodine receptor 3	chr15:31610652	4	494	9	91.64	91.87	0.00		+	+
SCCPDH	saccharopine dehydrogenase (putative)	chr1:244970860	4	635	22	91.43	86	0.09		+	+
SHISA4	shisa homolog 4 (Xenopus laevis)	chr1:200126174	2	808	10	95.23	94.41	0.01		+	+
SKAP2	src kinase associated phosphoprotein 2	chr7:26732626	7	399	14	88.64	74.68	0.25		+	+
SKAP2	src kinase associated phosphoprotein 2	chr7:26695785	10	643	13	88.59	90.06	-0.02		+	+
SLC25A12	solute carrier family 25 (mitochondrial carrier, Aralar), member 12	chr2:172399168	7	314	6	90.04	91.86	-0.03		+	+
SLC25A20	solute carrier family 25 (carnitine/acylcarnitine translocase), member 20	chr3:48870756	8	187	2	95.18	92.69	0.04		+	+
SLC31A2	solute carrier family 31 (copper transporters), member 2	chr9:114960588	2	695	10	99.13	92.54	0.1		+	+

Supplementary Table 4. List of latent 5'SSs activated in gliomas (continued)

Gene symbol	Gene name	Location of latent 5'SS	Intron number	Position of latent 5'SS[a]	Number of stop codons[b]	Latent score[c]	Authentic score[c]	Log score ratio[d]	Up in GBM	Up in ODII	Up in ODIII
SLC5A6	solute carrier family 5 (sodium-dependent vitamin transporter), member 6	chr2:27278657	12	527	8	88.65	89.06	-0.01		+	+
SLC6A5	solute carrier family 6 (neurotransmitter transporter, glycine), member 5	chr11:20582977	3	432	10	95.18	90.12	0.08		+	+
SLC9A7	solute carrier family 9 (sodium/hydrogen exchanger), member 7	chrX:46386723	12	891	17	89.3	85.63	0.06		+	+
SORL1	sortilin-related receptor, L(DLR class) A repeats containing	chr11:120927512	16	924	22	91.41	97.23	-0.09		+	+
SORL1	sortilin-related receptor, L(DLR class) A repeats containing	chr11:120946711	23	523	11	91.55	82.87	0.14		+	+
SORT1	sortilin 1	chr1:109670448	13	688	7	93.27	80.28	0.22		+	+
SORT1	sortilin 1	chr1:109660155	17	839	14	90.46	94.07	-0.06		+	+
SPTBN5	spectrin, beta, non-erythrocytic 5	chr15:39931863	61	260	5	89.25	87.41	0.03		+	+
SPTY2D1	SPT2, Suppressor of Ty, domain containing 1 (S. cerevisiae)	chr11:18611813	1	507	6	88.78	95.74	-0.11		+	+
SRMS	src-related kinase lacking C-terminal regulatory tyrosine and N-terminal myristylation sites	chr20:61643600	5	359	2	92.26	85.60	0.11		+	+
SS18L1	synovial sarcoma translocation gene on chromosome 18-like 1	chr20:60167668	2	470	5	91.64	85.6	0.1		+	+
STAM	signal transducing adaptor molecule (SH3 domain and ITAM motif) 1	chr10:17726811	1	428	3	93.83	90.91	0.05		+	+
SUSD1	sushi domain containing 1	chr9:113958762	2	838	13	89.46	90.91	-0.02		+	+
TACO1	translational activator of mitochondrially encoded cytochrome c oxidase I	chr17:59032704	1	251	2	93.96	95.23	-0.02		+	+
TACO1	translational activator of mitochondrially encoded cytochrome c oxidase I	chr17:59038283	3	752	12	88.59	91.36	-0.04		+	+
TEC	tec protein tyrosine kinase	chr4:47834689	17	749	18	97.10	82.66	0.23		+	+
TFB2M	transcription factor B2, mitochondrial	chr1:244786146	4	351	7	91.55	94.07	-0.04		+	+
TMEM199	transmembrane protein 199	chr17:23709503	1	473	7	90.68	90.41	0.00		+	+
TMEM63A	transmembrane protein 63A	chr1:224115771	13	769	9	91.53	87.35	0.07		+	+
TMEM63A	transmembrane protein 63A	chr1:224106592	20	395	4	88.65	90.04	-0.02		+	+
TOP2B	topoisomerase (DNA) II beta 180kDa	chr3:25639825	21	184	6	97.82	86	0.19		+	+
TPR	translocated promoter region (to activated MET oncogene)	chr1:184586600	20	484	11	91.28	88.51	0.04		+	+

Supplementary Table 4. List of latent 5'SSs activated in gliomas (continued)

Gene symbol	Gene name	Location of latent 5'SS	Intron number	Position of latent 5'SS[a]	Number of stop codons[b]	Latent score[c]	Authentic score[c]	Log score ratio[d]	Up in GBM	Up in ODII	Up in ODIII
TRMT1L	TRM1 tRNA methyltransferase 1-like	chr1:183363652	11	771	15	88.65	87.2	0.02		+	+
TRMT6	tRNA methyltransferase 6 homolog (S. cerevisiae)	chr20:5870257	8	339	6	90.96	84.63	0.1		+	+
TTC38	tetratricopeptide repeat domain 38	chr22:45056709	7	431	7	93.2	90.17	0.05		+	+
TUSC2	tumor suppressor candidate 2	chr3:50339929	1	459	9	90.94	93.99	-0.05		+	+
UBASH3B	ubiquitin associated and SH3 domain containing B	chr11:122173224	9	274	2	89.10	91.36	-0.04		+	+
UHRF1BP1	UHRF1 binding protein 1	chr6:34913062	8	960	20	94.18	84.06	0.16		+	+
UNKL	unkempt homolog (Drosophila)-like	chr16:1404176	1	440	1	89.55	89.10	0.01		+	+
USP9Y	ubiquitin specific peptidase 9, Y-linked	chrY:13462235	36	302	7	90.81	97.7	-0.11		+	+
UTP18	UTP18, small subunit (SSU) processome component, homolog (yeast)	chr17:46720948	11	386	6	91.78	83.45	0.14		+	+
VAMP3	vesicle-associated membrane protein 3 (cellubrevin)	chr1:7760058	3	94	2	92.23	90.69	0.02		+	+
VRK1	vaccinia related kinase 1	chr14:96412901	12	692	20	90.17	85.92	0.07		+	+
WDFY2	WD repeat and FYVE domain containing 2	chr13:51229121	10	523	6	89.25	87.54	0.03		+	+
WDR66	WD repeat domain 66	chr12:120881684	14	302	5	94.07	85.11	0.14		+	+
WIPF3	WAS/WASL interacting protein family, member 3	chr7:29895191	5	834	5	90.73	90.91	0		+	+
ZAR1	zygote arrest 1	chr4:48188716	1	689	8	90.36	90.77	-0.01		+	+
ZFP37	zinc finger protein 37 homolog (mouse)	chr9:114858030	1	627	10	90.69	89.92	0.01		+	+
ZMYND12	zinc finger, MYND-type containing 12	chr1:42670467	7	933	21	88.72	85.67	0.05		+	+
ZNF175	zinc finger protein 175	chr19:56768873	2	408	3	90.36	88.06	0.04		+	+
ZYG11B	zyg-11 homolog B (C. elegans)	chr1:53052513	12	342	4	88.65	91.83	-0.05		+	+

[a]Number of nt downstream from the authentic 5'SS.
[b]Number of in frame stop codons within the latent exon.
[c]See Methods.
[d]Latent score divided by authentic score.

Supplementary Table 5. Primer sequences

Primer name	Sequence
β-actin fwd	CTGGAACGGTGAAGGTGACA
β-actin rev	AAGGGACTTCCTGTAACAATGCA
C1R b	ACAGCAGATCCACAGCATTG
C1R c	GTCCTACCCCTGAAAGACCC
CAD a	CTCAGATCCTGGTCGACGGC
CAD b	CAGGGAGCCGCACCAGTTTC
CAD c	CCGGGTGCAGGAGTGACAGC
CAD Pre	AGAGAGGAGTTTACGTTGGGAGG
CAD Aut	ACCAAACTCTTCGTGGAGGCC
CAD Lat #2	CCAGAGGCGGAGAGGCCC
DERL2 b	CAAGAAAACTAAGCTCACAA
DERL2 c	GTATCCTTCAACAAACAACA
GAPDH fwd	TGCACCACCAACTGCTTAGC
GAPDH rev	GGCATGGACTGTGGTCATGAG
GTPBP4 a	GACTCTCTCTACCGCTGCAA
GTPBP4 b	AAGCTGGACTTCCCAACATT
GTPBP4 c2	GACAGGGTCAGTTATTCTTCCT
LPCAT4 b	ACAATGGGGTCAAAGAAAGT
LPCAT4 c	CAGGATGGAGGAAGTAAGTG
PSMD8 a	GCATGTACGAGCAACTCAAG
PSMD8 b	GAATTAGCTGCTGTTTGGTCA
PSMD8 c	GGGTCGACTCAAGGTAAAGT
M6PR b	ATCCTGCCAGAAGGCTAAGT
M6PR c	CACGTGAGTATGCCTTCCTT
TMED10 b	CCAGGTAGCTAGTCCAATGA
TMED10 c2	CCAAGGTGGGTATATGGAG
VIPR2 b	AACGTCTCTGACCATCCGTC
VIPR2 c	AAAGCAGGTACTGTCCGTCG

Supplementary Table 6. RT-PCR annealing temperatures

Primer-pair	Annealing (°C)
β-actin	60
C1R b+c	59
CAD a+b	63
CAD b+c	62
DERL2 b+c	55
GAPDH	60
GFP	59
GTPBP4 a+b	57
GTPBP4 b+c2	55
LPCAT4 b+c	56
M6PR b+c	51
PSMD8 a+b	54
PSMD8 b+c	57
TEMD10 b+c2	55
VIPR2 b+c	56
